# Supplementary material for: Discontinuation, persistence and adherence to subcutaneous biologics delivered via a homecare route to Scottish adults with rheumatic diseases: a retrospective study
Source: BMJ Open. 2019 Sep 4;9(9):e027059. doi: 10.1136/bmjopen-2018-027059 (PMC6731860; doi:10.1136/bmjopen-2018-027059)
Supplement: Supplementary data [file bmjopen-2018-027059supp001.pdf]

Supplementary file :

Validation method and results

Of the 751 patients in HSD, 340 were also in the RAD. A minority of these (n=48) had diagnosis recorded in either of the datasets with the majority of these (n=292) with diagnosis recorded in both datasets (supplementary figure 1). RAD diagnosis was considered as the "gold standard" because it was collected in rheumatology clinics by clinical staff and was used to determine the validity of HSD diagnosis in the 292 patients. There were 3540 patients diagnosed with the three rheumatic conditions (RA, PsA or AS) who attended rheumatology clinics in Glasgow and were only recorded in RAD. These patients were not used for the validation study.

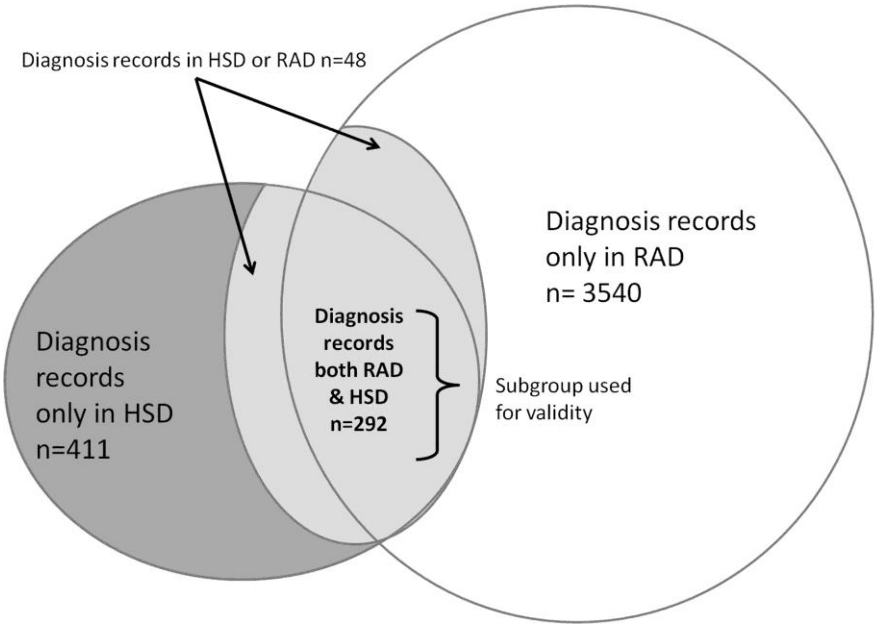

Supplementary Figure 1. Diagnoses of rheumatic conditions according to HSD and RAD records.

Validity of diagnoses of rheumatic conditions (PPV and sensitivity)

The positive predictive value (PPV) and sensitivity with 95% confidence intervals were calculated for the diagnosis of RA, PsA or AS in HSD. PPV was calculated as the proportion of subjects in HSD with an indication for any of these three conditions in first recorded delivery and confirmed diagnosis in RAD. Sensitivity was calculated as the proportion of RAD cases who had a RA, PsA or AS diagnosis from all the RA, PsA or AS patients in the 292 subjects. A high PPV identifies a true population, or this case subgroup, of patients with chronic inflammatory diseases; while a high sensitivity allows to asses as many cases as possible.

Validity of arthritic conditions

From the subset of patients with arthritic conditions in HSD, the diagnosis was confirmed in 292 patients, with a 91.8%(95%CI: 86.4-95.1) PPV and a sensitivity of 97.3% (95%CI:93.3-99.0) for RA, 97.4% (95%CI:86.5-99.9) PPV and 94.9% (95%CI: 83.1-98.6)sensitivity for AS and 95.8% (95%CI:89.8-98.4) PPV and 88.5%(95%CI:80.9-93.3) sensitivity for PsA.

Many studies focus in the optimal clinical use and safety of medication in rheumatic diseases using administrative or claims data and one of the first requirements, following the best-practice consensus from the CANRAD Network, is the validation of case definitions.[1] There are mainly two types of validation methods (a) internal using algorithms or sensitivity analyses within the data or (b) external using data from other sources such as clinical notes or data captured by general practitioners.[2] In this study, an external validation was performed by comparing the diagnoses of the patients present in both HSD and RAD databases. Both the PPV and sensitivity were above 80% for the three diseases. In contrast to several studies, instead of using ICD-10 codes, our study used free text entered by clinicians in RAD which was compared to the diagnoses entered from the prescriptions in HSD. All the patients also had at least one prescription for a subcutaneous biologic in HSD. Our sample size (n=292) is in the range of similar studies aimed to validate diagnoses (n=85-450), and our PPV values and sensitivities are comparable to others using data from rheumatology clinics (PPV 95-97%; sensitivities reported 30-100%).[3, 4]

Analyses with incident users of subcutaneous biologics

Incident users were defined as patients who did not receive a subcutaneous biologic in the first six months of the study period (from January-June 2012). This incident cohort included 673 patients (89.6% from the original cohort), from them 95 had AS, 208 PsA and 370 RA (Supplementary Table 1). The proportion of patients according to age, gender and the distribution of socioeconomic levels (SIMD) were similar.

A total of 88.9% of incident patients had more than one delivery of biologic during the study period (compared to 89.3% from the original cohort) and the same percentage of 83.2% did not switch biologic.

The index biologics mostly used were also adalimumab and etanercept. Although the proportion of users according to their index biologics did not change, there were less users of adalimumab, certolizumab pegol and etanercept in the incident cohort as the prevalent users of these three medications were excluded. This is in agreement with the dates of approval for their use as these biologics were the first ones approved and patients were more likely to be users before 2012.

The pattern of concomitant medications prevailed in the incident cohort as RA and PsA patients were frequent users of oral methotrexate, DMARDs and NSAIDs, while AS patients mainly used NSAIDs.

Supplementary Table 1. Baseline characteristics of incident patients with rheumatic conditions receiving subcutaneous biologic from July 2012-May 2015 (n=673)

**Rheumatic disease**	**All**		**Ankylosing spondylitis**		**Psoriatic arthritis**		**Rheumatoid arthritis**		**p-value**
n	**673**		**95**		**208**		**370**		
**Female - n (%)**	**446**	(66.3)	**34**	(36.8)	**117**	(56.3)	**295**	(79.7)	$<2.2 \times 10^{-16}$
**Age***									
Age (median, IQR)*	**52.9(42.5-60.4)**		**47.4 (35.6-55.4)**		**48.0(40.1-57.3)**		**55.9(47.1-64.2)**		1.78×10^{-11}
**Age by category**									4.6×10^{-10}
**18-34 (%)**	**87**	(12.9)	**21**	(22.1)	**34**	(16.3)	**32**	(8.6)	
**35-49 (%)**	**196**	(29.1)	**31**	(32.6)	**83**	(39.9)	**82**	(22.2)	
**50-64 (%)**	**282**	(41.9)	**35**	(36.8)	**75**	(36.1)	**172**	(46.5)	
**65+ (%)**	**108**	(16.0)	**8**	(8.4)	**16**	(7.7)	**84**	(22.7)	
SIMD †									0.314
**1 Most deprived (%)**	**274**	(40.7)	**38**	(43.2)	**85**	(42.1)	**151**	(41.8)	
**2 (%)**	**117**	(17.4)	**19**	(21.6)	**40**	(19.8)	**58**	(16.1)	
**3 (%)**	**75**	(11.1)	**13**	(14.8)	**22**	(10.9)	**40**	(11.1)	
**4 (%)**	**78**	(11.6)	**12**	(13.6)	**22**	(10.9)	**44**	(12.2)	
**5 Least deprived (%)**	**107**	(15.9)	**6**	(6.8)	**33**	(16.3)	**68**	(18.8)	
Number of deliveries of biologics									
Number of deliveries of biologics (median, IQR)	**6**	(3-10)	**6**	(3-11)	**6**	(3-11)	**5**	(3-10)	0.400

Number of deliveries of biologics by category (%)									0.803
1	**75**	(11.1)	**13**	(13.7)	**18**	(8.7)	**44**	(11.9)	
2-10	**434**	(64.5)	**57**	(60.0)	**136**	(65.4)	**241**	(65.1)	
11-20	**129**	(19.2)	**19**	(20.0)	**43**	(20.7)	**67**	(18.1)	
21+	**35**	(5.2)	**6**	(6.3)	**11**	(5.3)	**18**	(4.9)	
Number of different biologics delivered (%)									
1	**560**	(83.2)	**81**	(85.3)	**165**	(79.3)	**314**	(84.9)	0.196
2, 3 or 4 (switchers)	**113**	(16.8)	**14**	(14.7)	**43**	(20.7)	**56**	(15.1)	
First biologic delivered(%)									
									$7.33x10^{-11}$
**Adalimumab**	**263**	(39.1)	**47**	(49.5)	**98**	(47.1)	**118**	(31.9)	
**Certolizumab Pegol**	**68**	(10.1)	**6**	(6.3)	**<5**		**60**	(16.2)	
**Etanercept**	**215**	(31.9)	**22**	(23.2)	**77**	(37.0)	**116**	(31.4)	
**Golimumab**	**91**	(13.5)	**20**	(21.1)	**24**	(11.5)	**47**	(12.7)	
**Abatacept**	**8**	(1.2)	**NA**		**NA**		**8**	(2.2)	NA[‡]
**Tocilizumab**	**21**	(3.1)	**NA**		**NA**		**21**	(5.7)	
**Ustekinumab**	**7**	(1.0)	**NA**		**7**	(3.4)	**NA**		
**Follow-up (days), *(median, IQR)***	**480**	**(264.0-734.0)**	**442 (278-749.5)**		**504.5(267.5-763.5)**		**482.0 (263.3-731.5)**		0.950

Concomitant medication use within one year before index date (%)									
**Antibacterial use**	**20**	(3.0)	**<5**		**<5**		**14**	(3.8)	0.149
**Oral methotrexate use**	**39**	(5.8)	**<5**		**9**	(4.3)	**29**	(7.8)	
**Any DMARD**	**54**	(8.0)	**<5**		**13**	(6.3)	**40**	(10.8)	
**NSAID**	**54**	(8.0)	**8**	(8.4)	**15**	(7.2)	**31**	(8.4)	
**Antifungal use**	**<5**		**NA**		**NA**		**<5**		NA‡
**Antiviral use**	**NA**		**NA**		**NA**		**<5**		
**Subcutaneous methotrexate use**	**NA**		**NA**		**NA**		**NA**		
**Gluco or corticosteroids**	**10**	(2.5)	**NA**		**<5**		**9**	(2.4)	
Concomitant medication use during study period (%)									1.0×10^{-7}
**Antibacterial use**	**394**	(58.5)	**46**	(48.4)	**124**	(59.6)	**224**	(60.5)	
**Antifungal use**	**62**	(9.2)	**7**	(7.4)	**22**	(10.6)	**33**	(8.9)	
**Antiviral use**	**44**	(6.5)	**<5**		**16**	(7.7)	**26**	(7.0)	
**Oral methotrexate use**	**284**	(42.2)	**7**	(7.4)	**78**	(37.5)	**199**	(53.8)	
**Any DMARD**	**432**	(64.2)	**9**	(9.5)	**122**	(58.7)	**301**	(81.4)	
**NSAID**	**430**	(63.9)	**65**	(68.4)	**137**	(65.9)	**228**	(61.6)	
**Gluco or corticosteroids**	**132**	(19.6)	**10**	(10.5)	**26**	(12.5)	**96**	(25.9)	
**Subcutaneous methotrexate use**	**10**	(1.5)	**NA**		**<5**		**7**	(1.9)	NA‡

**Comorbidities (%)**§									$<2.2x10^{-16}$
Charlson score									
0	**145**	(21.5)	**32**	(30.5)	**66**	(31.7)	**47**	(12.7)	
1	**164**	(24.4)	..		..		**143**	(38.6)	
2+	**33**	(4.9)	..		..		**27**	(7.3)	
unknown	**331**	(49.2)	**55**	(52.4)	**123**	(59.1)	**153**	(41.4)	

*Age at first delivery, † from 673 patients 22 did not have Scottish Index of Multiple Deprivation (SIMD) values. Follow-up is from first delivery to study end date. NA‡ Comparison was not calculable because numbers in some cells were not available, were zero and/or too small. § Following the statistical disclosure protocol of the Greater Glasgow and Clyde Safe Haven to avoid attribute disclosure, the cell suppression (primary and secondary) method was used for for values in the Comorbidities section as some were too small. DMARD- disease-modifying anti-rheumatic drugs; NSAID- non-steroidal anti-inflammatory drug

Discontinuation and persistence

Also in the incident cohort approximately half of the patients were persistent on their index biologic throughout the study period (55% AS, 55% PsA, 49%RA) as shown in Supplementary Figure 2. From those who discontinue treatment the majority reintiate it with their index biologic (18%AS, 18% PsA, 19%RA) and a few switch to other biologics (5%AS, 3% PsA, 1%RA).

Supplementary Figure 2. Persistence and discontinuation (re-initiating or ceasing) of treatment with biologics according to rheumatic condition in the incident cohort (n=673)

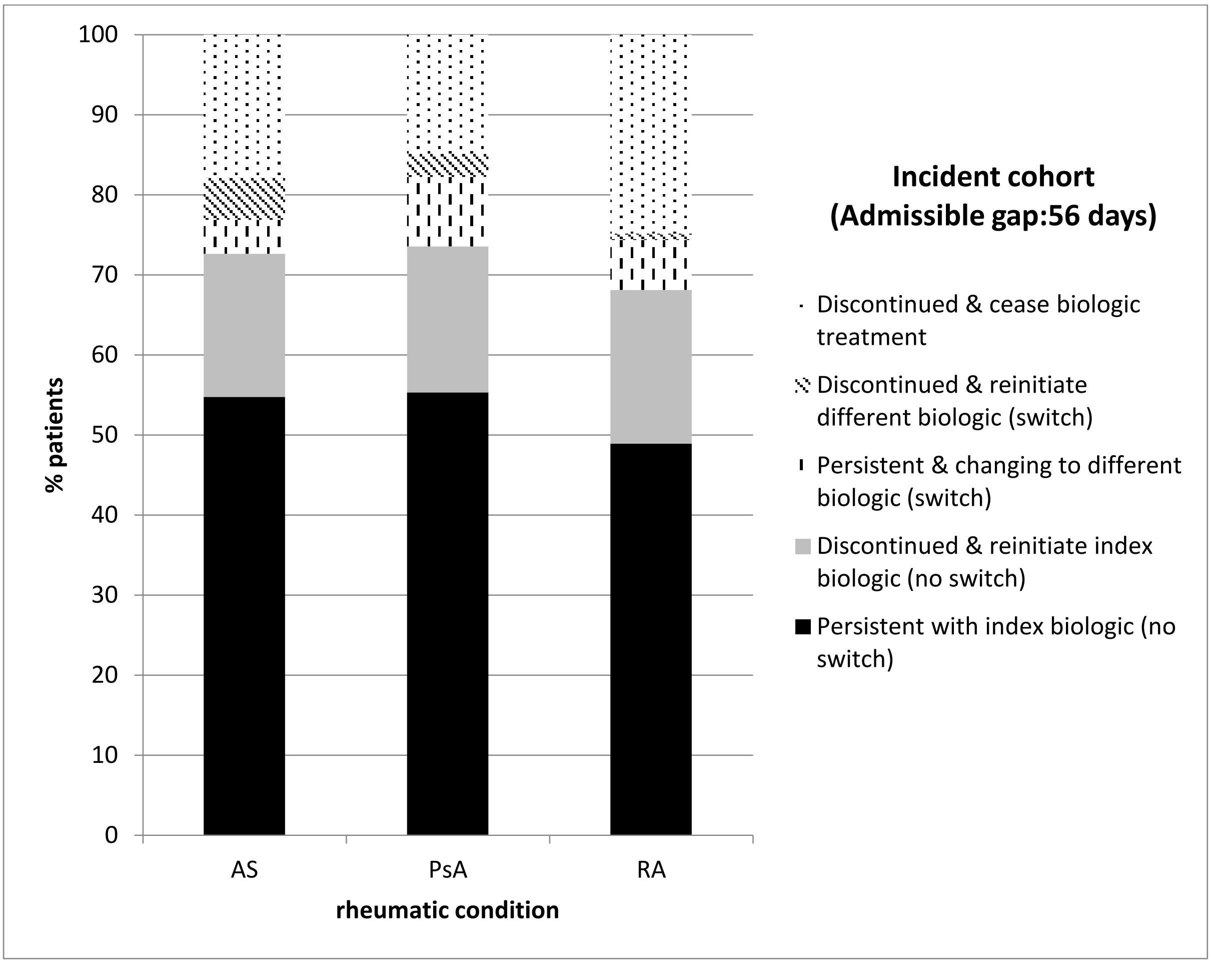

Sensitivity analyses discontinuation and persistence

Supplementary Figure 3. Persistence and discontinuation (re-initiating or ceasing) of treatment with biologics according to rheumatic condition in all the cohort with a gap of 28 days (Fig.6A) or 84 days (Fig.6B) and in the incident cohort with a gap of 28 days (Fig.6C) or 84 days (Fig.6D)

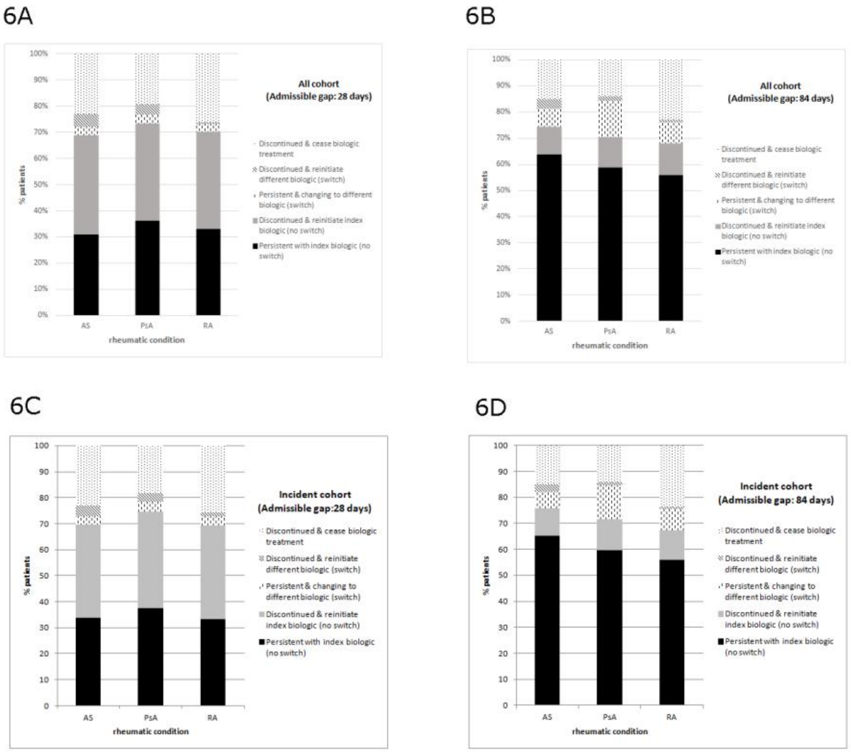

The crude survival curve of *time to discontinuation* (first gap greater than 56 days between biologic deliveries) of any biologic in the incident is shown in Supplementary Figure 4. In the crude model, the proportion of patients persisting is also slightly higher in PsA and AS compared to RA; however, there is no significant difference according to the log-rank test (p=0.165) and the Cox regression between RA and PsA (HR =0.77, 95%CI:0.58-1.01, p=0.0597).

Supplementary Figure 4:Crude survival curves comparing persistent patients according to rheumatic condition. (n=577)

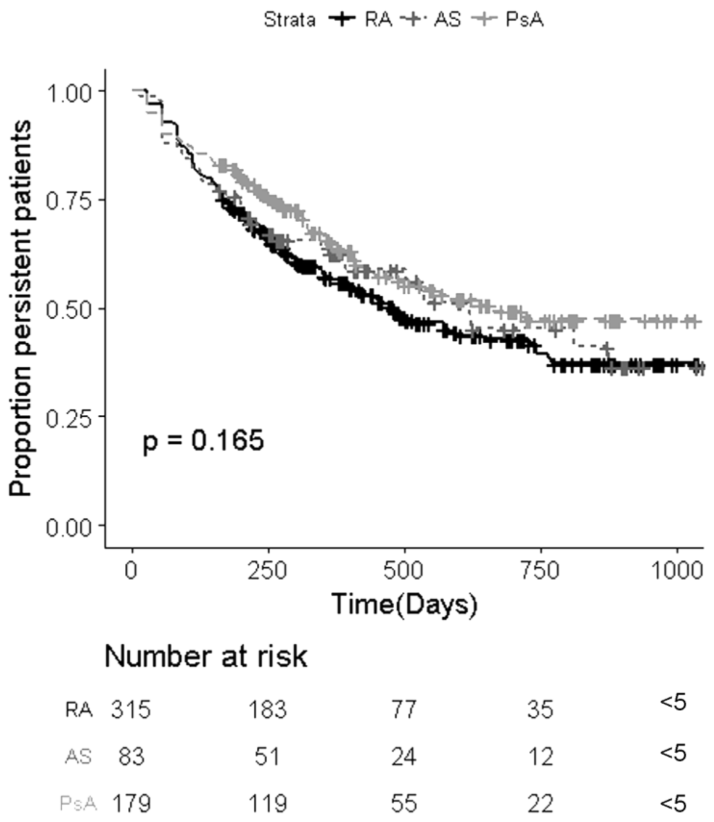

After adjustment for covariates, the difference between RA and PsA patients was still present (HR =0.73, 95%CI:0.54-1.00, p=0.048). Fewer covariates were significant in the incident cohort model. SIMD 3 was associated with a higher risk of discontinuation, whereas SIMD 5 (least deprived quintile) was associated with a lower risk of discontinuation as shown in Supplementary Figure 5. Sex, age and the use of methotrexate or DMARD prior or during the study were not statistically significant covariates.

Supplementary Figure 5: Adjusted Hazard Ratios for persistence with biologics using Cox regression analysis excluding patients without SIMD values (n=556)

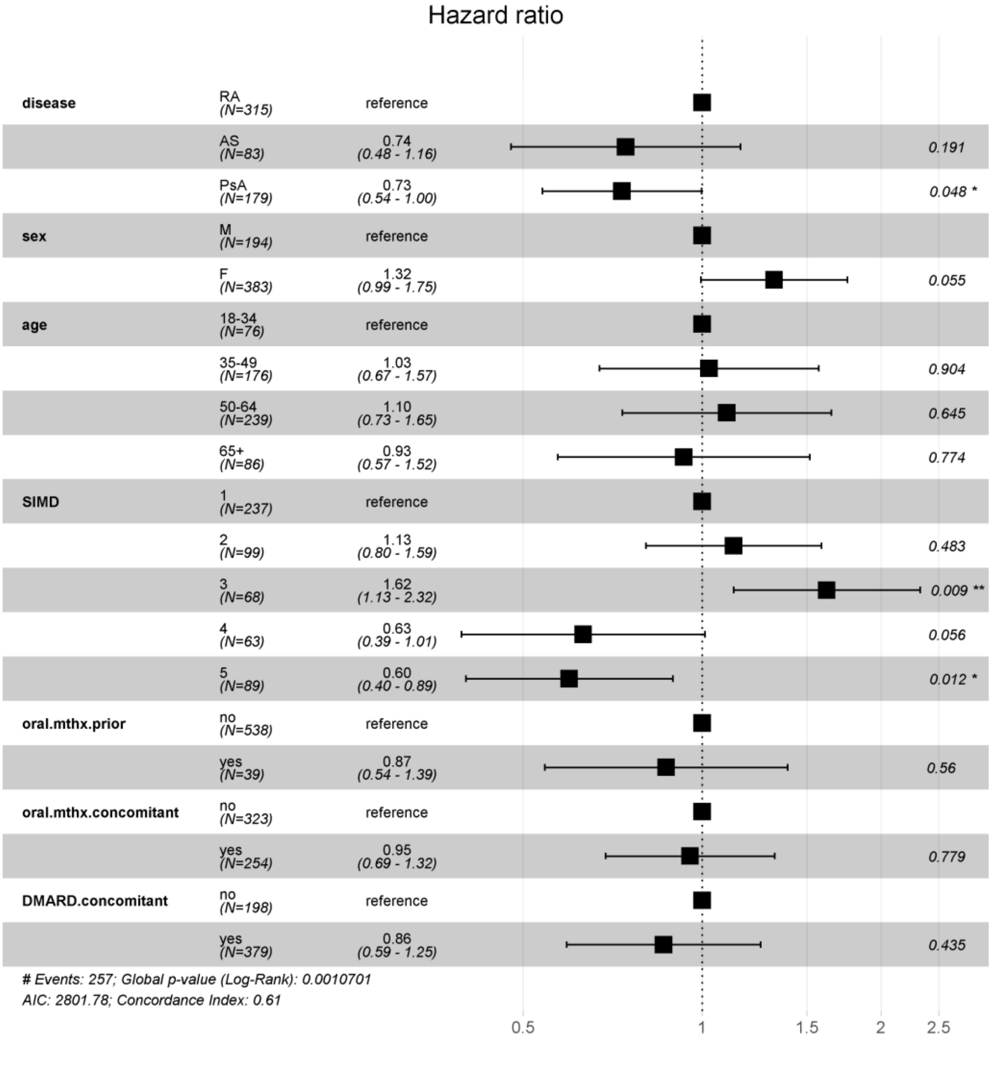

Adherence

Incident patients with at least two deliveries of the first (index) biologic (n = 598, 88.9%) were used to evaluate adherence. With overall adherence over 80% and both MRA and CR results showing the same patterns as the overall cohort.

Supplementary Figure 6: Adherence to biologics by rheumatic condition according to %MRA (5A) and %CR (5B). MRA- medication refill adherence; CR- compliance rate.

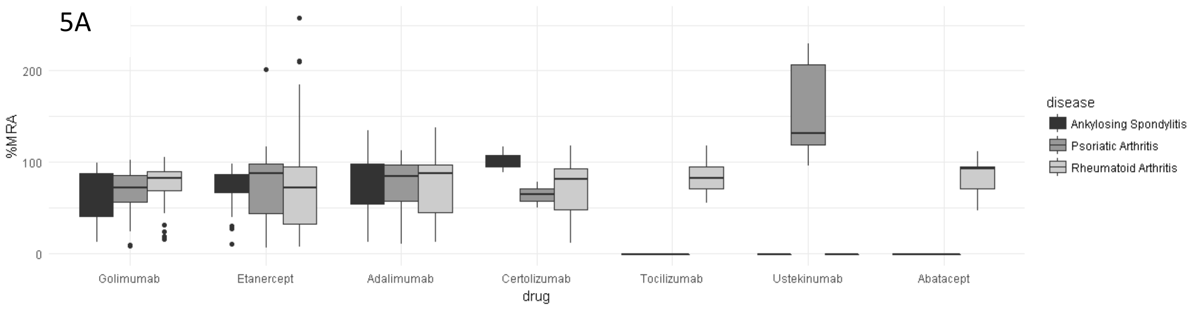

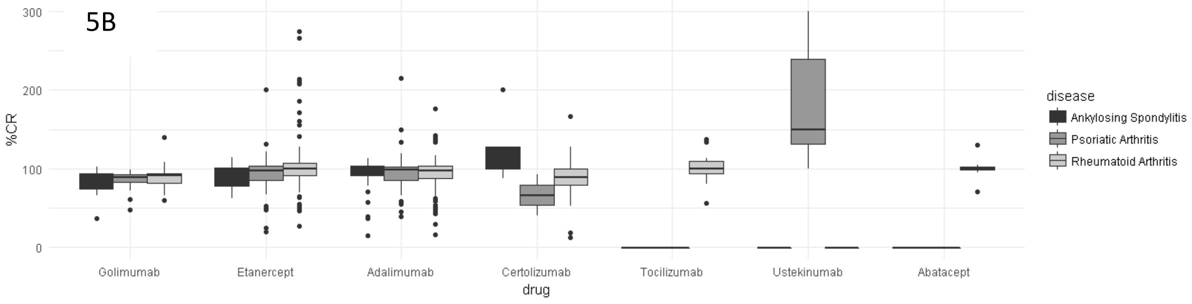

References

1. Widdifield J, Labrecque J, Lix L, et al. Systematic review and critical appraisal of validation studies to identify rheumatic diseases in health administrative databases. *Arthritis Care Res* 2013; **65**(9): 1490-503.
2. Herrett E, Thomas SL, Schoonen WM,et al. Validation and validity of diagnoses in the General Practice Research Database: a systematic review. *Br J Clin Pharmacol* 2010; **69**(1): 4-14.
3. Dubreuil M, Peloquin C, Zhang Y,et al. Validity of Ankylosing Spondylitis Diagnoses in The Health Improvement Network. *Pharmacoepidemiol Drug Saf* 2016; **25**(4): 399-404.
4. Thomas SL, Edwards CJ, Smeeth L, et al. How accurate are diagnoses for rheumatoid arthritis and juvenile idiopathic arthritis in the general practice research database? *Arthritis Rheum* 2008; **59**(9): 1314-21.
