# Supplementary material for: Discontinuation, persistence and adherence to subcutaneous biologics delivered via a homecare route to Scottish adults with rheumatic diseases: a retrospective study
Source: BMJ Open. 2019 Sep 4;9(9):e027059. doi: 10.1136/bmjopen-2018-027059 (PMC6731860; doi:10.1136/bmjopen-2018-027059)
Supplement: Author's manuscript [file bmjopen-2018-027059.draft_revisions.pdf]

BMJ Open is committed to open peer review. As part of this commitment we make the peer review history of every article we publish publicly available.

When an article is published we post the peer reviewers' comments and the authors' responses online. We also post the versions of the paper that were used during peer review. These are the versions that the peer review comments apply to.

The versions of the paper that follow are the versions that were submitted during the peer review process. They are not the versions of record or the final published versions. They should not be cited or distributed as the published version of this manuscript.

BMJ Open is an open access journal and the full, final, typeset and author-corrected version of record of the manuscript is available on our site with no access controls, subscription charges or pay-per-view fees (http://bmjopen.bmj.com).

If you have any questions on BMJ Open's open peer review process please email info.bmjopen@bmj.com

BMJ Open

Routine homecare data cohort can be used to assess subcutaneous biologic use: persistence, discontinuation and adherence

Journal:	*BMJ Open*
Manuscript ID	bmjopen-2018-027059
Article Type:	Research
Date Submitted by the Author:	03-Oct-2018
Complete List of Authors:	Alvarez-Madrazo, Samantha; University of Strathclyde, Health Data Research Scotland,Strathclyde Institute of Pharmacy and Biomedical Sciences<br>Kavanagh, Kimberly; University of Strathclyde Department of Mathematics and Statistics<br>Siebert, Stefan<br>Semple, Yvonne; University of Strathclyde, Strathclyde Institute of Pharmacy and Biomedical Sciences; Glasgow Royal Infirmary, Medicines Information, Pharmacy Department<br>Godman, Brian; Karolinska University Hospital, 5 Division of Clinical Pharmacology, Karolinska Institutet,<br>Maciel Almeida, Alessandra; Universidade Federal de Minas Gerais<br>Acurcio, Francisco ; Universidade Federal de Minas Gerais<br>Bennie, Marion; University of Strathclyde, Health Data Research Scotland, Strathclyde Institute of Pharmacy and Biomedical Sciences; NHS National Services Scotland, Information Services Division
Keywords:	biologics, psoriatic arthritis, rheumatoid arthritis, ankylosing spondylitis, persistence, adherence

SCHOLARONE™
Manuscripts

TITLE PAGE

Title: Routine homecare data cohort can be used to assess subcutaneous biologic use: persistence, discontinuation and adherence

Authors:

[1]Samantha Alvarez-Madrazo

[2]Kimberley Kavanagh

[3]Stefan Siebert

[1,4]Yvonne Semple

[1,5] Brian Godman

[6]Alessandra Maciel Almeida

[6]Francisco de Assis Acurcio

[1,7] Marion Bennie

Author affiliations:

[1]Health Data Research Scotland, Strathclyde Institute of Pharmacy and Biomedical Sciences, University of Strathclyde, Glasgow, UK
[2] Department of Mathematics and Statistics, University of Strathclyde, Glasgow,UK
[3] Institute of Infection Immunity and Inflammation, University of Glasgow,UK
[4] Medicines Information, Pharmacy Department, Glasgow Royal Infirmary,UK
[5] Division of Clinical Pharmacology, Karolinska Institutet, Karolinska University Hospital, Stockholm, Sweden
[6] Federal University of Minas Gerais, Brazil
[7] Information Services Division, NHS National Services Scotland, Edinburgh,UK

Corresponding author:

Prof. Marion Bennie

Strathclyde Institute of Pharmacy and Biomedical Sciences

University of Strathclyde

161 Cathedral Street

Glasgow

G4 0RE

Email: marion.bennie@strath.ac.uk

Tel. +44(0) 141 548 2113

Word count: 3988

For peer review only

For peer review only - http://bmjopen.bmj.com/site/about/guidelines.xhtml

ABSTRACT

Objectives: to understand patterns of subcutaneous (SC) biologic use over time in adults with inflammatory rheumatic musculoskeletal diseases receiving a homecare delivery service.

Design: retrospective cohort

Setting: patients in secondary care receiving SC biologics in the largest Scottish Health Board

Participants: a new bespoke cohort was created from routine data gathered as part of a health board Homecare Service Database. Patients over 18 years who received a supply of SC biologic from January 2012 - May 2015 with a diagnosis for rheumatoid arthritis (RA), psoriatic arthritis (PsA) or ankylosing spondylitis (AS) were included.

Outcomes measured: a standardised framework was applied by measuring discontinuation rates, persistence using Kaplan-Meier analysis and Cox regression and adherence using medication refill adherence (MRA) and Compliance Rate (CR).

Results: 751 patients were identified (AS – 105, PsA – 227, RA - 419) of whom 89.3% had more than one biologic delivery (median days' follow-up: AS - 494; PsA – 544; RA - 529) and 83.2% did not switch biologic. For all conditions, approximately half were persistent on their index biologic (52% AS, 54% PsA, 48%RA). Of patients who discontinued treatment, more re-initiated treatment (59% AS, 60% PsA and 47% RA), the majority with the same biologic, than ceased therapy. Overall adherence during the period of treatment was over 80% when calculated using MRA (median %MRA - AS 84.0%, PsA 85.0%, RA 82.4%) or CR (median %CR - AS 96.6%, PsA 97%, RA 96.6%).

Conclusion: Use of linked routine data is a sustainable pathway to enable on-going evaluation of biologics use. A more consistent approach to studying use (discontinuation, persistence and adherence metrics) should be adopted to enable comparability of studies.

Keywords: biologics, psoriatic arthritis, rheumatoid arthritis, ankylosing spondylitis,persistence, adherence

Article summary

Strengths and limitations of this study

- This is the first real-world cohort containing type, frequency and dose of seven subcutaneous biologics delivered directly to patients with ankylosing spondylitis, psoriatic arthritis and rheumatoid arthritis.
- The cohort is sustainable being generated as part of routine clinical care, can be easily updated and linked to other administrative datasets and provides a novel way to assess adherence, persistence and discontinuation of biologics.
- The limitations include having a mixed cohort of naive and existing biologic users, not capturing reasons for variablity in patients' response, use of intravenous biologics or prior use of any biologic or subcutaneous methotrexate.
- The generalisability of the results in a Scottish setting has to be tested, as this cohort covers the largest Scottish Health Board.

For peer review only - http://bmjopen.bmj.com/site/about/guidelines.xhtml

INTRODUCTION

Chronic inflammatory rheumatic musculoskeletal diseases such as rheumatoid arthritis (RA), psoriatic arthritis (PsA) and ankylosing spondylitis (AS) are characterised by inflammatory joint symptoms requiring long-term management. Over the past two decades, the use of biologic therapies has revolutionised their treatment, with national and global groups recommending their use in patients with moderate to severe disease unresponsive to conventional therapies.[1-5] Whilst clinical trials have demonstrated their short-term efficacy and safety, long-term evidence require observational studies and real-world data. Despite reassuring results to-date,[6-10] important challenges and unanswered questions remain.

Furthermore, despite the apparent shared inflammatory mechanisms and therapies, there are significant differences between these inflammatory conditions in terms of prevalence, gender and sex distribution, genetics, pathophysiology and clinical phenotype, including extra-articular manifestations and co-morbidities.[11-16] Therefore, the safety and long-term therapy data for one condition (usually RA due to the larger numbers and longer pharmacological history) cannot simply be assumed to apply to the other conditions, even for the same drug. It is therefore important to evaluate these factors in real-world settings across the range of conditions for which these drugs are used.

In addition to drug and disease-related factors, in clinical practice poor adherence and persistence to therapy are important detrimental factors, contributing to treatment failure and disease progression.[17] Most of the direct comparisons for adherence and persistence are from observational studies, mainly focusing on the first biologics to reach the market/clinical practice (infliximab, adalimumab and etanercept).[17-19] A few studies have incorporated other biologics,[20-26] with more biologics set to be approved in the upcoming years. Although the standardised measurement of adherence and persistence, dividing the process in three phases - initiation, implementation and discontinuation - has been advocated by Vrijens *et al.* and endorsed by the European Society for Patient Adherence, Compliance and Persistence (ESPACOMP),[27] this methodology has mainly been applied to oral medications and not to biologics, which are administered subcutaneously or intravenously, with complex dosing schedules.

Disease or drug registers are expensive, labour-intensive and not optimal for evaluating adherence and persistence, so other methods are required.[17, 18] In many countries, administrative or prescription datasets linked to reimbursement have been used,[28-31] but these do not always indicate whether the participant actually received the medication. Furthermore, biologics usually require specialist prescription so are not routinely captured in primary care prescription datasets.

This study aimed to use a homecare service database for subcutaneous biologics to understand the patterns of biologic use over time in a population of adults with rheumatic diseases receiving biologics.

For peer review only - http://bmjopen.bmj.com/site/about/guidelines.xhtml

METHODS

Data Sources

For this study a new bespoke, retrospective cohort of patients receiving subcutaneous biologics was created by linking administrative electronic health records (EHRs) at a patient level via an unique national (Scottish) health service identifier, known as the Community Health Index (CHI).[32] This dataset included two resources available in the largest Scottish Health Board, NHS Greater Glasgow and Clyde (GGC), with a population covering 1.2 million of the 5.4 million residents in Scotland.[33] The two novel resources were the Homecare Service Database (HSD) and the local Rheumatology Arthritis Database (RAD), in addition to three existing nationally held datasets: the Prescribing Information System (PIS),[34] the mortality records (National Records of Scotland-NRS) and the Scottish Morbidity Records (SMR01).[35] The variables obtained from each dataset are outlined in Figure 1.

Figure 1. Variables obtained from different Scottish datasets.

Of the GGC patients receiving biologics up until early 2018, 98% received subcutaneous biologics uniquely via the homecare delivery route, with delivery details captured in the HSD and managed through the pharmacy distribution system in the health board. Since 2012 the HSD has systematically captured information about patients (e.g. hospital attended, indication for biologics, drugs and dose, administration and delivery frequency, date and quantity delivered), prescribers and healthcare delivery companies. The remaining 2% of patients receiving intravenous biologics are not captured in the HSD as these medicines are administered directly in hospital day wards.

The RAD contains information of patients who have attended rheumatology clinics in two of the four main hospitals in Glasgow since 1997. Diverse clinical variables are recorded, including the main and secondary diagnoses, biologic screening/follow up, comorbidities, other medications and lifestyle factors.

The PIS includes prescriptions prescribed, dispensed and reimbursed within the community setting,[34] NRS captures cause-specific mortality records and SMR01 has episode-level data on all hospital inpatient and day care episodes from hospitals in Scotland.[35]

Study population

Patients who received at least one delivery of subcutaneous biologics in the HSD dataset with a diagnosis for AS, PsA or RA were eligible for this study. The RAD database was used to validate the diagnosis in a subset of HSD patients (see supplementary file for validation methods and results).

All patients who had a biologic supply date between January 2012 and May 2015 and were at least 18 years old at cohort entry were included. All patients were followed-up until death, migration to a primary care practice outside Scotland or end date of the study (May 2015), whichever occurred first. In the overall cohort, both prevalent and incident users were included (n=751, 105 AS, 227 PsA and 419 RA). To illustrate the stability of findings, a subset analysis was carried out with only incident users (n=673, 95 AS, 208 PsA and 370

For peer review only - http://bmjopen.bmj.com/site/about/guidelines.xhtml

RA), defined as patients who did not receive a subcutaneous biologic in the first six months of the study period (from January-June 2012) (online supplementary file).

Patients were excluded if their first biologic delivery was prior to the date of approval for the drug by the Scottish Medicines Consortium (SMC), which is the national health technology assessment process for all new medicines in Scotland,[36] or had missing records in the HSD for dose, frequency or how often the delivery of the biologics would take place.

The first delivery date for any biologic was established as the index date and the first biologic recorded was considered the index biologic. Age was calculated at index date. Socioeconomic status was assessed using the Scottish Index of Multiple Deprivation quintile (SIMD) 2012 quintiles, a measure that incorporates different aspects of deprivation into a single indexTo assess co-medication, patients with at least one prescription in the 12 months prior to the index date in PIS for antibacterials (British National Formulary- BNF section 5.1), antifungals (BNF section 5.2), antivirals (BNF section 5.3), oral methotrexate (prescribed item methotrexate), Disease-Modifying Anti-Rheumatic Drugs (DMARDs - BNF subsection 10.1.3), Non-Steroidal Anti-inflammatory Drugs (NSAIDs - BNF subsection 10.1.1) and corticosteroids (BNF subsections 1.5.2 or 6.3.2) were recorded.

Data analyses

In this study, we advocate the use of a standardised framework that includes measurements of discontinuation, persistence and adherence.[27, 37]

Considering the variation in the administration frequencies and number of injections delivered according to biologic, first the estimated days covered per delivery was calculated by multiplying the quantity delivered by the standard administration frequency according to the approved licence for each biologic. Knowing the delivery date and the estimated days covered allowed estimation of the expected end date covered and how it related with the following delivery, and whether there were gaps or not. These metrics were the basis of the utilisation analysis.

Discontinuation was defined as the end of treatment, either because there was a considerable gap of more than 56 days between deliveries (re-initiation) or no more deliveries were received (cessation). Those who did not discontinue were defined as persistent. Discontinuation rates were estimated using the refill-gap method.[38] Patients were censored at first discontinuation, pre-defined as a gap of more than 56 days with no biologics delivery after exhaustion of the days' supplied by the previous delivery, regardless of whether or not patients switched to another biologic. This gap was selected because most biologics were delivered bimonthly, approximately 5 times the half-life for the biologics of interest would have elapsed, and this was comparable to other studies.[26, 29] A sensitivity analysis was conducted using a gap of 28 days (minimum period between deliveries) and 84 days (maximum period between deliveries) (see supplementary file online).

Crude persistence was estimated using Kaplan-Meier analysis and Cox regression were used to assess persistence post-index date, in patients with at least 6 months follow-up.The hazard ratios (HRs) for persistence with biologics were adjusted for sex, age, SIMD, use of index biologic or not (meaning a switch could have happened whilst being persistent), use of oral methotrexate a year prior to first delivery of the biologic, and concomitant use of oral methotrexate or DMARD during study period.

For peer review only - http://bmjopen.bmj.com/site/about/guidelines.xhtml

Adherence is defined as to what extent the biologics delivered correspond to the treatment regimen recommended in the BNF.There is no consensus in the literature on how best to estimate adherence thus two measurements were used for patients with at least two deliveries of the index biologic: medication refill adherence (MRA) and compliance rate (CR).[37, 39] MRA gives an overall adherence percentage describing the exposure during the whole study period regardless of the time when the last prescription was delivered and is calculated as (total days' supply/total days in study) x 100. In contrast, CR describes the exposure between first and last prescription delivered (total days' supply-last delivery/days first up to but not including last delivery) x 100.

Analyses were performed in R software, version 3.3.0.

Patient and public involvement

The prelimary design of this study was presented to the Farr Scotland Public Panel. According to their experience, and preferences the research questions and outcome measures were developed to make the results generalisable to patients with rheumatic conditions.

RESULTS

The study included 751 patients: 105 with AS, 227 with PsA and 419 with RA (Table 1). From them 673 patients (89.6%) were incident users, 95 AS (90.5%), 208 PsA (91.6%) and 370 RA (88.3%).The patients with RA were older and had a higher proportion of females. Regardless of diagnosis, the majority of patients receiving biologics resided in the most deprived areas.

From the overall cohort, a total of 89.3% of patients had more than one delivery of biologic during the study period and 83.2% did not switch biologic. Patients were most commonly started on adalimumab or etanercept as index biologic. The median days' follow-up on biologic therapy, regardless of whether or not the patients switched to others biologics, was: 494 days in AS; 544 days in PsA and 529 days in RA patients.

Use of concomitant non-biologic medication during the study period was more frequent amongst patients with RA with oral methotrexate (54.9%), any DMARDs (81.6%) and NSAIDs (63.2%) most commonly prescribed. A similar pattern of medication use was observed in PsA patients. In contrast, in AS patients, NSAIDs (71.4%) were the most commonly co-prescribed medications. Prescription of at least one course of antibiotics in patients during the study period was similar for the three conditions (RA – 62.5%, PsA – 59.9%, AS – 49.5%). Similar trends of concomitant medications were found in the incident cohort (see supplementary file).

For peer review only - http://bmjopen.bmj.com/site/about/guidelines.xhtml

For peer review only

Table 1. Baseline characteristics of patients with rheumatic conditions receiving subcutaneous biologic from Jan 2012- May 2015 (n=751)

Rheumatic disease	All		Ankylosing spondylitis		Psoriatic arthritis		Rheumatoid arthritis	
n	**751**		**105**		**227**		**419**	
**Female - n (%)**	**497**	(66.2)	**35**	(33.3)	**130**	(56.5)	**332**	(78.7)
**Age***								
Age (median, IQR)*	**53.0(42.7-60.9)**		**47.2 (35.4-55.0)**		**48.0(40.2-57.3)**		**56.0(47.4-64.2)**	
**18-34 (%)**	**93**	(12.4)	**24**	(22.9)	**36**	(15.9)	**33**	(7.9)
**35-49 (%)**	**222**	(29.6)	**36**	(34.3)	**91**	(40.1)	**95**	(22.7)
**50-64 (%)**	**313**	(41.7)	**36**	(34.3)	**82**	(36.1)	**195**	(46.5)
**65+ (%)**	**123**	(16.4)	**9**	(8.6)	**18**	(7.9)	**96**	(22.9)
SIMD*								
**1 Most deprived (%)**	**305**	(40.6)	**42**	(42.9)	**93**	(42.1)	**170**	(41.5)
**2 (%)**	**132**	(17.6)	**21**	(21.4)	**44**	(19.9)	**67**	(16.3)
**3 (%)**	**81**	(10.8)	**13**	(13.3)	**23**	(10.4)	**45**	(11)
**4 (%)**	**93**	(12.4)	**15**	(15.3)	**25**	(11.3)	**53**	(12.9)
**5 Least deprived (%)**	**118**	(15.7)	**7**	(7.1)	**36**	(16.3)	**75**	(18.3)
Number of deliveries of biologics								
1	**80**	(10.7)	**14**	(13.3)	**19**	(8.4)	**47**	(11.2)
2-10	**456**	(60.7)	**59**	(56.2)	**141**	(62.1)	**256**	(61.1)
11-20	**156**	(20.8)	**25**	(23.8)	**49**	(21.6)	**82**	(19.6)
21+	**59**	(7.8)	**7**	(6.7)	**18**	(7.9)	**34**	(8.1)

For peer review only - http://bmjopen.bmj.com/site/about/guidelines.xhtml

Number of different biologics delivered								
1	**625**	(83.2)	**89**	(84.8)	**179**	(78.9)	**357**	(85.2)
2, 3 or 4 (switchers)	**126**	(16.8)	**16**	(15.2)	**48**	(21.1)	**62**	(14.8)
First biologic delivered								
**Abatacept**	**8**	(1.1)	**NA**		**NA**		**8**	(1.9)
**Adalimumab**	**305**	(40.6)	**55**	(52.4)	**112**	(49.3)	**138**	(32.9)
**Certolizumab Pegol**	**77**	(10.3)	**6**	(5.7)	**<5**		**69**	(16.5)
**Etanercept**	**240**	(32)	**24**	(22.9)	**82**	(36.1)	**134**	(32)
**Golimumab**	**93**	(12.4)	**20**	(19)	**24**	(10.6)	**49**	(11.7)
**Tocilizumab**	**21**	(2.8)	**NA**		**NA**		**21**	(5)
**Ustekinumab**	**7**	(0.9)	**NA**		**7**	(3.1)	**NA**	
**Follow-up (days), median (range)**	**529**	**(284.0-852.0)**	**494 (285-838)**		**544 (280-831)**		**529 (283.5-866.5)**	
Concomitant medication use 1 year prior first delivery								
**Antibacterial use**	**48**	(6.4)	**6**	(5.7)	**11**	(4.8)	**31**	(7.4)
**Antifungal use**	**<10**		**NA**		**<5**		**6**	(1.4
**Antiviral use**	**<5**		**NA**		**NA**		**<5**	
**Oral methotrexate use**	**89**	(11.9)	**<5**		**23**	(10.1)	**63**	(15)
**Subcutaneous methotrexate use**	**NA**		**NA**		**NA**		**NA**	
**Any DMARD**	**115**	(15.3)	**<5**		**28**	(12.3)	**84**	(20)
**NSAID**	**114**	(15.2)	**17**	(16.2)	**28**	(12.3)	**69**	(16.5)
**Gluco or corticosteroids**	**19**	(2.5)	**<5**		**<5**		**16**	(3.8)

For peer review only - http://bmjopen.bmj.com/site/about/guidelines.xhtml

Concomitant medication use during study period								
**Antibacterial use**	**450**	(59.9)	**52**	(49.5)	**136**	(59.9)	**262**	(62.5)
**Antifungal use**	**72**	(9.6)	**9**	(8.6)	**26**	(11.5)	**37**	(8.8)
**Antiviral use**	**53**	(7.1)	**<5**		**17**	(7.5)	**34**	(8.1)
**Oral methotrexate use**	**332**	(44.2)	**10**	(9.5)	**92**	(40.5)	**230**	(54.9)
**Subcutaneous methotrexate use**	**14**	(1.9)	**NA**		**<5**		**11**	(2.6)
**Any DMARD**	**493**	(65.6)	**12**	(11.4)	**139**	(61.2)	**342**	(81.6)
**NSAID**	**491**	(65.4)	**75**	(71.4)	**151**	(66.5)	**265**	(63.2)
**Gluco or corticosteroids**	**148**	(19.7)	**10**	(9.5)	**29**	(12.8)	**109**	(26.0)
**Comorbidities**								
Charlson score								
0	**163**	(21.7)	**38**	(36.2)	**75**	(33)	**50**	(11.9)
1	**190**	(25.3)	**8**	(7.6)	**15**	(6.6)	**167**	(39.9)
2+	**34**	(4.5)	**<5**		**5**	(2.2)	**28**	(6.7)
unknown	**364**	(48.5)	**58**	(55.2)	**132**	(58.1)	**174**	(41.5)

*Age at first delivery, ** from 751 patients 22 did not have Scottish Index of Multiple Deprivation (SIMD) values. Follow-up is from first delivery to study end date. DMARD- disease-modifying anti-rheumatic drugs; NSAID- non-steroidal anti-inflammatory drug

For peer review only - http://bmjopen.bmj.com/site/about/guidelines.xhtml

Discontinuation and persistence

For all three conditions, approximately half of the patients were persistent on their index biologic throughout the study period (52% AS, 54% PsA, 48%RA). This was also observed in the incident cohort (55% AS, 55% PsA, 49%RA) as shown in Supplementary Figure 2

A small proportion of patients were continuously (no period of discontinuation) on biologics throughout the study period (persistent) but switched from their index to another biologic (overall cohort 4% AS, 9% PsA and 5% RA; incident cohort 4% AS, 9% PsA and 6% RA) (Figure 2 overall cohort and Supplementary Figure 2 incidence cohort). The remaining patients had a discontinuation, with a gap longer than 56 days between deliveries. Of these, in the overall cohort 41% of AS, 40% of PsA and 53% of RA patients ceased treatment (did not restart a subcutaneous biologic during the study period) (incident cohort 43% AS,41%PsA, 55%RA).

More patients re-initiated a biologic after a period of discontinuation (overall cohort 59% AS, 60% PsA and 47% RA;incident cohort 57% AS, 59% PsA and 45% RA,respectively), of which the majority (overall cohort 44% AS, 49% PsA and 45% RA; incident cohort 44%AS, 51% PsA, 43%RA) re-initiated the same biologic received at the index date and only a small number switched to an alternate subcutaneous biologic (overall cohort 15% AS, 11% PsA and 2% RA; incident cohort 13%AS, 8% PsA, 2%RA). A sensitivity analysis was conducted using a gap of 28 and 84 days showing similar trends (results for overall and incident cohorts are in supplementary file online).

Figure 2. Persistence and discontinuation (re-initiating or ceasing) of treatment with biologics according to rheumatic condition (n=751)

The crude survival curve of *time to discontinuation* (first gap greater than 56 days between biologic deliveries) of any biologic is shown in Figure 3. In the crude model, the proportion of patients persisting is slightly higher in PsA and AS compared to RA; however, there is no significant difference according to the log-rank test (p=0.0983). The same results were observed in the incident cohort (p=0.165; Supplementary Figure 3).

Figure 3:Crude survival curves comparing persistent patients according to rheumatic condition. (n=655)

After adjustment for covariates, there was a slight difference between RA and PsA patients (HR =0.73, 95%CI:0.55-0.97, p=0.03). Lower risk of discontinuation was associated with the two least deprived quintiles (SIMD 4 or 5), persisting after switching biologic (vs index biologic) and use of oral methotrexate prior the study period, whereas female sex and SIMD 3 (deprived quintile) were associated with a higher risk of discontinuation as shown in Figure

For peer review only - http://bmjopen.bmj.com/site/about/guidelines.xhtml

4. Age, SIMD 2 (more deprived quintile), concomitant use of oral methotrexate or DMARD were not statistically signficant covariates and did not influence the model, which excluded 21 patients with missing SIMD values. A subanalysis was performed with the incident cohort in which there was no longer a difference between rheumatic conditions and use of methotrexate or DMARD prior or during the study, however, the results of sex, SIMD quintiles and persistence after switching biologic remained significant (see Supplementary Figure 4).

Figure 4: Adjusted Hazard Ratios for persistence with biologics using Cox regression analysis excluding patients without SIMD values (n=634)

Adherence

Patients with at least two deliveries of the first (index) biologic (n = 671, 89.3%) were used to evaluate adherence, calculated using two methods.

Overall adherence during the period of treatment was over 80% when calculated using either the MRA or CR. However, this was consistently higher using %CR (median(IQR) for AS 96.6% (86.6-103.5), PsA 97% (85.5-103.2) and RA 96.6% (85.9-102.7) than using %MRA (median(IQR) for AS 84.0% (54.73-94.74), PsA 85.0% (50.76-98.25) and RA 82.4% (47.24-95.77)). The same pattern was also observed when comparing these two methods by the first biologic received (Figure 5A and 5B). Regardless of which of the two measurements were used, the few PsA patients (n=7) taking ustekinumab had higher adherence than was seen with any other biologic. Similar adherence results were obtained in the subset with incident users (Supplementary Figure 5).

Figure 5: Adherence to biologics by rheumatic condition according to %MRA (5A) and %CR (5B). MRA- medication refill adherence; CR- compliance rate.

DISCUSSION

This study is the first real-world cohort using data about SC biologic deliveries, combined with routine data from rheumatology clinics, community pharmacy dispensing, hospital discharges and mortality records to assess the utilisation of biologics in patients with RA, AS and PsA over a period of three years. This study found for all three conditions, approximately 50% of the patients were persistent on their index biologic and that overall adherence during the period of treatment was over 80%. Almost half of patients who discontinued their index biologic for a period greater than 56 days, ultimately re-initiate the same therapy with only a minority switching to another biologic. This highlights the importance of longitudinal data as many of these patients would have been incorrectly counted as cessations in other datasets.

For peer review only - http://bmjopen.bmj.com/site/about/guidelines.xhtml

A diverse range of studies have reported on the use of biologics over the last 20 years with the majority focused solely on RA,[19, 29, 40] and few studies comparing utilisation across the three main inflammatory rheumatologic conditions within a single healthcare system. Patient demographic characteristics in our study are similar to those reported in other epidemiological studies in the UK, Denmark, Norway and Germany,[11-16] despite the different methodologies used to create these cohorts.

Linked prescription and administrative health datasets have been used to study the utilisation of biologics in real-world settings in other studies in Europe, USA, Canada and Brazil. [24, 28, 29, 41, 42] However, to the best of our knowledge, this is the first study to use routine data generated through “direct to home” deliveries of biologics as a source of information to assess how patients use these medicines. Most other routine administrative healthcare studies use prescriptions or claims/reimbursement data. In contrast, this homecare delivery service requires active participation of the patient in both ordering and then signing for receipt of the biologic, thereby confirming the biologic was prescribed, the prescription was processed and the patient received the biologic, providing better insight into how patients are using their medicines.

To understand the use of medicines in our cohort, we adopted the taxonomy proposed by ESPACOMP.[27] In summary, the framework enables us to quantify a patient’s medicine taking behaviour in three respects: intensity, continuity and duration - how much of a drug does a patient take, how frequently, and for how long? *Persistence* and *discontinuation* allow us to study continuity and duration of treatment; while *adherence* enables us to study intensity of treatment in the context of the prescribed treatment regimen.

In those studies which examined persistence and discontinuation with biologics across rheumatic conditions, using a consistent in-study method over a comparable time period to our study, persistence was reported as being lower in RA than AS.[42, 43] This is similar to our findings where patients with RA were most likely to discontinue their index subcutaneous biologic, potentially reflecting the treat-to-target approach and greater therapeutic options available for RA at the time of this study. Our study also enabled quantification and categorisation of biologic switching, we believe for the first time, in both patients who were persistent regardless of changes in therapy and those who discontinued index therapy and then commenced a new biologic (Figure1).

In our Cox regression analyses, female sex was associated with lower persistence being consistent with several studies.[40, 42, 43] However, our findings regarding socieconomic status contradict those of the Machado *et al.* study as we have shown higher persistence in patients with lower deprivation levels. There are also discrepancies in the association with the use of methotrexate, as Heiberg *et al.* reported a strong association of its concomitant use with persistence in the three rheumatic diseases and we found an association with prior use. Randomised controlled trials of biologics in patients with PsA have failed to demonstrate superiority of combination therapies.[44, 45] Thus the role of methotrexate as a predictor for persistence remains unclear.

Persistence after the first, second or subsequent courses of biologics is still controversial. A French study found better persistence with first biologic compared to second or third,[46] with similar tendencies reported in Norway by Heiberg *et al.* In contrast, in a large UK national cohort of RA patients, those who switched to a second biologic had higher persistence[47] and

For peer review only - http://bmjopen.bmj.com/site/about/guidelines.xhtml

the study using the RADIUS registry found similar persistence between first and second biologic.[48] In our study instead of comparing each course, we compared those persisting with their index biologic (first biologic) with those persisting after switching. Our findings were similar to the UK cohort as persistence was higher in those who had switched.

Various studies have examined adherence in a single rheumatoid condition, often RA[18-24, 26-29] but we could only find a single Italian study measuring adherence across RA, AS and PsA,[41] though applying a different method to our study. The challenge in comparing results between studies is often the absence of any detail on how adherence was measured and where specified, multiple measures being used. This has been highlighted in a recent systematic review and meta-analysis by Scheiman-Elazary *et al*. of anti-arthritis medication in RA which reported adherence ranging from 10.5%-98.5% with the meta-analysis generating an adherence rate of 66% (95%CI 58-75%), noting that variability may result from different methods and readers should appraise studies according to the validity of the method, the scales and cut-off points.[18] From our experience of using both MRA and CR for adherence measurement we would recommend the use of CR as it is simple to calculate and includes the period between deliveries, thereby removing any ambiguity arising following the last supply of medicine.

In this study, there was higher adherence to ustekinumab compared to the other biologics which may relate in part to the small numbers and more recent introduction and therefore shorter use of this agent at the time of the study, although this observation has also been reported in psoriasis cohorts.[49, 50]

This current study has several strengths. This is the first real-world cohort containing type, frequency and dose of subcutaneous biologics delivered directly to patients for these three rheumatic diseases. The cohort is sustainable as it is generated as part of routine clinical care, avoiding the need for additional data-collection burden required in a traditional registry model, it can be easily updated and linked to other administrative datasets, has better coverage than individually-held clinical data and allows the use of biologics to be studied over time. The sustainability is a key factor, enabling biosimilar and new future innovator biologics to be easily captured as these reach the clinic. This model can also be easily extended to other specialities, including dermatology and gastroenterology, where similar biologic therapies are used. Recording the confirmed delivery of drug directly to patients may reduce some of the uncertainty and limitations associated with using prescription or reimbursement administrative datasets where it cannot be determined if patients actually received the drug. Recall bias was minimized by linking routine data and not based on self-reported persistence or adherence, while selection bias was addressed by including all the patients receiving biologics in the largest Scottish Health Board.

This study has also some limitations. The cohort covers a specific, albeit highly populated, region in Scotland and the generalisability of the results has to be tested. It is a mixed cohort of naive and existing biologic users, which could influence the assessment of medication utilisation. However, the results were similar in the overall and incident cohorts. The reason for variability in patients' response is not currently captured, so it is of limited utility in terms of understanding the factors that influence adherence. History of biologics or subcutaneous methotrexate being used prior to 2012 was not available. However, during the study period the use subcutaneous methotrexate was captured within the HSD, recording a relatively low use. The biologics home delivery service only captures subcutaneous biologics and not

For peer review only - http://bmjopen.bmj.com/site/about/guidelines.xhtml

intravenous agents, such as infliximab or rituximab, so will have missed where patients were switched to these agents. However, the latter are far smaller in volume and may be captured from rheumatology clinic documentation and hospital administrative data where attendance for infusion guarantees receipt of drug.

CONCLUSION

The long-term evaluation of biologics for the treatment of rheumatic and related diseases is a desirable goal on a clinical level, but it is also a requirement from a pharmacological and economic point of view. Traditional drug or disease registers are expensive, resource intensive and difficult to sustain, so strategies using linked administrative and routine clinical data provide the best opportunity to capture the true picture of biologic use. Additionally a homecare delivery database may reduce some of the uncertainties associated with other administrative databases.

In moving forward, we strongly advocate that future studies should consider application of the ESPACOMP framework[25] to better understand the use of biologics i.e. the combination of discontinuation, persistence and adherence metrics thus leading to a more consistent approach which enables more comparability between studies.

Acknowledgements: Farr Institute @ Scotland, GGC Safe Haven team, Norman Lannigan and Kevin Milne for providing the Homecare Database and allow its use for research purposes. FAA acknowledges the support from FAPEMIG, the Minas Gerais State Research Foundation, Brazil.

REFERENCES

1. Coates LC, Kavanaugh A, Mease PJ, et al. Group for Research and Assessment of Psoriasis and Psoriatic Arthritis 2015 Treatment Recommendations for Psoriatic Arthritis. *Arthritis Rheumatol (Hoboken, NJ)* 2016; **68**(5): 1060-71.
2. Gossec L, Smolen JS. European League Against Rheumatism (EULAR) recommendations for the management of psoriatic arthritis with pharmacological therapies: 2015 update. *Ann Rheum Dis* 2016; **75**(3): 499-510.
3. NICE. Rheumatoid arthritis in adults: management. Clinical guideline CG79. England: National Institute for Health and Care Excellence; 2015.
4. Smolen JS, Landewe R, Bijlsma J, et al. EULAR recommendations for the management of rheumatoid arthritis with synthetic and biological disease-modifying antirheumatic drugs: 2016 update. *Ann Rheum Dis* 2017; **76**(6): 960-77.
5. van der Heijde D, Ramiro S. 2016 update of the ASAS-EULAR management recommendations for axial spondyloarthritis. *Ann Rheum Dis* 2017; **76**(6): 978-91.
6. Macfarlane GJ, Barnish MS, Jones EA, et al. The British Society for Rheumatology Biologics Registers in Ankylosing Spondylitis (BSRBR-AS) study: Protocol for a prospective cohort study of the long-term safety and quality of life outcomes of biologic treatment. *BMC Musculoskelet Disord* 2015; **16**: 347.
7. Nam JL, Takase-Minegishi K, Ramiro S. Efficacy of biological disease-modifying antirheumatic drugs: a systematic literature review informing the 2016 update of the EULAR recommendations for the management of rheumatoid arthritis. *Ann Rheum Dis* 2017; **76**(6): 1113-36.
8. Nikiphorou E, Buch MH, Hyrich KL. Biologics registers in RA: methodological aspects, current role and future applications. *Nat Rev Rheumatol* 2017; **13**(8): 503-10.

For peer review only - http://bmjopen.bmj.com/site/about/guidelines.xhtml

9. Saad AA, Ashcroft DM, Watson KD,et al. Efficacy and safety of anti-TNF therapies in psoriatic arthritis: an observational study from the British Society for Rheumatology Biologics Register. *Rheumatology (Oxford, England)* 2010; **49**(4): 697-705.
10. Simard JF, Arkema EV, Sundstrom A, et al. Ten years with biologics: to whom do data on effectiveness and safety apply? *Rheumatology (Oxford, England)* 2011; **50**(1): 204-13.
11. Dean LE, Jones GT, MacDonald AG, et al. Global prevalence of ankylosing spondylitis. *Rheumatology* 2014; **53**(4): 650-7.
12. Egeberg A, Kristensen LE, Thyssen JP, et al. Incidence and prevalence of psoriatic arthritis in Denmark: a nationwide register linkage study. *Ann Rheum Dis* 2017; **8**(210963): 2016-210963.
13. Hoff M, Gulati AM, Romundstad PR,et al. Prevalence and incidence rates of psoriatic arthritis in central Norway: data from the Nord-Trondelag health study (HUNT). *Ann Rheum Dis* 2015; **74**(1): 60-4.
14. Humphreys JH, Verstappen SM, Hyrich KL, et al. The incidence of rheumatoid arthritis in the UK: comparisons using the 2010 ACR/EULAR classification criteria and the 1987 ACR classification criteria. Results from the Norfolk Arthritis Register. *Ann Rheum Dis* 2013; **72**(8): 1315-20.
15. Huscher D, Thiele K, Rudwaleit M, et al. Trends in treatment and outcomes of ankylosing spondylitis in outpatient rheumatological care in Germany between 2000 and 2012. *RMD Open* 2015; **1**(1): 2014-000033.
16. Ogdie A, Weiss P. The Epidemiology of Psoriatic Arthritis. *Rheum Dis Clin North Am* 2015; **41**(4): 545-68.
17. Blum MA, Koo D, Doshi JA. Measurement and rates of persistence with and adherence to biologics for rheumatoid arthritis: a systematic review. *Clin Ther* 2011; **33**(7): 901-13.
18. Scheiman-Elazary A, Duan L, Shourt C, et al. The Rate of Adherence to Antiarthritis Medications and Associated Factors among Patients with Rheumatoid Arthritis: A Systematic Literature Review and Metaanalysis. *J Rheumatol* 2016; **43**(3): 512-23.
19. Vangeli E, Bakhshi S, Baker A, et al. A Systematic Review of Factors Associated with Non-Adherence to Treatment for Immune-Mediated Inflammatory Diseases. *Adv Ther* 2015; **32**(11): 983-1028.
20. Bhoi P, Bessette L, Bell MJ,et al. Adherence and dosing interval of subcutaneous antitumour necrosis factor biologics among patients with inflammatory arthritis: analysis from a Canadian administrative database. *BMJ open* 2017; **7**(9): e015872.
21. Ebina K, Hashimoto M, Yamamoto W, et al. Drug retention and discontinuation reasons between seven biologics in patients with rheumatoid arthritis -The ANSWER cohort study. *PloS one* 2018; **13**(3): e0194130.
22. Glintborg B, Ostergaard M, Krogh NS, et al. Clinical response, drug survival and predictors thereof in 432 ankylosing spondylitis patients after switching tumour necrosis factor alpha inhibitor therapy: results from the Danish nationwide DANBIO registry. *Ann Rheum Dis* 2013; **72**(7): 1149-55.
23. Kruger K, Burmester GR, Wassenberg S,et al. Effectiveness and safety of golimumab in patients with rheumatoid arthritis, psoriatic arthritis and ankylosing spondylitis under real-life clinical conditions: non-interventional GO-NICE study in Germany. *BMJ open* 2018; **8**(6): e021082.
24. Lyu R, Govoni M, Ding Q, et al. Treatment persistence among patients with rheumatoid disease (RA, AS, PsA) treated with subcutaneous biologics in Germany. *Rheumatol Int* 2015; **28**: 28.
25. Oei HB, Hooker RS, Cipher DJ, Reimold A. High rates of stopping or switching biological medications in veterans with rheumatoid arthritis. *Clin Exp Rheumatol* 2009; **27**(6): 926-34.
26. Wallman JK, Kapetanovic MC, Petersson IF,et al. Comparison of non-radiographic axial spondyloarthritis and ankylosing spondylitis patients--baseline characteristics, treatment adherence, and development of clinical variables during three years of anti-TNF therapy in clinical practice. *Arthritis Res Ther* 2015; **17**(378): 015-0897.
27. Vrijens B, De Geest S, Hughes DA, et al. A new taxonomy for describing and defining adherence to medications. *Br J Clin Pharmacol* 2012; **73**(5): 691-705.

For peer review only - http://bmjopen.bmj.com/site/about/guidelines.xhtml

28. Acurcio FA, Machado MA, Moura CS, et al. Medication Persistence of Disease-Modifying Antirheumatic Drugs and Anti-Tumor Necrosis Factor Agents in a Cohort of Patients With Rheumatoid Arthritis in Brazil. *Arthritis Care Res* 2016; **68**(10): 1489-96.
29. Fisher A, Bassett K, Wright JM,et al. Comparative persistence of the TNF antagonists in rheumatoid arthritis--a population-based cohort study. *PloS one* 2014; **9**(8): e105193.
30. Ogale S, Hitraya E, Henk HJ. Patterns of biologic agent utilization among patients with rheumatoid arthritis: a retrospective cohort study. *BMC Musculoskelet Disord* 2011; **12**: 204.
31. Wadstrom H, Eriksson JK, Neovius M, et al. How good is the coverage and how accurate are exposure data in the Swedish Biologics Register (ARTIS)? *Scand J Rheumatol* 2015; **44**(1): 22-8.
32. ISD Scotland. ISD Scotland data dictionary. Edinburgh; 2016. http://www.ndc.scot.nhs.uk/Dictionary-A-Z/Definitions/index.asp?Search=C&ID=128&Title=CHI%20Number (accessed 10 December 2017).
33. NRS. Mid-year population estimates. Edinburgh; 2016. http://www. nrscotland.gov.uk/statistics-and-data/statistics/statistics-by-theme/ population/population-estimates/mid-year-population-estimates (accessed 10 December 2017)
34. Alvarez-Madrazo S, McTaggart S, Nangle C,et al. Data Resource Profile: The Scottish National Prescribing Information System (PIS). *Int J Epidemiol* 2016; **45**(3): 714-5f.
35. ISD Scotland. National Data Catalogue: National Datasets. Edinburgh; 2017. http://www.ndc.scot.nhs.uk/National-Datasets/. Accessed 10 December 2017.
36. Scottish Medicines Consortium . https://www.scottishmedicines.org.uk/. Accessed 22 June 2017.
37. Mueller T, Alvarez-Madrazo S, Robertson C, et al. Use of direct oral anticoagulants in patients with atrial fibrillation in Scotland: Applying a coherent framework to drug utilisation studies. *Pharmacoepidemiol Drug Saf* 2017; **26**(11): 1378-86.
38. Gregoire JP, Moisan J. Assessment of adherence to drug treatment in database research. In: Elseviers M, Wettermark B, Almarsdottir AB, et al., eds. Drug Utilization Research: Methods and Applications. Chichester: Wiley Blackwell; 2016: 369-80.
39. Hess LM, Raebel MA, Conner DA, et al. Measurement of adherence in pharmacy administrative databases: a proposal for standard definitions and preferred measures. *Ann Pharmacother* 2006; **40**(7-8): 1280-88.
40. Souto A, Maneiro JR, Gomez-Reino JJ. Rate of discontinuation and drug survival of biologic therapies in rheumatoid arthritis: a systematic review and meta-analysis of drug registries and health care databases. *Rheumatology (Oxford, England)* 2016; **55**(3): 523-34.
41. Degli Esposti L, Sangiorgi D, Perrone V, et al. Adherence and resource use among patients treated with biologic drugs: findings from BEETLE study. *Clinicoecon Outcomes Res* 2014; **6**: 401-7.
42. Machado MA, Moura CS, Ferre F, Bernatsky S, Rahme E, Acurcio Fde A. Treatment persistence in patients with rheumatoid arthritis and ankylosing spondylitis. *Rev Saude Publica* 2016; **50**(50): 2016050006265.
43. Heiberg MS, Koldingsnes W, Mikkelsen K, et al. The comparative one-year performance of anti-tumor necrosis factor alpha drugs in patients with rheumatoid arthritis, psoriatic arthritis, and ankylosing spondylitis: results from a longitudinal, observational, multicenter study. *Arthritis Rheum* 2008; **59**(2): 234-40.
44. Mease PJ, Gladman DD, Ritchlin CT, et al. Adalimumab for the treatment of patients with moderately to severely active psoriatic arthritis: results of a double-blind, randomized, placebo-controlled trial. *Arthritis Rheum* 2005; **52**(10): 3279-89.
45. Mease PJ, Kivitz AJ, Burch FX, et al. Etanercept treatment of psoriatic arthritis: safety, efficacy, and effect on disease progression. *Arthritis Rheum* 2004; **50**(7): 2264-72.
46. Duclos M, Gossec L, Ruyssen-Witrand A, et al. Retention rates of tumor necrosis factor blockers in daily practice in 770 rheumatic patients. *J Rheumatol* 2006; **33**(12): 2433-8.

For peer review only - http://bmjopen.bmj.com/site/about/guidelines.xhtml

47. Hyrich KL, Lunt M, Watson KD,et al. Outcomes after switching from one anti-tumor necrosis factor alpha agent to a second anti-tumor necrosis factor alpha agent in patients with rheumatoid arthritis: results from a large UK national cohort study. *Arthritis Rheum* 2007; **56**(1): 13-20.
48. Markenson JA, Gibofsky A, Palmer WR, et al. Persistence with anti-tumor necrosis factor therapies in patients with rheumatoid arthritis: observations from the RADIUS registry. *J Rheumatol* 2011; **38**(7): 1273-81.
49. Menter A, Papp KA, Gooderham M, et al. Drug survival of biologic therapy in a large, disease-based registry of patients with psoriasis: results from the Psoriasis Longitudinal Assessment and Registry (PSOLAR). *J Eur Acad Dermatol Venereol* 2016; **30**(7): 1148-58.
50. Zweegers J, van den Reek JM, van de Kerkhof PC, et al. Body mass index predicts discontinuation due to ineffectiveness and female sex predicts discontinuation due to side-effects in patients with psoriasis treated with adalimumab, etanercept or ustekinumab in daily practice: a prospective, comparative, long-term drug-survival study from the BioCAPTURE registry. *Br J Dermatol* 2016; **175**(2): 340-7.

FOOTNOTES

Contributors: All authors contributed to study design. SAM extracted, prepared the data and performed the analyses. SAM drafted the manuscript, with revisions by KK, SS, YS,BG,AMA,FAA and MB.

Funding: We acknowledge the support from The Farr Institute @ Scotland. The Farr Institute @ Scotland is supported by a 10-funder consortium: Arthritis Research UK, the British Heart Foundation, Cancer Research UK, the Economic and Social Research Council, the Engineering and Physical Sciences Research Council, the Medical Research Council, the National Institute of Health Research, the National Institute for Social Care and Health Research (Welsh Assembly Government), the Chief Scientist Office (Scottish Government Health Directorates), the Wellcome Trust, (MRC Grant No: MR/K007017/1).

Competing interest: SS has received honoraria from Janssen, Novartis, AbbVie, Pfizer, UCB, Celgene and Boehringer-Ingelheim; and research funding from UCB, Celgene, Boehringer-Ingelheim, BMS and Pfizer.

Patient consent: Not required.

Ethical approval: Ethics permission was not required as only non-identifiable routine data was used for this study. The study was approved by the NHS GGC Safe Haven Local Privacy Advisory Committee (Project number: GSH/15/MT/001). No patient identifiers were available to the study team and all data were accessed via the GGC Safe Haven.

Provenance and peer review: not commissioned; externally peer reviewed.

Data sharing statement: All data supporting this research are openly available to researchers based in recognized UK institutions from http://www.nhsggc.org.uk/about-us/professional-support-sites/nhsggc-safe-haven/your-research/available-datasets. For further information contact the Greater Glasgow and Clyde Safe Haven by emailing safehaven@ggc.scot.nhs.uk

For peer review only - http://bmjopen.bmj.com/site/about/guidelines.xhtml

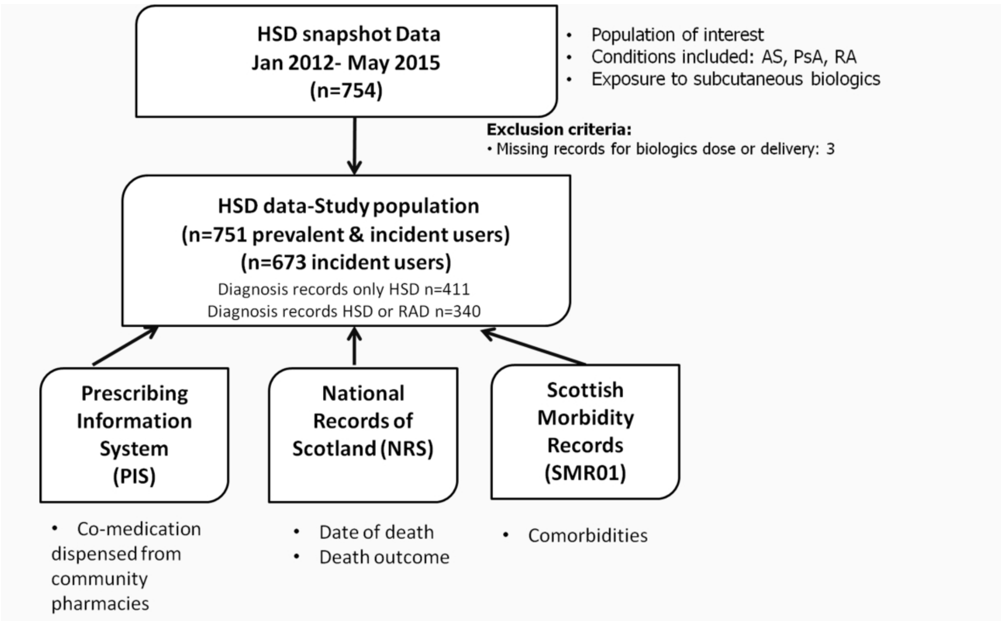

306x188mm (300 x 300 DPI)

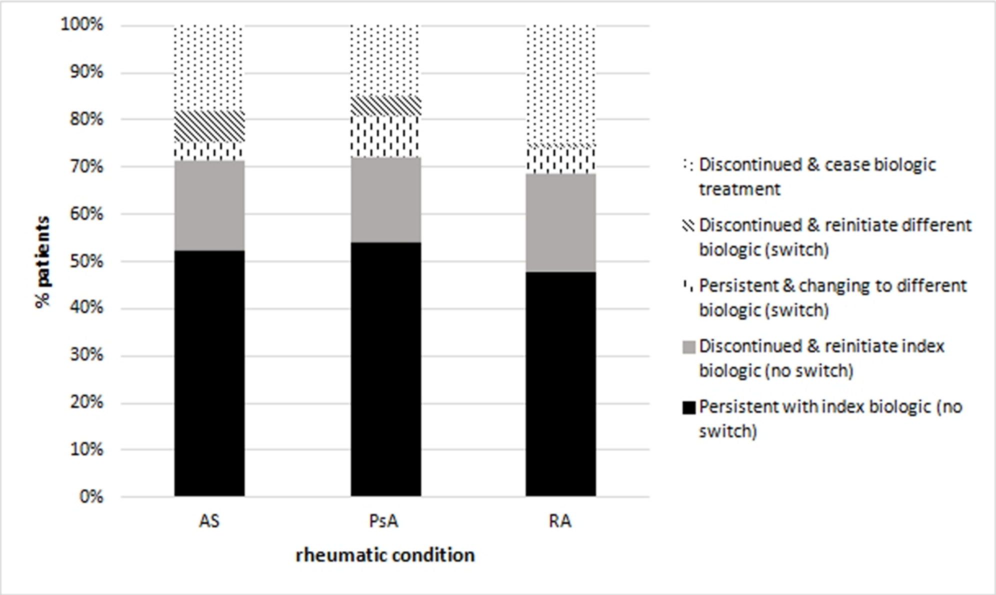

215x129mm (300 x 300 DPI)

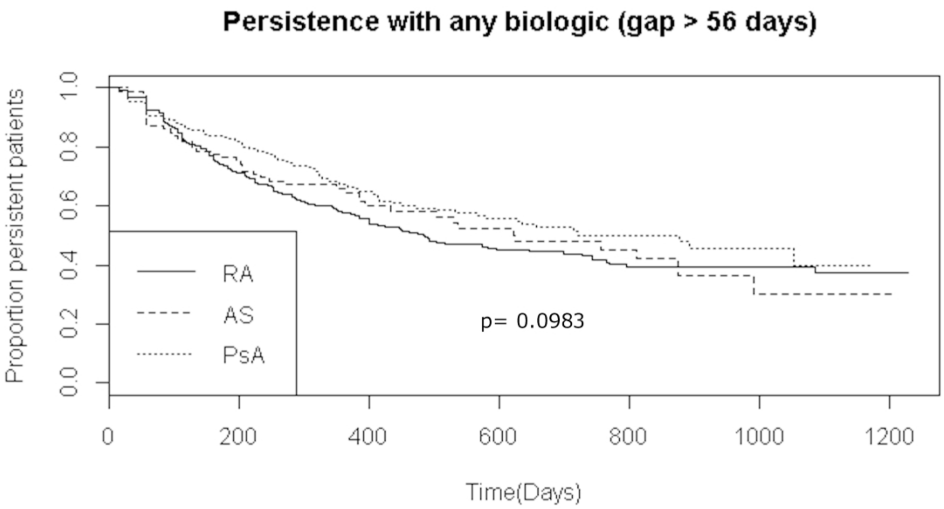

246x141mm (300 x 300 DPI)

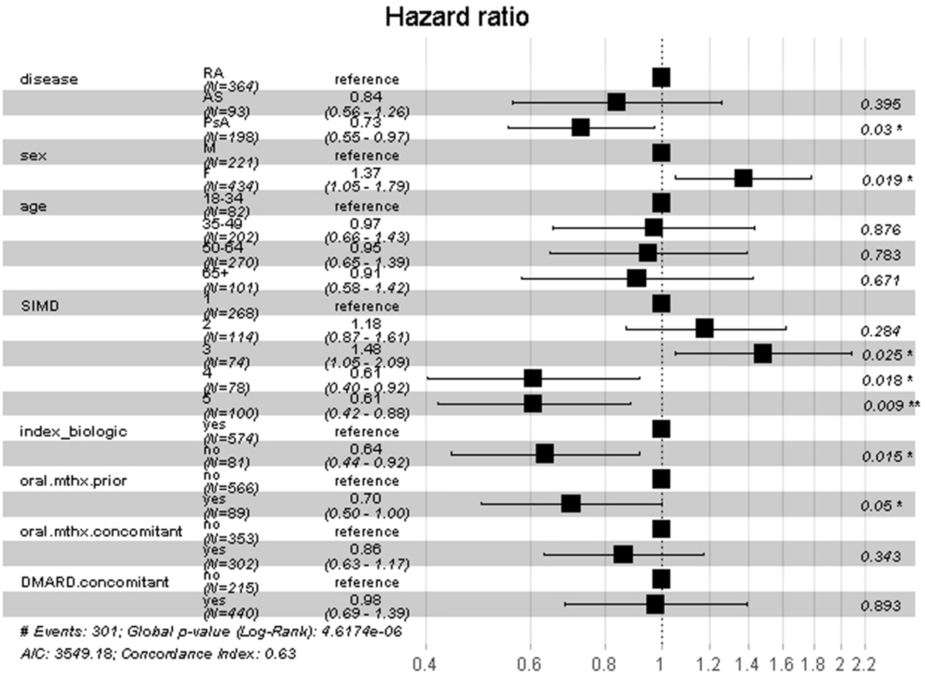

177x127mm (300 x 300 DPI)

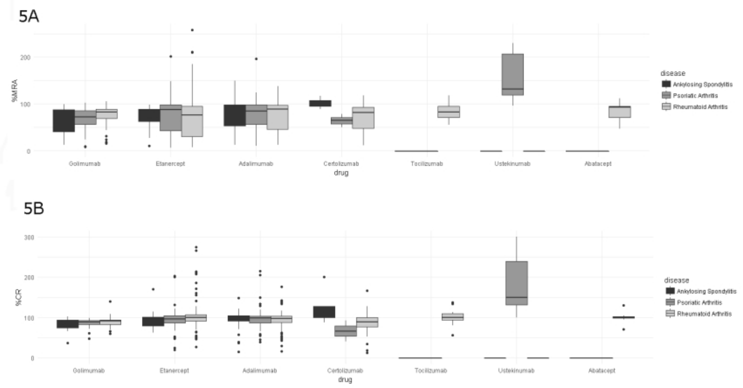

173x117mm (300 x 300 DPI)

Supplementary file :

Validation method and results

Of the 751 patients in HSD, 340 were also in the RAD. A minority of these (n=48) had diagnosis recorded in either of the datasets with the majority of these (n=292) with diagnosis recorded in both datasets (supplementary figure 1). RAD diagnosis was considered as the "gold standard" because it was collected in rheumatology clinics by clinical staff and was used to determine the validity of HSD diagnosis in the 292 patients.

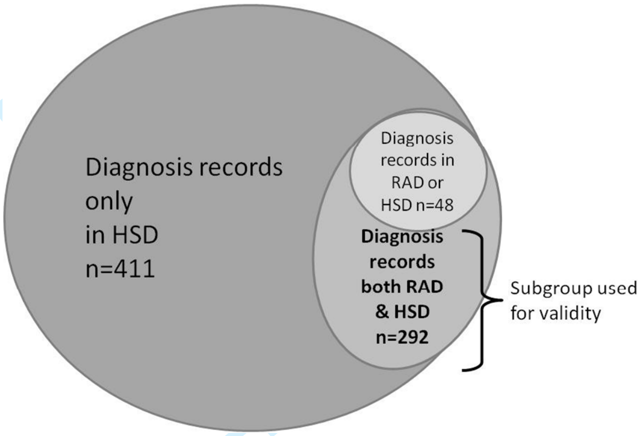

Supplementary Figure 1. Diagnoses of rheumatic conditions of the 751 patients according to HSD and RAD records.

Validity of diagnoses of rheumatic conditions (PPV and sensitivity)

The positive predictive value (PPV) and sensitivity with 95% confidence intervals were calculated for the diagnosis of RA, PsA or AS in HSD. PPV was calculated as the proportion of subjects in HSD with an indication for any of these three conditions in first recorded delivery and confirmed diagnosis in RAD. Sensitivity was calculated as the proportion of RAD cases who had a RA, PsA or AS diagnosis from all the RA, PsA or AS patients in the 292 subjects. A high PPV identifies a true population, or this case subgroup, of patients with chronic inflammatory diseases; while a high sensitivity allows to asses as many cases as possible.

Validity of arthritic conditions

From the subset of patients with arthritic conditions in HSD, the diagnosis was confirmed in 292 patients, with a 91.8%(95%CI: 86.4-95.1) PPV and a sensitivity of 97.3% (95%CI:93.3-99.0) for RA, 97.4% (95%CI:86.5-99.9) PPV and 94.9% (95%CI: 83.1-98.6)sensitivity for AS and 95.8% (95%CI:89.8-98.4) PPV and 88.5%(95%CI:80.9-93.3) sensitivity for PsA.

Many studies focus in the optimal clinical use and safety of medication in rheumatic diseases using administrative or claims data and one of the first requirements, following the best-practice consensus from the CANRAD Network, is the validation of case definitions.[1] There are mainly two types of validation methods (a) internal using algorithms or sensitivity analyses within the data or (b) external using data from other sources such as clinical notes or data captured by general practitioners.[2] In this study, an external validation was performed by comparing the diagnoses of the patients present in both HSD and RAD

For peer review only - http://bmjopen.bmj.com/site/about/guidelines.xhtml

databases. Both the PPV and sensitivity were above 80% for the three diseases. In contrast to several studies, instead of using ICD-10 codes, our study used free text entered by clinicians in RAD which was compared to the diagnoses entered from the prescriptions in HSD. All the patients also had at least one prescription for a subcutaneous biologic in HSD. Our sample size (n=292) is in the range of similar studies aimed to validate diagnoses (n=85-450), and our PPV values and sensitivities are comparable to others using data from rheumatology clinics (PPV 95-97%; sensitivities reported 30-100%).[3, 4]

Analyses with incident users of subcutaneous biologics

Incident users were defined as patients who did not receive a subcutaneous biologic in the first six months of the study period (from January-June 2012). This incident cohort included 673 patients (89.6% from the original cohort), from them 95 had AS, 208 PsA and 370 RA (Supplementary Table 1). The proportion of patients according to age, gender and the distribution of socioeconomic levels (SIMD) were similar.

A total of 88.9% of incident patients had more than one delivery of biologic during the study period (compared to 89.3% from the original cohort) and the same percentage of 83.2% did not switch biologic.

The index biologics mostly used were also adalimumab and etanercept. Although the proportion of users according to their index biologics did not change, there were less users of adalimumab, certolizumab pegol and etanercept in the incident cohort as the prevalent users of these three medications were excluded. This is in agreement with the dates of approval for their use as these biologics were the first ones approved and patients were more likely to be users before 2012.

The pattern of concomitant medications prevailed in the incident cohort as RA and PsA patients were frequent users of oral methotrexate, DMARDs and NSAIDs, while AS patients mainly used NSAIDs.

For peer review only

Supplementary Table 1. Baseline characteristics of incident patients with rheumatic conditions receiving subcutaneous biologic from July 2012-May 2015 (n=673)

Rheumatic disease	All		Ankylosing spondylitis		Psoriatic arthritis		Rheumatoid arthritis	
n	**673**		**95**		**208**		**370**	
**Female - n (%)**	**446**	(66.3)	**34**	(36.8)	**117**	(56.3)	**295**	(79.7)
**Age***								
Age (median, IQR)*	**52.9(42.5-60.4)**		**47.4 (35.6-55.4)**		**48.0(40.1-57.3)**		**55.9(47.1-64.2)**	
**18-34 (%)**	**87**	(12.9)	**21**	(22.1)	**34**	(16.3)	**32**	(8.6)
**35-49 (%)**	**196**	(29.1)	**31**	(32.6)	**83**	(39.9)	**82**	(22.2)
**50-64 (%)**	**282**	(41.9)	**35**	(36.8)	**75**	(36.1)	**172**	(46.5)
**65+ (%)**	**108**	(16.0)	**8**	(8.4)	**16**	(7.7)	**84**	(22.7)
SIMD*								
**1 Most deprived (%)**	**274**	(40.7)	**38**	(43.2)	**85**	(42.1)	**151**	(41.8)
**2 (%)**	**117**	(17.4)	**19**	(21.6)	**40**	(19.8)	**58**	(16.1)
**3 (%)**	**75**	(11.1)	**13**	(14.8)	**22**	(10.9)	**40**	(11.1)
**4 (%)**	**78**	(11.6)	**12**	(13.6)	**22**	(10.9)	**44**	(12.2)
**5 Least deprived (%)**	**107**	(15.9)	**6**	(6.8)	**33**	(16.3)	**68**	(18.8)
Number of deliveries of biologics								
1	**75**	(11.1)	**13**	(13.7)	**18**	(8.7)	**44**	(11.9)
2-10	**434**	(64.5)	**57**	(60.0)	**136**	(65.4)	**241**	(65.1)
11-20	**129**	(19.2)	**19**	(20.0)	**43**	(20.7)	**67**	(18.1)
21+	**35**	(5.2)	**6**	(6.3)	**11**	(5.3)	**18**	(4.9)

For peer review only - http://bmjopen.bmj.com/site/about/guidelines.xhtml

Number of different biologics delivered								
1	**560**	(83.2)	**81**	(85.3)	**165**	(79.3)	**314**	(84.9)
2, 3 or 4 (switchers)	**113**	(16.8)	**14**	(14.7)	**43**	(20.7)	**56**	(15.1)
First biologic delivered								
**Abatacept**	**8**	(1.2)	**NA**		**NA**		**8**	(2.2)
**Adalimumab**	**263**	(39.1)	**47**	(49.5)	**98**	(47.1)	**118**	(31.9)
**Certolizumab Pegol**	**68**	(10.1)	**6**	(6.3)	**<5**		**60**	(16.2)
**Etanercept**	**215**	(31.9)	**22**	(23.2)	**77**	(37.0)	**116**	(31.4)
**Golimumab**	**91**	(13.5)	**20**	(21.1)	**24**	(11.5)	**47**	(12.7)
**Tocilizumab**	**21**	(3.1)	**NA**		**NA**		**21**	(5.7)
**Ustekinumab**	**7**	(1.0)	**NA**		**7**	(3.4)	**NA**	
**Follow-up (days), median (range)**	**480**	**(264.0-734.0)**	**442 (278-749.5)**		**504.5(267.5-763.5)**		**482.0 (263.3-731.5)**	
Concomitant medication use 1 year prior first delivery								
**Antibacterial use**	**20**	(3.0)	**<5**		**<5**		**14**	(3.8)
**Antifungal use**	**<5**		**NA**		**NA**		**<5**	
**Antiviral use**	**NA**		**NA**		**NA**		**<5**	
**Oral methotrexate use**	**39**	(5.8)	**<5**		**9**	(4.3)	**29**	(7.8)
**Subcutaneous methotrexate use**	**NA**		**NA**		**NA**		**NA**	
**Any DMARD**	**54**	(8.0)	**<5**		**13**	(6.3)	**40**	(10.8)
**NSAID**	**54**	(8.0)	**8**	(8.4)	**15**	(7.2)	**31**	(8.4)
**Gluco or corticosteroids**	**10**	(2.5)	**NA**		**<5**		**9**	(2.4)

For peer review only - http://bmjopen.bmj.com/site/about/guidelines.xhtml

Concomitant medication use during study period								
**Antibacterial use**	**394**	(58.5)	**46**	(48.4)	**124**	(59.6)	**224**	(60.5)
**Antifungal use**	**62**	(9.2)	**7**	(7.4)	**22**	(10.6)	**33**	(8.9)
**Antiviral use**	**44**	(6.5)	**<5**		**16**	(7.7)	**26**	(7.0)
**Oral methotrexate use**	**284**	(42.2)	**7**	(7.4)	**78**	(37.5)	**199**	(53.8)
**Subcutaneous methotrexate use**	**10**	(1.5)	**NA**		**<5**		**7**	(1.9)
**Any DMARD**	**432**	(64.2)	**9**	(9.5)	**122**	(58.7)	**301**	(81.4)
**NSAID**	**430**	(63.9)	**65**	(68.4)	**137**	(65.9)	**228**	(61.6)
**Gluco or corticosteroids**	**132**	(19.6)	**10**	(10.5)	**26**	(12.5)	**96**	(25.9)
**Comorbidities**								
Charlson score								
0	**145**	(21.5)	**32**	(30.5)	**66**	(31.7)	**47**	(12.7)
1	**164**	(24.4)	**7**	(6.7)	**14**	(6.7)	**143**	(38.6)
2+	**33**	(4.9)	**<5**		**5**	(2.4)	**27**	(7.3)
unknown	**331**	(49.2)	**55**	(52.4)	**123**	(59.1)	**153**	(41.4)

*Age at first delivery, ** from 673 patients 22 did not have Scottish Index of Multiple Deprivation (SIMD) values. Follow-up is from first delivery to study end date. DMARD- disease-modifying anti-rheumatic drugs; NSAID- non-steroidal anti-inflammatory drug

For peer review only - http://bmjopen.bmj.com/site/about/guidelines.xhtml

Discontinuation and persistence

Also in the incident cohort approximately half of the patients were persistent on their index biologic throughout the study period (55% AS, 55% PsA, 49%RA) as shown in Supplementary Figure 2. From those who discontinue treatment the majority reintiate it with their index biologic (44%AS, 51% PsA, 43%RA) and a few switch to other biologics (13%AS, 8% PsA, 2%RA).

Supplementary Figure 2. Persistence and discontinuation (re-initiating or ceasing) of treatment with biologics according to rheumatic condition in the incident cohort (n=673)

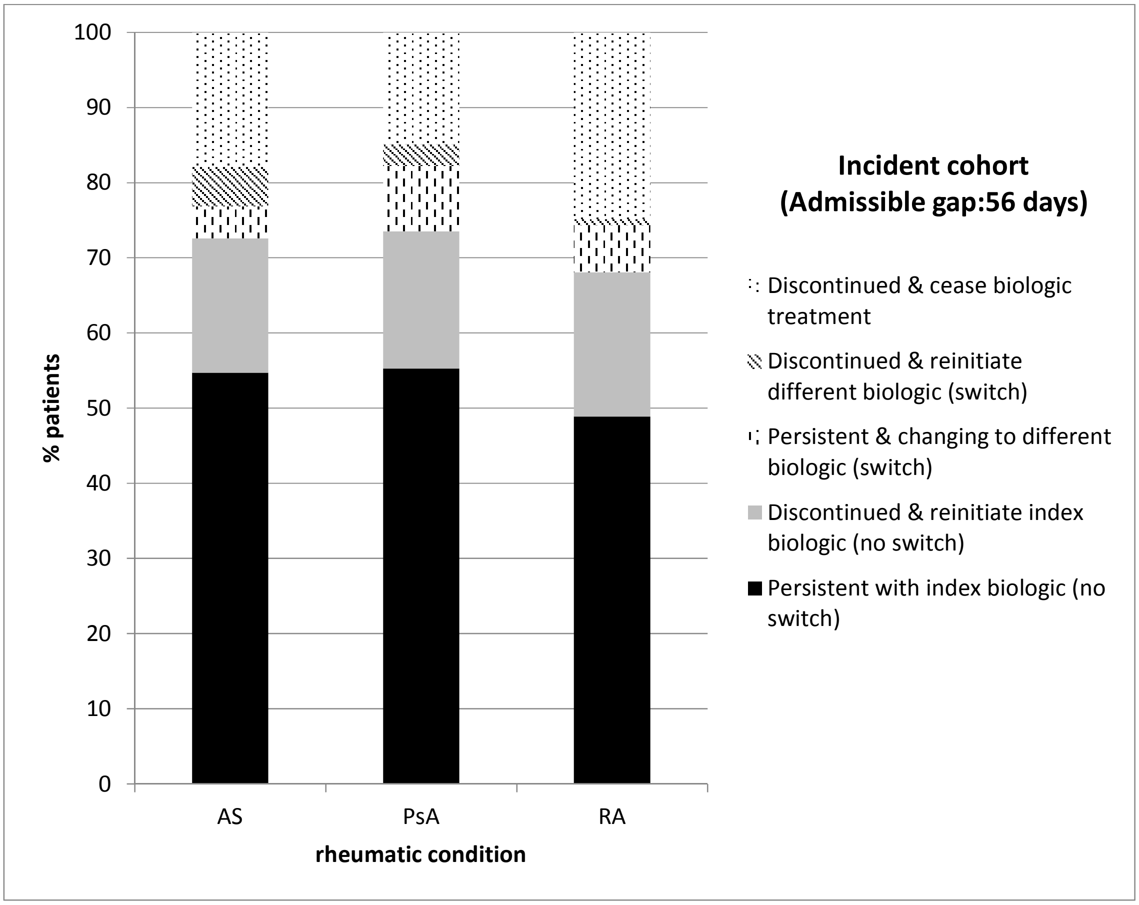

For peer review only - http://bmjopen.bmj.com/site/about/guidelines.xhtml

The crude survival curve of *time to discontinuation* (first gap greater than 56 days between biologic deliveries) of any biologic in the incident is shown in Supplementary Figure 3. In the crude model, the proportion of patients persisting is also slightly higher in PsA and AS compared to RA; however, there is no significant difference according to the log-rank test (p=0.165) and the Cox regression between RA and PsA (HR =0.77, 95%CI:0.58-1.01, p=0.0597).

Supplementary Figure 3:Crude survival curves comparing persistent patients according to rheumatic condition. (n=577)

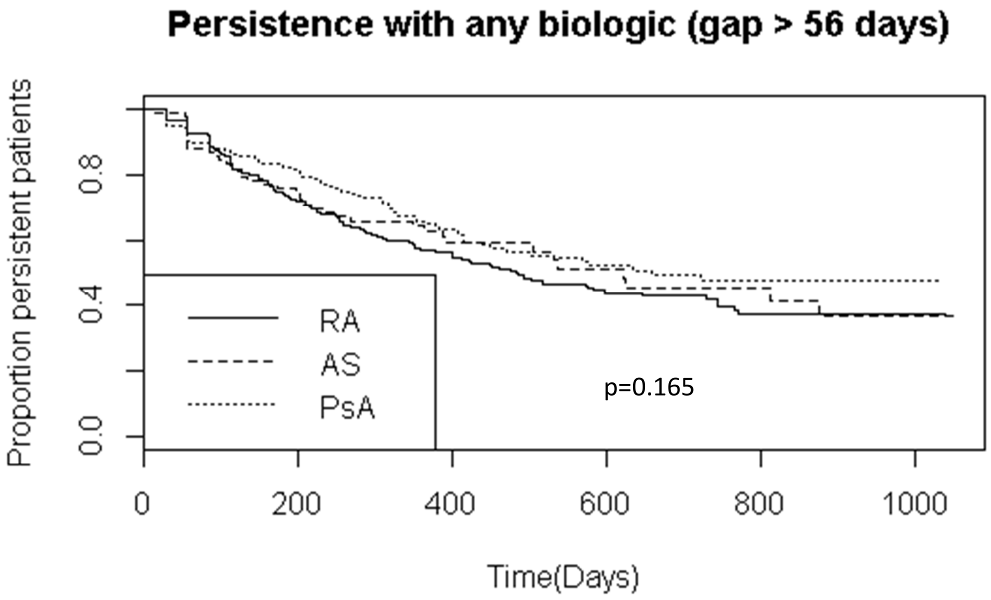

After adjustment for covariates, although a slight difference between RA and PsA patients was present, it was not significant (HR =0.74, 95%CI:0.54-1.00, p=0.062). Fewer covariates were significant in the incident cohort model. Female sex and SIMD 3 were associated with a higher risk of discontinuation, whereas SIMD 5 (least deprived quintile) and persisting with switched biologic (not index biologic) were associated with a lower risk of discontinuation as shown in Supplementary Figure 4. Age and the use of methotrexate or DMARD prior or during the study were not statistically significant covariates.

For peer review only - http://bmjopen.bmj.com/site/about/guidelines.xhtml

Supplementary Figure 4: Adjusted Hazard Ratios for persistence with biologics using Cox regression analysis excluding patients without SIMD values (n=556)

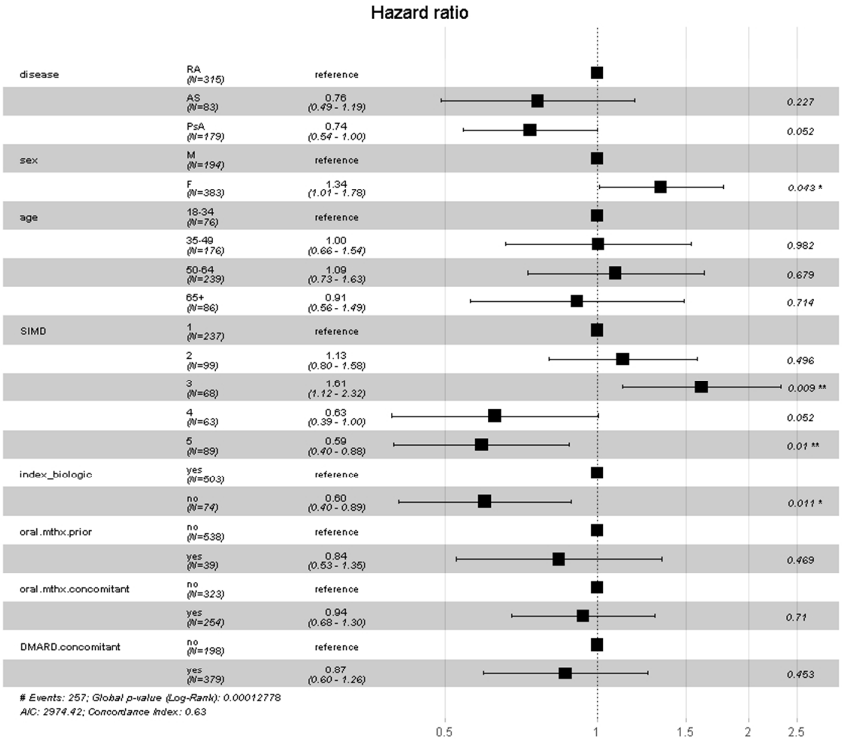

For peer review only - http://bmjopen.bmj.com/site/about/guidelines.xhtml

Adherence

Incident patients with at least two deliveries of the first (index) biologic (n = 598, 88.9%) were used to evaluate adherence. With overall adherence over 80% and both MRA and CR results showing the same patterns as the overall cohort.

Supplementary Figure 5: Adherence to biologics by rheumatic condition according to %MRA (5A) and %CR (5B). MRA- medication refill adherence; CR- compliance rate.

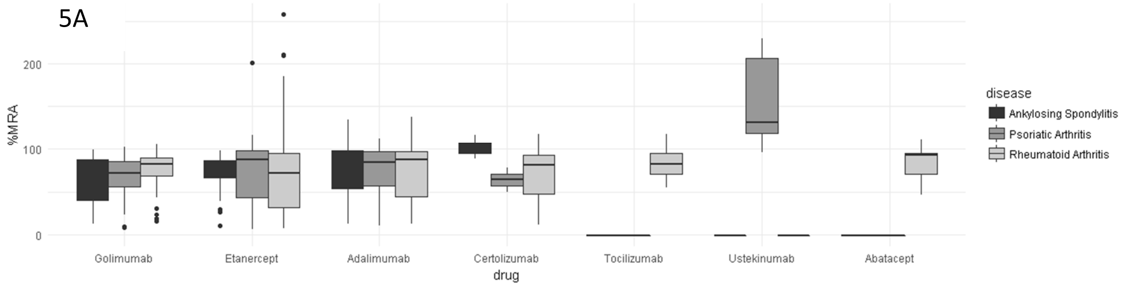

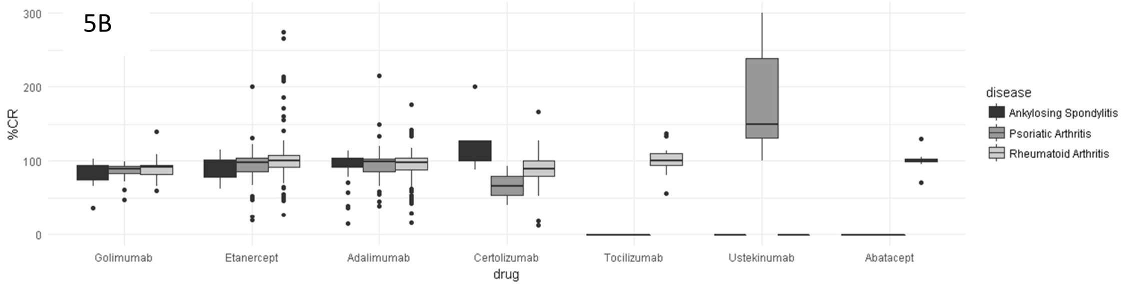

For peer review only - http://bmjopen.bmj.com/site/about/guidelines.xhtml

Sensitivity analyses discontinuation and persistence

Supplementary Figure 6. Persistence and discontinuation (re-initiating or ceasing) of treatment with biologics according to rheumatic condition in all the cohort with a gap of 28 days (Fig.6A) or 84 days (Fig.6B) and in the incident cohort with a gap of 28 days (Fig.6C) or 84 days (Fig.6D)

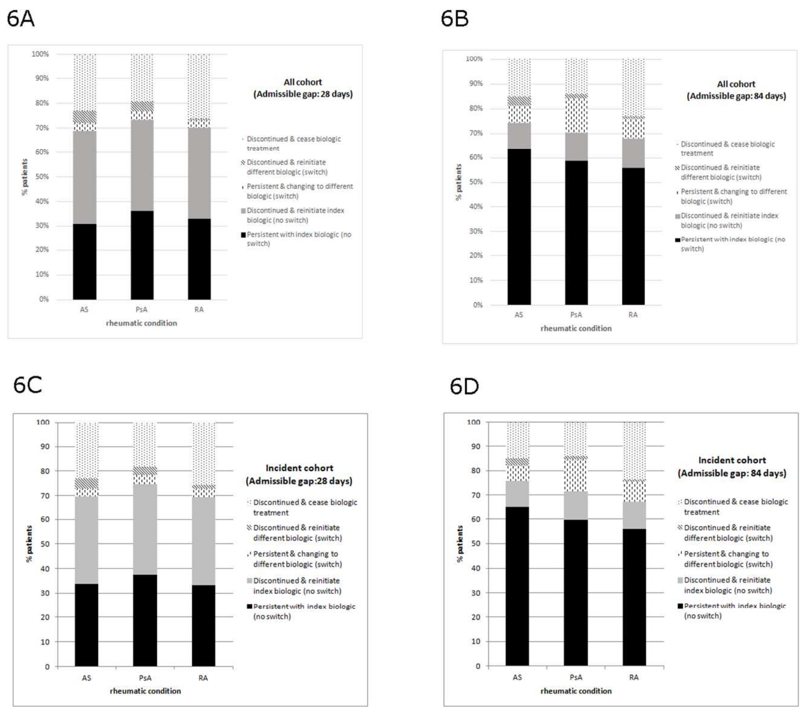

References

1. Widdifield J, Labrecque J, Lix L, et al. Systematic review and critical appraisal of validation studies to identify rheumatic diseases in health administrative databases. *Arthritis Care Res* 2013; **65**(9): 1490-503.
2. Herrett E, Thomas SL, Schoonen WM,et al. Validation and validity of diagnoses in the General Practice Research Database: a systematic review. *Br J Clin Pharmacol* 2010; **69**(1): 4-14.
3. Dubreuil M, Peloquin C, Zhang Y,et al. Validity of Ankylosing Spondylitis Diagnoses in The Health Improvement Network. *Pharmacoepidemiol Drug Saf* 2016; **25**(4): 399-404.
4. Thomas SL, Edwards CJ, Smeeth L, et al. How accurate are diagnoses for rheumatoid arthritis and juvenile idiopathic arthritis in the general practice research database? *Arthritis Rheum* 2008; **59**(9): 1314-21.

only

For peer review only - http://bmjopen.bmj.com/site/about/guidelines.xhtml

STROBE 2007 (v4) Statement—Checklist of items that should be included in reports of *cohort studies*

Section/Topic	Item #	Recommendation	Reported on page #
**Title and abstract**	1	(*a*) Indicate the study's design with a commonly used term in the title or the abstract	1
		(*b*) Provide in the abstract an informative and balanced summary of what was done and what was found	2
**Introduction**			
Background/rationale	2	Explain the scientific background and rationale for the investigation being reported	3
Objectives	3	State specific objectives, including any prespecified hypotheses	3
**Methods**			
Study design	4	Present key elements of study design early in the paper	4
Setting	5	Describe the setting, locations, and relevant dates, including periods of recruitment, exposure, follow-up, and data collection	4-5
Participants	6	(*a*) Give the eligibility criteria, and the sources and methods of selection of participants. Describe methods of follow-up	4-5
		(*b*) For matched studies, give matching criteria and number of exposed and unexposed	NA
Variables	7	Clearly define all outcomes, exposures, predictors, potential confounders, and effect modifiers. Give diagnostic criteria, if applicable	5-6
Data sources/ measurement	8*	For each variable of interest, give sources of data and details of methods of assessment (measurement). Describe comparability of assessment methods if there is more than one group	4-6
Bias	9	Describe any efforts to address potential sources of bias	13
Study size	10	Explain how the study size was arrived at	4
Quantitative variables	11	Explain how quantitative variables were handled in the analyses. If applicable, describe which groupings were chosen and why	5
Statistical methods	12	(*a*) Describe all statistical methods, including those used to control for confounding	5-6
		(*b*) Describe any methods used to examine subgroups and interactions	5-6
		(*c*) Explain how missing data were addressed	5
		(*d*) If applicable, explain how loss to follow-up was addressed	NA
		(*e*) Describe any sensitivity analyses	4-5, supplementary file

For peer review only

**Results**			
Participants	13*	(a) Report numbers of individuals at each stage of study—eg numbers potentially eligible, examined for eligibility, confirmed eligible, included in the study, completing follow-up, and analysed	4
		(b) Give reasons for non-participation at each stage	4
		(c) Consider use of a flow diagram	4
Descriptive data	14*	(a) Give characteristics of study participants (eg demographic, clinical, social) and information on exposures and potential confounders	6-9
		(b) Indicate number of participants with missing data for each variable of interest	7-9
		(c) Summarise follow-up time (eg, average and total amount)	8
Outcome data	15*	Report numbers of outcome events or summary measures over time	10
Main results	16	(*a*) Give unadjusted estimates and, if applicable, confounder-adjusted estimates and their precision (eg, 95% confidence interval). Make clear which confounders were adjusted for and why they were included	10-11
		(*b*) Report category boundaries when continuous variables were categorized	7-9
		(*c*) If relevant, consider translating estimates of relative risk into absolute risk for a meaningful time period	NA
Other analyses	17	Report other analyses done—eg analyses of subgroups and interactions, and sensitivity analyses	10-11, supplementary file
**Discussion**			
Key results	18	Summarise key results with reference to study objectives	11
**Limitations**			
Interpretation	20	Give a cautious overall interpretation of results considering objectives, limitations, multiplicity of analyses, results from similar studies, and other relevant evidence	12-14
Generalisability	21	Discuss the generalisability (external validity) of the study results	13
**Other information**			
Funding	22	Give the source of funding and the role of the funders for the present study and, if applicable, for the original study on which the present article is based	17

*Give information separately for cases and controls in case-control studies and, if applicable, for exposed and unexposed groups in cohort and cross-sectional studies.

For peer review only

Note: An Explanation and Elaboration article discusses each checklist item and gives methodological background and published examples of transparent reporting. The STROBE checklist is best used in conjunction with this article (freely available on the Web sites of PLoS Medicine at http://www.plosmedicine.org/, Annals of Internal Medicine at http://www.annals.org/, and Epidemiology at http://www.epidem.com/). Information on the STROBE Initiative is available at www.strobe-statement.org.

For peer review only

BMJ Open

Discontinuation, persistence and adherence to subcutaneous biologics delivered via a homecare route to Scottish adults with rheumatic diseases: a retrospective study.

Journal:	*BMJ Open*
Manuscript ID	bmjopen-2018-027059.R1
Article Type:	Research
Date Submitted by the Author:	29-Mar-2019
Complete List of Authors:	Alvarez-Madrazo, Samantha; University of Strathclyde, Health Data Research Scotland,Strathclyde Institute of Pharmacy and Biomedical Sciences<br>Kavanagh, Kimberley; University of Strathclyde Department of Mathematics and Statistics<br>Siebert, Stefan<br>Semple, Yvonne; University of Strathclyde, Strathclyde Institute of Pharmacy and Biomedical Sciences; Glasgow Royal Infirmary, Medicines Information, Pharmacy Department<br>Godman, Brian; Karolinska University Hospital, 5 Division of Clinical Pharmacology, Karolinska Institutet,<br>Maciel Almeida, Alessandra; Universidade Federal de Minas Gerais<br>Acurcio, Francisco ; Universidade Federal de Minas Gerais<br>Bennie, Marion; University of Strathclyde, Health Data Research Scotland, Strathclyde Institute of Pharmacy and Biomedical Sciences; NHS National Services Scotland, Information Services Division
**Primary Subject Heading**:	Rheumatology
Secondary Subject Heading:	Health informatics, Pharmacology and therapeutics
Keywords:	biologics, psoriatic arthritis, rheumatoid arthritis, ankylosing spondylitis, persistence, adherence

SCHOLARONE™
Manuscripts

TITLE PAGE

Title: Discontinuation, persistence and adherence to subcutaneous biologics delivered via a homecare route to Scottish adults with rheumatic diseases: a retrospective study

Authors:

[1]Samantha Alvarez-Madrazo

[2]Kimberley Kavanagh

[3]Stefan Siebert

[1,4]Yvonne Semple

[1,5] Brian Godman

[6]Alessandra Maciel Almeida

[6]Francisco de Assis Acurcio

[1,7] Marion Bennie

Author affiliations:

[1]Health Data Research Scotland, Strathclyde Institute of Pharmacy and Biomedical Sciences, University of Strathclyde, Glasgow, UK
[2] Department of Mathematics and Statistics, University of Strathclyde, Glasgow,UK
[3] Institute of Infection Immunity and Inflammation, University of Glasgow,UK
[4] Medicines Information, Pharmacy Department, Glasgow Royal Infirmary,UK
[5] Division of Clinical Pharmacology, Karolinska Institutet, Karolinska University Hospital, Stockholm, Sweden
[6] Federal University of Minas Gerais, Brazil
[7] Information Services Division, NHS National Services Scotland, Edinburgh,UK

Corresponding author:

Prof. Marion Bennie

Strathclyde Institute of Pharmacy and Biomedical Sciences

University of Strathclyde

161 Cathedral Street

Glasgow

G4 0RE

Email: marion.bennie@strath.ac.uk

Tel. +44(0) 141 548 2113

Word count: 3988

For peer review only

ABSTRACT

Objectives*:* to understand patterns of subcutaneous (SC) biologic use over time in adults with inflammatory rheumatic musculoskeletal diseases receiving a homecare delivery service.

Design: retrospective cohort

Setting: patients in secondary care receiving SC biologics in the largest Scottish Health Board

Participants: a new bespoke cohort was created from routine data gathered as part of a health board Homecare Service Database. Patients over 18 years who received a supply of SC biologic from January 2012 - May 2015 with a diagnosis for rheumatoid arthritis (RA), psoriatic arthritis (PsA) or ankylosing spondylitis (AS) were included.

Outcomes measured: a standardised framework was applied by measuring discontinuation rates, persistence using Kaplan-Meier analysis and Cox regression and adherence using medication refill adherence (MRA) and Compliance Rate (CR).

Results: 751 patients were identified (AS – 105, PsA – 227, RA - 419) of whom 89.3% had more than one biologic delivery (median days' follow-up: AS - 494; PsA – 544; RA - 529) and 83.2% did not switch biologic. For all conditions, approximately half were persistent on their index biologic (52% AS, 54% PsA, 48%RA). Of patients who discontinued treatment, the majority re-initiated with the same biologic (19% AS, 18% PsA and 21% RA). Overall adherence during the period of treatment was over 80% when calculated using MRA (median %MRA - AS 84.0%, PsA 85.0%, RA 82.4%) or CR (median %CR - AS 96.6%, PsA 97%, RA 96.6%).

Conclusion: Use of linked routine data is a sustainable pathway to enable on-going evaluation of biologics use. A more consistent approach to studying use (discontinuation, persistence and adherence metrics) should be adopted to enable comparability of studies.

Keywords: biologics, psoriatic arthritis, rheumatoid arthritis, ankylosing spondylitis,persistence, adherence

Article summary

Strengths and limitations of this study

- This is the first real-world cohort containing type, frequency and dose of seven subcutaneous biologics delivered directly to patients with ankylosing spondylitis, psoriatic arthritis and rheumatoid arthritis.
- The cohort is sustainable being generated as part of routine clinical care, can be easily updated and linked to other administrative datasets and provides a novel way to assess adherence, persistence and discontinuation of biologics.
- The limitations include having a mixed cohort of naive and existing biologic users, not capturing reasons for variablity in patients' response, use of intravenous biologics or prior use of any biologic or subcutaneous methotrexate.
- The generalisability of the results in a Scottish setting has to be tested, as this cohort covers the largest Scottish Health Board.

For peer review only - http://bmjopen.bmj.com/site/about/guidelines.xhtml

For peer review only

INTRODUCTION

Chronic inflammatory rheumatic musculoskeletal diseases such as rheumatoid arthritis (RA), psoriatic arthritis (PsA) and ankylosing spondylitis (AS) are characterised by inflammatory joint symptoms requiring long-term management. Over the past two decades, the use of biologic therapies has revolutionised their treatment, with national and global groups recommending their use in patients with moderate to severe disease unresponsive to conventional therapies.[1-5] Whilst clinical trials have demonstrated their short-term efficacy and safety, long-term evidence require observational studies and real-world data. Despite reassuring results to-date,[6-10] important challenges and unanswered questions remain.

Furthermore, despite the apparent shared inflammatory mechanisms and therapies, there are significant differences between these inflammatory conditions in terms of prevalence, gender and sex distribution, genetics, pathophysiology and clinical phenotype, including extra-articular manifestations and co-morbidities.[11-16] Therefore, the safety and long-term therapy data for one condition (usually RA due to the larger numbers and longer pharmacological history) cannot simply be assumed to apply to the other conditions, even for the same drug. It is therefore important to evaluate these factors in real-world settings across the range of conditions for which these drugs are used.

In addition to drug and disease-related factors, in clinical practice poor adherence and persistence to therapy are important detrimental factors, contributing to treatment failure and disease progression.[17] Most of the direct comparisons for adherence and persistence are from observational studies, mainly focusing on the first biologics to reach the market/clinical practice (infliximab, adalimumab and etanercept).[17-19] A few studies have incorporated other biologics,[20-26] with more biologics set to be approved in the upcoming years. Although the standardised measurement of adherence and persistence, dividing the process in three phases - initiation, implementation and discontinuation - has been advocated by Vrijens *et al.* and endorsed by the European Society for Patient Adherence, Compliance and Persistence (ESPACOMP),[27] this methodology has mainly been applied to oral medications and not to biologics, which are administered subcutaneously or intravenously, with complex dosing schedules.

Disease or drug registers are expensive, labour-intensive and not optimal for evaluating adherence and persistence, so other methods are required.[17, 18] In many countries, administrative or prescription datasets linked to reimbursement have been used,[28-31] but these do not always indicate whether the participant actually received the medication. Furthermore, biologics usually require specialist prescription so are not routinely captured in primary care prescription datasets.

This study aimed to use a homecare service database for subcutaneous biologics to understand the patterns of biologic use over time in a population of adults with rheumatic diseases receiving biologics.

For peer review only - http://bmjopen.bmj.com/site/about/guidelines.xhtml

METHODS

Data Sources

For this study a new bespoke, retrospective cohort of patients receiving subcutaneous biologics was created by linking administrative electronic health records (EHRs) at a patient level via an unique national (Scottish) health service identifier, known as the Community Health Index (CHI).[32] This dataset included two resources available in the largest Scottish Health Board, NHS Greater Glasgow and Clyde (GGC), with a population covering 1.2 million of the 5.4 million residents in Scotland.[33] The two novel resources were the Homecare Service Database (HSD) and the local Rheumatology Arthritis Database (RAD), in addition to three existing nationally held datasets: the Prescribing Information System (PIS),[34] the mortality records (National Records of Scotland-NRS) and the Scottish Morbidity Records (SMR01).[35] The variables obtained from each dataset are outlined in Figure 1.

Figure 1. Variables obtained from different Scottish datasets.

Of the GGC patients receiving biologics up until early 2018, 98% received subcutaneous biologics uniquely via the homecare delivery route, with delivery details captured in the HSD and managed through the pharmacy distribution system in the health board. Since 2012 the HSD has systematically captured information about patients (e.g. hospital attended, indication for biologics, drugs and dose, administration and delivery frequency, date and quantity delivered), prescribers and healthcare delivery companies. The remaining 2% of patients receiving intravenous biologics are not captured in the HSD as these medicines are administered directly in hospital day wards.

The RAD contains information of patients who have attended rheumatology clinics in two of the four main hospitals in Glasgow since 1997. Diverse clinical variables are recorded, including the main and secondary diagnoses, biologic screening/follow up, comorbidities, other medications and lifestyle factors.

The PIS includes prescriptions prescribed, dispensed and reimbursed within the community setting,[34] NRS captures cause-specific mortality records and SMR01 has episode-level data on all hospital inpatient and day care episodes from hospitals in Scotland.[35]

Study population

Patients who received at least one delivery of subcutaneous biologics in the HSD dataset with a diagnosis for AS, PsA or RA were eligible for this study. The RAD database was used to validate the diagnosis in a subset of HSD patients (see supplementary file and Supplementary Figure 1 for validation methods and results).

All patients who had a biologic supply date between January 2012 and May 2015 and were at least 18 years old at cohort entry were included. All patients were followed-up until death, migration to a primary care practice outside Scotland or end date of the study (May 2015), whichever occurred first. In the overall cohort, both prevalent and incident users were included (n=751, 105 AS, 227 PsA and 419 RA). To illustrate the stability of findings, a subset analysis was carried out with only incident users (n=673, 95 AS, 208 PsA and 370 RA), defined as patients who did not receive a subcutaneous biologic in the first six months of the study period (from January-June 2012) (online supplementary file).

For peer review only - http://bmjopen.bmj.com/site/about/guidelines.xhtml

One patient was excluded as its first biologic delivery was prior to the date of approval for the drug by the Scottish Medicines Consortium (SMC), which is the national health technology assessment process for all new medicines in Scotland.[36] Also two patients having all missing records in the HSD for dose, frequency or how often the delivery of the biologics would take place were deleted to enable calculations of weeks covered per delivery in the rest the cohort.

The first delivery date for any biologic was established as the index date and the first biologic recorded was considered the index biologic. Age was calculated at index date. Socioeconomic status was assessed using the Scottish Index of Multiple Deprivation quintile (SIMD) 2012 quintiles, a measure that incorporates different aspects of deprivation into a single index. Patients with missing SIMD values remained in the cohort. To assess co-medication, patients with at least one prescription in the 12 months prior to the index date in PIS for antibacterials (British National Formulary- BNF section 5.1), antifungals (BNF section 5.2), antivirals (BNF section 5.3), oral methotrexate (prescribed item methotrexate), Disease-Modifying Anti-Rheumatic Drugs (DMARDs - BNF subsection 10.1.3), Non-Steroidal Anti-inflammatory Drugs (NSAIDs - BNF subsection 10.1.1) and corticosteroids (BNF subsections 1.5.2 or 6.3.2) were recorded.

Data analyses

Patients characteristics were summarized using descriptive statistics. Medians and interquartile ranges are provided for continuous variables and global comparisons were done using the Kruskal-Wallis test. Numbers and percentages are provided for categorical variables and global comparisons were done using either chi-square test or Fisher's exact test (in cases where cells had expected frequencies < 5). To protect confidentiality in the results the GGC Safe Haven carried out a statistical disclosure control. The cell suppression method was applied when the cell values were small and there was a risk of disseminating sensitive information.[37]

In this study, we advocate the use of a standardised framework that includes measurements of discontinuation, persistence and adherence.[27, 38]

Considering the variation in the administration frequencies and number of injections delivered according to biologic, first the estimated days covered per delivery was calculated by multiplying the quantity delivered by the standard administration frequency according to the approved licence for each biologic. Knowing the delivery date and the estimated days covered allowed estimation of the expected end date covered and how it related with the following delivery, and whether there were gaps or not. These metrics were the basis of the utilisation analysis.

Discontinuation was defined as the end of treatment, either because there was a considerable gap of more than 56 days between deliveries (re-initiation) or no more deliveries were received (cessation). Those who did not discontinue were defined as persistent. Discontinuation rates were estimated using the refill-gap method.[39] Patients were censored at first discontinuation, pre-defined as a gap of more than 56 days with no biologics delivery after exhaustion of the days' supplied by the previous delivery, regardless of whether or not patients switched to another biologic. This gap was selected because most biologics were delivered bimonthly, approximately 5 times the half-life for the biologics of

For peer review only - http://bmjopen.bmj.com/site/about/guidelines.xhtml

interest would have elapsed, and this was comparable to other studies.[26, 29] A sensitivity analysis was conducted using a gap of 28 days (minimum period between deliveries) and 84 days (maximum period between deliveries) (see supplementary file online).

Crude persistence was estimated using Kaplan-Meier analysis and Cox regression were used to assess persistence post-index date, in patients with at least 6 months follow-up.The proportional hazards (PH) assumption for a Cox regression model was tested in both the crude and the adjusted model,and stratified where appropriate to ensure a non-significant global p value.[40] Patients who died or migrated were censored.

The hazard ratios (HRs) for persistence with biologics were adjusted for sex, age, SIMD, use of index biologic or not (meaning a switch could have happened whilst being persistent), use of oral methotrexate in the 12 months before to the index date, and concomitant use of oral methotrexate or DMARD during study period.

Adherence is defined as to what extent the biologics delivered correspond to the treatment regimen recommended in the BNF.There is no consensus in the literature on how best to estimate adherence thus two measurements were used for patients with at least two deliveries of the index biologic: medication refill adherence (MRA) and compliance rate (CR).[38, 41] MRA gives an overall adherence percentage describing the exposure during the whole study period regardless of the time when the last prescription was delivered and is calculated as (total days' supply/total days in study) x 100. In contrast, CR describes the exposure between first and last prescription delivered (total days' supply excluding last delivery/days first up to but not including last delivery) x 100.

Analyses were performed in R software, version 3.5.0.[42]

Patient and public involvement

The prelimary design of this study was presented to the Farr Scotland Public Panel. According to their experience, and preferences the research questions and outcome measures were developed to make the results generalisable to patients with rheumatic conditions.

RESULTS

The study included 751 patients: 105 with AS, 227 with PsA and 419 with RA (Table 1). From them 673 patients (89.6%) were incident users, 95 AS (90.5%), 208 PsA (91.6%) and 370 RA (88.3%).The patients with RA were older and had a higher proportion of females. Regardless of diagnosis, the majority of patients receiving biologics resided in the most deprived areas.

From the overall cohort, a total of 89.3% of patients had more than one delivery of biologic during the study period and 83.2% did not switch biologic. Patients were most commonly started on adalimumab or etanercept as index biologic. The median days' follow-up on biologic therapy, regardless of whether or not the patients switched to others biologics, was: 494 days in AS; 544 days in PsA and 529 days in RA patients.

For peer review only - http://bmjopen.bmj.com/site/about/guidelines.xhtml

Use of concomitant non-biologic medication during the study period was more frequent amongst patients with RA with oral methotrexate (54.9%), any DMARDs (81.6%) and NSAIDs (63.2%) most commonly prescribed. A similar pattern of medication use was observed in PsA patients. In contrast, in AS patients, NSAIDs (71.4%) were the most commonly co-prescribed medications. Prescription of at least one course of antibiotics in patients during the study period was similar for the three conditions (RA – 62.5%, PsA – 59.9%, AS – 49.5%). Similar trends of concomitant medications were found in the incident cohort (see Supplementary Table 1).

For peer review only

Table 1. Baseline characteristics of patients with rheumatic conditions receiving subcutaneous biologic from Jan 2012- May 2015 (n=751)

Rheumatic disease	All		Ankylosing spondylitis		Psoriatic arthritis		Rheumatoid arthritis		p-value
n	**751**		**105**		**227**		**419**		
**Female - n (%)**	**497**	(66.2)	**35**	(33.3)	**130**	(56.5)	**332**	(78.7)	2.2×10^{-16}
**Age***									
Age (median, IQR)*	**53.0(42.7-60.9)**		**47.2 (35.4-55.0)**		**48.0(40.2-57.3)**		**56.0(47.4-64.2)**		4.3×10^{-14}
**Age by category**									5.3×10^{-12}
**18-34 (%)**	**93**	(12.4)	**24**	(22.9)	**36**	(15.9)	**33**	(7.9)	
**35-49 (%)**	**222**	(29.6)	**36**	(34.3)	**91**	(40.1)	**95**	(22.7)	
**50-64 (%)**	**313**	(41.7)	**36**	(34.3)	**82**	(36.1)	**195**	(46.5)	
**65+ (%)**	**123**	(16.4)	**9**	(8.6)	**18**	(7.9)	**96**	(22.9)	
SIMD †									0.326
**1 Most deprived (%)**	**305**	(40.6)	**42**	(42.9)	**93**	(42.1)	**170**	(41.5)	
**2 (%)**	**132**	(17.6)	**21**	(21.4)	**44**	(19.9)	**67**	(16.3)	
**3 (%)**	**81**	(10.8)	**13**	(13.3)	**23**	(10.4)	**45**	(11)	
**4 (%)**	**93**	(12.4)	**15**	(15.3)	**25**	(11.3)	**53**	(12.9)	
**5 Least deprived (%)**	**118**	(15.7)	**7**	(7.1)	**36**	(16.3)	**75**	(18.3)	
Number of deliveries of biologics									
Number of deliveries of biologics (median, IQR)	**6(3-12)**		**7(3-11)**		**6(3-12)**		**6(3-12)**		0.579
Number of deliveries of biologics by category (%)									0.752

1	**80**	(10.7)	**14**	(13.3)	**19**	(8.4)	**47**	(11.2)	
2-10	**456**	(60.7)	**59**	(56.2)	**141**	(62.1)	**256**	(61.1)	
11-20	**156**	(20.8)	**25**	(23.8)	**49**	(21.6)	**82**	(19.6)	
21+	**59**	(7.8)	**7**	(6.7)	**18**	(7.9)	**34**	(8.1)	
Number of different biologics delivered (%)									0.108
1	**625**	(83.2)	**89**	(84.8)	**179**	(78.9)	**357**	(85.2)	
2, 3 or 4 (switchers)	**126**	(16.8)	**16**	(15.2)	**48**	(21.1)	**62**	(14.8)	
First biologic delivered (%)									$8.3x10^{-13}$
**Adalimumab**	**305**	(40.6)	**55**	(52.4)	**112**	(49.3)	**138**	(32.9)	
**Certolizumab Pegol**	**77**	(10.3)	**6**	(5.7)	**<5**		**69**	(16.5)	
**Etanercept**	**240**	(32)	**24**	(22.9)	**82**	(36.1)	**134**	(32)	
**Golimumab**	**93**	(12.4)	**20**	(19)	**24**	(10.6)	**49**	(11.7)	
**Abatacept**	**8**	(1.1)	**NA**		**NA**		**8**	(1.9)	NA[‡]
**Tocilizumab**	**21**	(2.8)	**NA**		**NA**		**21**	(5)	
**Ustekinumab**	**7**	(0.9)	**NA**		**7**	(3.1)	**NA**		
**Follow-up (days), median (range)**	**529**	**(284.0-852.0)**	**494 (285-838)**		**544 (280-831)**		**529 (283.5-866.5)**		0.894
Concomitant medication use within one year before index date (%)									$6.8x10^{-3}$
**Antibacterial use**	**48**	(6.4)	**6**	(5.7)	**11**	(4.8)	**31**	(7.4)	
**Oral methotrexate use**	**89**	(11.9)	**<5**		**23**	(10.1)	**63**	(15)	
**Any DMARD**	**115**	(15.3)	**<5**		**28**	(12.3)	**84**	(20)	

**NSAID**	**114**	(15.2)	**17**	(16.2)	**28**	(12.3)	**69**	(16.5)	
**Gluco or corticosteroids**	**19**	(2.5)	**<5**		**<5**		**16**	(3.8)	
**Antifungal use**	**<10**		**NA**		**<5**		**6**	(1.4)	NA‡
**Antiviral use**	**<5**		**NA**		**NA**		**<5**		
**Subcutaneous methotrexate use**	**NA**		**NA**		**NA**		**NA**		
Concomitant medication use during study period (%)									$1.0x10^{-7}$
**Antibacterial use**	**450**	(59.9)	**52**	(49.5)	**136**	(59.9)	**262**	(62.5)	
**Antifungal use**	**72**	(9.6)	**9**	(8.6)	**26**	(11.5)	**37**	(8.8)	
**Antiviral use**	**53**	(7.1)	**<5**		**17**	(7.5)	**34**	(8.1)	
**Oral methotrexate use**	**332**	(44.2)	**10**	(9.5)	**92**	(40.5)	**230**	(54.9)	
**Any DMARD**	**493**	(65.6)	**12**	(11.4)	**139**	(61.2)	**342**	(81.6)	
**NSAID**	**491**	(65.4)	**75**	(71.4)	**151**	(66.5)	**265**	(63.2)	
**Gluco or corticosteroids**	**148**	(19.7)	**10**	(9.5)	**29**	(12.8)	**109**	(26.0)	
**Subcutaneous methotrexate use**	**14**	(1.9)	**NA**		**<5**		**11**	(2.6)	NA‡
**Comorbidities (%)§**									
Charlson score									$<2.2x10^{-16}$
0	**163**	(21.7)	**38**	(36.2)	**75**	(33)	**50**	(11.9)	
1	**190**	(25.3)	..		..		**167**	(39.9)	
2+	**34**	(4.5)	..		..		**28**	(6.7)	
unknown	**364**	(48.5)	**58**	(55.2)	**132**	(58.1)	**174**	(41.5)	

*Age at first delivery, † from 751 patients 22 did not have Scottish Index of Multiple Deprivation (SIMD) values. Follow-up is from first delivery to study end date. NA‡ Comparison was not calculable because numbers in some cells were not available, were zero and/or too small. § Following the statistical disclosure protocol of the Greater Glasgow and Clyde Safe Haven to avoid attribute disclosure, the cell suppression (primary and secondary) method was used for values in the Comorbidities section as some were too small . DMARD- disease-modifying anti-rheumatic drugs; NSAID- non-steroidal anti-inflammatory drug

For peer review only - http://bmjopen.bmj.com/site/about/guidelines.xhtml

Discontinuation and persistence

For all three conditions, approximately half of the patients were persistent on their index biologic throughout the study period (52% AS, 54% PsA, 48%RA). This was also observed in the incident cohort (55% AS, 55% PsA, 49%RA) as shown in Figure 2 for the overall cohort and Supplementary Figure 2.

Some patients after a period of discontinuation re-initiated the same biologic received at the index date (overall cohort 19% AS, 18% PsA and 21% RA; incident cohort 18%AS, 18% PsA, 19%RA).

A small proportion of patients switched. There were patients changing their index to another biologic either whilst being persistent (overall cohort 4% AS, 9% PsA and 5% RA; incident cohort 4% AS, 9% PsA and 6% RA) or re-initiating after a discontinuation, with a gap longer than 56 days between deliveries (overall cohort 7% AS, 4% PsA and 1% RA; incident cohort 5% AS, 3% PsA and 1% RA).

The remaining patients had a discontinuation and ceased treatment, meaning they did not restart a subcutaneous biologic during the study period (overall and incident cohort 18% AS, 15% PsA and 25% RA). A sensitivity analysis was conducted using a gap of 28 and 84 days showing similar trends (results for overall and incident cohorts are in Supplementary Figure 3).

Figure 2. Persistence and discontinuation (re-initiating or ceasing) of treatment with biologics according to rheumatic condition (n=751)

The crude survival curve of *time to discontinuation* (first gap greater than 56 days between biologic deliveries) of any biologic is shown in Figure 3. In the crude model, the proportion of patients persisting is slightly higher in PsA and AS compared to RA; however, there is no significant difference according to the log-rank test (p=0.0983). The same results were observed in the incident cohort (p=0.165; Supplementary Figure 4). The PH assumption was not violated with all the patients (global p = 0.757) or in the incident cohort (global p = 0.953).

Figure 3:Crude survival curves comparing persistent patients according to rheumatic condition. (n=655)

After adjustment for covariates, there was a slight difference between RA and PsA patients (HR =0.71, 95%CI:0.54-0.95, p=0.02). Lower risk of discontinuation was associated with the two least deprived quintiles (SIMD 4 or 5), whereas female sex and SIMD 3 (deprived quintile) were associated with a higher risk of discontinuation as shown in Figure 4. Age, SIMD 2 (more deprived quintile), prior use of of oral methotrexate, concomitant use of oral methotrexate or DMARD were not statistically signficant covariates and did not influence the model, which excluded 21 patients with missing SIMD values. A subanalysis was performed with the incident cohort in which there was no longer a difference between sex and SIMD4, however, the results of rheumatic conditions and SIMD 3 and SIMD5 quintiles

For peer review only - http://bmjopen.bmj.com/site/about/guidelines.xhtml

remained significant (see Supplementary Figure 5). As those who used the index biologic or switched was used as a stratifying variable, no effect size is estimated for this covariate. The PH assumption was not violated when estimating the adjusted model with all the patients (global p = 0.545) or the incident cohort (global p = 0.192).

Figure 4: Adjusted Hazard Ratios for persistence with biologics using Cox regression analysis excluding patients without SIMD values (n=634)

Adherence

Patients with at least two deliveries of the first (index) biologic (n = 671, 89.3%) were used to evaluate adherence, calculated using two methods.

Overall adherence during the period of treatment was over 80% when calculated using either the MRA or CR. However, this was consistently higher using %CR (median(IQR) for AS 96.6% (86.6-103.5), PsA 97% (85.5-103.2) and RA 96.6% (85.9-102.7) than using %MRA (median(IQR) for AS 84.0% (54.73-94.74), PsA 85.0% (50.76-98.25) and RA 82.4% (47.24-95.77)). The same pattern was also observed when comparing these two methods by the first biologic received (Figure 5A and 5B). Regardless of which of the two measurements were used, the few PsA patients (n=7) taking ustekinumab had higher adherence than was seen with any other biologic. Similar adherence results were obtained in the subset with incident users (Supplementary Figure 6).

Figure 5: Adherence to biologics by rheumatic condition according to %MRA (5A) and %CR (5B). MRA- medication refill adherence; CR- compliance rate.

DISCUSSION

This study is the first real-world cohort using data about SC biologic deliveries, combined with routine data from rheumatology clinics, community pharmacy dispensing, hospital discharges and mortality records to assess the utilisation of biologics in patients with RA, AS and PsA over a period of three years. This study found for all three conditions, approximately 50% of the patients were persistent on their index biologic and that overall adherence during the period of treatment was over 80%. Almost half of patients who discontinued their index biologic for a period greater than 56 days, ultimately re-initiate the same therapy with only a minority switching to another biologic. This highlights the importance of longitudinal data as many of these patients would have been incorrectly counted as cessations in other datasets.

A diverse range of studies have reported on the use of biologics over the last 20 years with the majority focused solely on RA,[19, 29, 43] and few studies comparing utilisation across the three main inflammatory rheumatologic conditions within a single healthcare system. Patient demographic characteristics in our study are similar to those reported in other epidemiological studies in the UK, Denmark, Norway and Germany,[11-16] despite the different methodologies used to create these cohorts.

Linked prescription and administrative health datasets have been used to study the utilisation of biologics in real-world settings in other studies in Europe, USA, Canada and Brazil.[24, 28, 29, 44, 45] However, to the best of our knowledge, this is the first study to use

For peer review only - http://bmjopen.bmj.com/site/about/guidelines.xhtml

routine data generated through "direct to home" deliveries of biologics as a source of information to assess how patients use these medicines. Most other routine administrative healthcare studies use prescriptions or claims/reimbursement data. In contrast, this homecare delivery service requires active participation of the patient in both ordering and then signing for receipt of the biologic, thereby confirming the biologic was prescribed, the prescription was processed and the patient received the biologic, providing better insight into how patients are using their medicines.

To understand the use of medicines in our cohort, we adopted the taxonomy proposed by ESPACOMP.[27] In summary, the framework enables us to quantify a patient's medicine taking behaviour in three respects: intensity, continuity and duration - how much of a drug does a patient take, how frequently, and for how long? *Persistence* and *discontinuation* allow us to study continuity and duration of treatment; while *adherence* enables us to study intensity of treatment in the context of the prescribed treatment regimen.

In those studies which examined persistence and discontinuation with biologics across rheumatic conditions, using a consistent in-study method over a comparable time period to our study, persistence was reported as being lower in RA than AS.[45, 46] This is similar to our findings where patients with RA were most likely to discontinue their index subcutaneous biologic, potentially reflecting the treat-to-target approach and greater therapeutic options available for RA at the time of this study. Our study also enabled quantification and categorisation of biologic switching, we believe for the first time, in both patients who were persistent regardless of changes in therapy and those who discontinued index therapy and then commenced a new biologic (Figure1).

In our Cox regression analyses, female sex was associated with lower persistence being consistent with several studies.[43, 45, 46] However, our findings regarding socieconomic status contradict those of the Machado *et al.* study as we have shown higher persistence in patients with lower deprivation levels. There are also discrepancies in the association with the use of methotrexate, as Heiberg *et al.* reported a strong association of its concomitant use with persistence in the three rheumatic diseases and we did not find an association. Randomised controlled trials of biologics in patients with PsA have failed to demonstrate superiority of combination therapies.[47, 48] Thus the role of methotrexate as a predictor for persistence remains unclear.

Persistence after the first, second or subsequent courses of biologics is still controversial. A French study found better persistence with first biologic compared to second or third,[49] with similar tendencies reported in Norway by Heiberg *et al.* In contrast, in a large UK national cohort of RA patients, those who switched to a second biologic had higher persistence[50] and the study using the RADIUS registry found similar persistence between first and second biologic.[51] In our study instead of comparing each course, we compared those persisting with their index biologic (first biologic) with those persisting after switching. Our findings were similar to the UK cohort as persistence was higher in those who had switched.

Various studies have examined adherence in a single rheumatoid condition, often RA[18-24, 26-29] but we could only find a single Italian study measuring adherence across RA, AS and PsA,[44] though applying a different method to our study. The challenge in comparing results between studies is often the absence of any detail on how adherence was measured and where specified, multiple measures being used. This has been highlighted in a recent

For peer review only - http://bmjopen.bmj.com/site/about/guidelines.xhtml

systematic review and meta-analysis by Scheiman-Elazary *et al*. of anti-arthritis medication in RA which reported adherence ranging from 10.5%-98.5% with the meta-analysis generating an adherence rate of 66% (95%CI 58-75%), noting that variability may result from different methods and readers should appraise studies according to the validity of the method, the scales and cut-off points.[18] From our experience of using both MRA and CR for adherence measurement we would recommend the use of CR as it is simple to calculate and includes the period between deliveries, thereby removing any ambiguity arising following the last supply of medicine.

In this study, there were cases where %MRA or %CR was above 100% suggesting some patients could have received more biologics than those expected for that period of time or adjustments with deliveries could be made later on which were not captured in the study period. The higher adherence to ustekinumab compared to the other biologics in this study may relate in part to the small numbers, a more recent introduction requiring the delivery of two 45mg vials to achieve the 90mg dose and a shorter use of this agent at the time of the study, although this observation has also been reported in psoriasis cohorts.[52, 53]

This current study has several strengths. This is the first real-world cohort containing type, frequency and dose of subcutaneous biologics delivered directly to patients for these three rheumatic diseases. The cohort is sustainable as it is generated as part of routine clinical care, avoiding the need for additional data-collection burden required in a traditional registry model, it can be easily updated and linked to other administrative datasets, has better coverage than individually-held clinical data and allows the use of biologics to be studied over time. The sustainability is a key factor, enabling biosimilar and new future innovator biologics to be easily captured as these reach the clinic. This model can also be easily extended to other specialities, including dermatology and gastroenterology, where similar biologic therapies are used. Recording the confirmed delivery of drug directly to patients may reduce some of the uncertainty and limitations associated with using prescription or reimbursement administrative datasets where it cannot be determined if patients actually received the drug. Recall bias was minimized by linking routine data and not based on self-reported persistence or adherence, while selection bias was addressed by including all the patients receiving biologics in the largest Scottish Health Board.

This study also has some limitations. The cohort covers a specific, albeit highly populated, region in Scotland and the generalisability of the results has to be tested. It is a mixed cohort of naive and existing biologic users, which could influence the assessment of medication utilisation. However, the results were similar in the overall and incident cohorts. By using CR as an adherence measurement patients discontinuing prior to study completion are not considered.This was overcome in our study by reporting together adherence, persistence and discontinuation.The reasons for discontinuation or treatment interruption are not currently captured in HSD, so it is of limited utility in terms of understanding the factors that influence the use of biologics. History of biologics or subcutaneous methotrexate being used prior to 2012 was not available. However, during the study period the use subcutaneous methotrexate was captured within the HSD, recording a relatively low use. The biologics home delivery service only captures subcutaneous biologics and not intravenous agents, such as infliximab or rituximab, so will have missed where patients were switched to these agents. However, the latter are far smaller in volume and may be captured from rheumatology clinic documentation and hospital administrative data where attendance for infusion guarantees receipt of drug.

For peer review only - http://bmjopen.bmj.com/site/about/guidelines.xhtml

CONCLUSION

The long-term evaluation of biologics for the treatment of rheumatic and related diseases is a desirable goal on a clinical level, but it is also a requirement from a pharmacological and economic point of view. Traditional drug or disease registers are expensive, resource intensive and difficult to sustain, so strategies using linked administrative and routine clinical data provide the best opportunity to capture the true picture of biologic use. Additionally a homecare delivery database may reduce some of the uncertainties associated with other administrative databases.

In moving forward, we strongly advocate that future studies should consider application of the ESPACOMP framework[25] to better understand the use of biologics i.e. the combination of discontinuation, persistence and adherence metrics thus leading to a more consistent approach which enables more comparability between studies.

Acknowledgements: Farr Institute @ Scotland, GGC Safe Haven team, Norman Lannigan and Kevin Milne for providing the Homecare Database and allow its use for research purposes. FAA acknowledges the support from FAPEMIG, the Minas Gerais State Research Foundation, Brazil.

REFERENCES

1. Coates LC, Kavanaugh A, Mease PJ, *et al*. Group for Research and Assessment of Psoriasis and Psoriatic Arthritis 2015 Treatment Recommendations for Psoriatic Arthritis. *Arthritis & rheumatology (Hoboken, NJ)* 2016; **68**(5): 1060-71.
2. Gossec L, Smolen JS. European League Against Rheumatism (EULAR) recommendations for the management of psoriatic arthritis with pharmacological therapies: 2015 update. *Ann Rheum Dis* 2016; **75**(3): 499-510.
3. NICE. Rheumatoid arthritis in adults: management. Clinical guideline CG79. England: National Institute for Health and Care Excellence; 2015.
4. Smolen JS, Landewe R, Bijlsma J, *et al*. EULAR recommendations for the management of rheumatoid arthritis with synthetic and biological disease-modifying antirheumatic drugs: 2016 update. *Ann Rheum Dis* 2017; **76**(6): 960-77.
5. van der Heijde D, Ramiro S. 2016 update of the ASAS-EULAR management recommendations for axial spondyloarthritis. *Ann Rheum Dis* 2017; **76**(6): 978-91.
6. Macfarlane GJ, Barnish MS, Jones EA, *et al*. The British Society for Rheumatology Biologics Registers in Ankylosing Spondylitis (BSRBR-AS) study: Protocol for a prospective cohort study of the long-term safety and quality of life outcomes of biologic treatment. *BMC Musculoskelet disorders* 2015; **16**: 347.
7. Nam JL, Takase-Minegishi K, Ramiro S. Efficacy of biological disease-modifying antirheumatic drugs: a systematic literature review informing the 2016 update of the EULAR recommendations for the management of rheumatoid arthritis. *Ann Rheum Dis* 2017; **76**(6): 1113-36.
8. Nikiphorou E, Buch MH, Hyrich KL. Biologics registers in RA: methodological aspects, current role and future applications. *Nat Rev Rheumatol* 2017; **13**(8): 503-10.
9. Saad AA, Ashcroft DM, Watson KD, *et al*. Efficacy and safety of anti-TNF therapies in psoriatic arthritis: an observational study from the British Society for Rheumatology Biologics Register. *Rheumatology (Oxford, England)* 2010; **49**(4): 697-705.
10. Simard JF, Arkema EV, Sundstrom A, *et al*. Ten years with biologics: to whom do data on effectiveness and safety apply? *Rheumatology (Oxford, England)* 2011; **50**(1): 204-13.
11. Dean LE, Jones GT, MacDonald AG, *et al*. Global prevalence of ankylosing spondylitis. *Rheumatology* 2014; **53**(4): 650-7.

For peer review only - http://bmjopen.bmj.com/site/about/guidelines.xhtml

12. Egeberg A, Kristensen LE, Thyssen JP, *et al*. Incidence and prevalence of psoriatic arthritis in Denmark: a nationwide register linkage study. *Ann Rheum Dis* 2017; **8**(210963): 2016-210963.
13. Hoff M, Gulati AM, Romundstad PR, *et al*. Prevalence and incidence rates of psoriatic arthritis in central Norway: data from the Nord-Trondelag health study (HUNT). *Ann Rheum Dis* 2015; **74**(1): 60-4.
14. Humphreys JH, Verstappen SM, Hyrich KL, *et al*. The incidence of rheumatoid arthritis in the UK: comparisons using the 2010 ACR/EULAR classification criteria and the 1987 ACR classification criteria. Results from the Norfolk Arthritis Register. *Ann Rheum Dis* 2013; **72**(8): 1315-20.
15. Huscher D, Thiele K, Rudwaleit M, *et al*. Trends in treatment and outcomes of ankylosing spondylitis in outpatient rheumatological care in Germany between 2000 and 2012. *RMD Open* 2015; **1**(1): 2014-000033.
16. Ogdie A, Weiss P. The Epidemiology of Psoriatic Arthritis. *RheumDis Clin North Am* 2015; **41**(4): 545-68.
17. Blum MA, Koo D, Doshi JA. Measurement and rates of persistence with and adherence to biologics for rheumatoid arthritis: a systematic review. *Clin Ther* 2011; **33**(7): 901-13.
18. Scheiman-Elazary A, Duan L, Shourt C, *et al*. The Rate of Adherence to Antiarthritis Medications and Associated Factors among Patients with Rheumatoid Arthritis: A Systematic Literature Review and Metaanalysis. *J Rheumatol* 2016; **43**(3): 512-23.
19. Vangeli E, Bakhshi S, Baker A, *et al*. A Systematic Review of Factors Associated with Non-Adherence to Treatment for Immune-Mediated Inflammatory Diseases. *Adv Ther* 2015; **32**(11): 983-1028.
20. Bhoi P, Bessette L, Bell MJ, *et al*. Adherence and dosing interval of subcutaneous antitumour necrosis factor biologics among patients with inflammatory arthritis: analysis from a Canadian administrative database. *BMJ open* 2017; **7**(9): e015872.
21. Ebina K, Hashimoto M, Yamamoto W, *et al*. Drug retention and discontinuation reasons between seven biologics in patients with rheumatoid arthritis -The ANSWER cohort study. *PloS one* 2018; **13**(3): e0194130.
22. Glintborg B, Ostergaard M, Krogh NS, *et al*. Clinical response, drug survival and predictors thereof in 432 ankylosing spondylitis patients after switching tumour necrosis factor alpha inhibitor therapy: results from the Danish nationwide DANBIO registry. *Ann Rheum Dis* 2013; **72**(7): 1149-55.
23. Kruger K, Burmester GR, Wassenberg S,*et al*. Effectiveness and safety of golimumab in patients with rheumatoid arthritis, psoriatic arthritis and ankylosing spondylitis under real-life clinical conditions: non-interventional GO-NICE study in Germany. *BMJ open* 2018; **8**(6): e021082.
24. Lyu R, Govoni M, Ding Q, *et al*. Treatment persistence among patients with rheumatoid disease (RA, AS, PsA) treated with subcutaneous biologics in Germany. *Rheumatol Int* 2015; **28**: 28.
25. Oei HB, Hooker RS, Cipher DJ,*et al*. High rates of stopping or switching biological medications in veterans with rheumatoid arthritis. *Clin Exp Rheumatol* 2009; **27**(6): 926-34.
26. Wallman JK, Kapetanovic MC, Petersson IF *et al*. Comparison of non-radiographic axial spondyloarthritis and ankylosing spondylitis patients--baseline characteristics, treatment adherence, and development of clinical variables during three years of anti-TNF therapy in clinical practice. *Arthritis Res Ther* 2015; **17**(378): 015-0897.
27. Vrijens B, De Geest S, Hughes DA, et al. A new taxonomy for describing and defining adherence to medications. *Br J Clin Pharmacol* 2012; **73**(5): 691-705.
28. Acurcio FA, Machado MA, Moura CS, *et al*. Medication Persistence of Disease-Modifying Antirheumatic Drugs and Anti-Tumor Necrosis Factor Agents in a Cohort of Patients With Rheumatoid Arthritis in Brazil. *Arthritis Care Res* 2016; **68**(10): 1489-96.
29. Fisher A, Bassett K, Wright JM,*et al*. Comparative persistence of the TNF antagonists in rheumatoid arthritis--a population-based cohort study. *PloS one* 2014; **9**(8): e105193.
30. Ogale S, Hitraya E, Henk HJ. Patterns of biologic agent utilization among patients with rheumatoid arthritis: a retrospective cohort study. *BMC Musculoskelet Disord* 2011; **12**: 204.

For peer review only - http://bmjopen.bmj.com/site/about/guidelines.xhtml

31. Wadstrom H, Eriksson JK, Neovius M, *et al*. How good is the coverage and how accurate are exposure data in the Swedish Biologics Register (ARTIS)? *Scand J Rheumatol* 2015; **44**(1): 22-8.
32. ISD Scotland. ISD Scotland data dictionary. Edinburgh; 2016. http://www.ndc.scot.nhs.uk/Dictionary-A-Z/Definitions/index.asp?Search=C&ID=128&Title=CHI%20Number (accessed 10 December 2017)
33. NRS. Mid-year population estimates. Edinburgh; 2016. http://www. nrscotland.gov.uk/statistics-and-data/statistics/statistics-by-theme/ population/population-estimates/mid-year-population-estimates (accessed 10 December 2017)
34. Alvarez-Madrazo S, McTaggart S, Nangle C,*et al*. Data Resource Profile: The Scottish National Prescribing Information System (PIS). *Int J Epidemiol* 2016; **45**(3): 714-5f.
35. ISD Scotland. National Data Catalogue: National Datasets. Edinburgh; 2017. http://www.ndc.scot.nhs.uk/National-Datasets/ (accessed 10 December 2017)
36. Scottish Medicines Consortium . https://www.scottishmedicines.org.uk/(accessed 22 June 2017)
37. NHS National Services Scotland. Statistical Disclosure Control Protocol. Version 3.0. 2015. https://www.isdscotland.org/About-ISD/Confidentiality/disclosure_protocol_v3.pdf (accessed 30 January 2019)
38. Mueller T, Alvarez-Madrazo S, Robertson C, *et al*.. Use of direct oral anticoagulants in patients with atrial fibrillation in Scotland: Applying a coherent framework to drug utilisation studies. *Pharmacoepidemiol Drug Saf* 2017; **26**(11): 1378-86.
39. Gregoire JM, J. Assessment of adhrence to drug treatment in database records. In: Elseviers M, Wettermak B, Almarsdotti AB.; *et al*., ed. Drug Utilization Research: Methods and Applications. Chichester: Wiley Blackwell; 2016: 369-80.
40. Grambsch PM and Therneau TM. Proportional hazards tests and diagnostics based on weighted residuals. *Biometrika* 1994; **81**: 515-26.
41. Hess LM, Raebel MA, Conner DA, *et al*. Measurement of adherence in pharmacy administrative databases: a proposal for standard definitions and preferred measures. *Ann Pharmacother* 2006; **40**(7-8): 1280-88.
42. R Core Team (2018): A language and environment for statistical computing. R Foundation for Statistical Computing, Vienna, Austria. 2018. http://www.R-project.org/.

43. Souto A, Maneiro JR, Gomez-Reino JJ. Rate of discontinuation and drug survival of biologic therapies in rheumatoid arthritis: a systematic review and meta-analysis of drug registries and health care databases. *Rheumatology (Oxford, England)* 2016; **55**(3): 523-34.

44. Degli Esposti L, Sangiorgi D, Perrone V, et al. Adherence and resource use among patients treated with biologic drugs: findings from BEETLE study. *Clinicoecon Outcomes Res: CEOR* 2014; **6**: 401-7.
45. Machado MA, Moura CS, Ferre F, *et al*. Treatment persistence in patients with rheumatoid arthritis and ankylosing spondylitis. *Rev Saude Publica* 2016; **50**(50): 2016050006265.
46. Heiberg MS, Koldingsnes W, Mikkelsen K, *et al*. The comparative one-year performance of anti-tumor necrosis factor alpha drugs in patients with rheumatoid arthritis, psoriatic arthritis, and ankylosing spondylitis: results from a longitudinal, observational, multicenter study. *Arthritis Rheum* 2008; **59**(2): 234-40.
47. Mease PJ, Gladman DD, Ritchlin CT, *et al*. Adalimumab for the treatment of patients with moderately to severely active psoriatic arthritis: results of a double-blind, randomized, placebo-controlled trial. *Arthritis Rheum* 2005; **52**(10): 3279-89.
48. Mease PJ, Kivitz AJ, Burch FX, et al. Etanercept treatment of psoriatic arthritis: safety, efficacy, and effect on disease progression. *Arthritis Rheum* 2004; **50**(7): 2264-72.
49. Duclos M, Gossec L, Ruyssen-Witrand A, *et al*. Retention rates of tumor necrosis factor blockers in daily practice in 770 rheumatic patients. *J Rheumatol* 2006; **33**(12): 2433-8.

For peer review only - http://bmjopen.bmj.com/site/about/guidelines.xhtml

50. Hyrich KL, Lunt M, Watson KD, *et al*. Outcomes after switching from one anti-tumor necrosis factor alpha agent to a second anti-tumor necrosis factor alpha agent in patients with rheumatoid arthritis: results from a large UK national cohort study. *Arthritis Rheum* 2007; **56**(1): 13-20.
51. Markenson JA, Gibofsky A, Palmer WR, *et al*. Persistence with anti-tumor necrosis factor therapies in patients with rheumatoid arthritis: observations from the RADIUS registry. *J Rheumatol* 2011; **38**(7): 1273-81.
52. Menter A, Papp KA, Gooderham M, *et al*. Drug survival of biologic therapy in a large, disease-based registry of patients with psoriasis: results from the Psoriasis Longitudinal Assessment and Registry (PSOLAR). *J Eur Acad Dermatol Venereol: JEADV* 2016; **30**(7): 1148-58.
53. Zweegers J, van den Reek JM, van de Kerkhof PC, *et al*. Body mass index predicts discontinuation due to ineffectiveness and female sex predicts discontinuation due to side-effects in patients with psoriasis treated with adalimumab, etanercept or ustekinumab in daily practice: a prospective, comparative, long-term drug-survival study from the BioCAPTURE registry. *Br J Dermatol* 2016; **175**(2): 340-7.

FOOTNOTES

Contributors: All authors contributed to study design. SAM extracted, prepared the data and performed the analyses. SAM drafted the manuscript, with revisions by KK, SS, YS,BG,AMA,FAA and MB.

Funding: We acknowledge the support from The Farr Institute @ Scotland. The Farr Institute @ Scotland is supported by a 10-funder consortium: Arthritis Research UK, the British Heart Foundation, Cancer Research UK, the Economic and Social Research Council, the Engineering and Physical Sciences Research Council, the Medical Research Council, the National Institute of Health Research, the National Institute for Social Care and Health Research (Welsh Assembly Government), the Chief Scientist Office (Scottish Government Health Directorates), the Wellcome Trust, (MRC Grant No: MR/K007017/1).

Competing interest: SS has received honoraria from Janssen, Novartis, AbbVie, Pfizer, UCB, Celgene and Boehringer-Ingelheim; and research funding from UCB, Celgene, Boehringer-Ingelheim, BMS and Pfizer.

Patient consent: Not required.

Ethical approval: Ethics permission was not required as only non-identifiable routine data was used for this study. The study was approved by the NHS GGC Safe Haven Local Privacy Advisory Committee (Project number: GSH/15/MT/001). No patient identifiers were available to the study team and all data were accessed via the GGC Safe Haven.

Provenance and peer review: not commissioned; externally peer reviewed.

Data sharing statement: All data supporting this research are openly available to researchers based in recognized UK institutions from http://www.nhsggc.org.uk/about-us/professional-support-sites/nhsggc-safe-haven/your-research/available-datasets. For further information contact the Greater Glasgow and Clyde Safe Haven by emailing safehaven@ggc.scot.nhs.uk

For peer review only

For peer review only - http://bmjopen.bmj.com/site/about/guidelines.xhtml

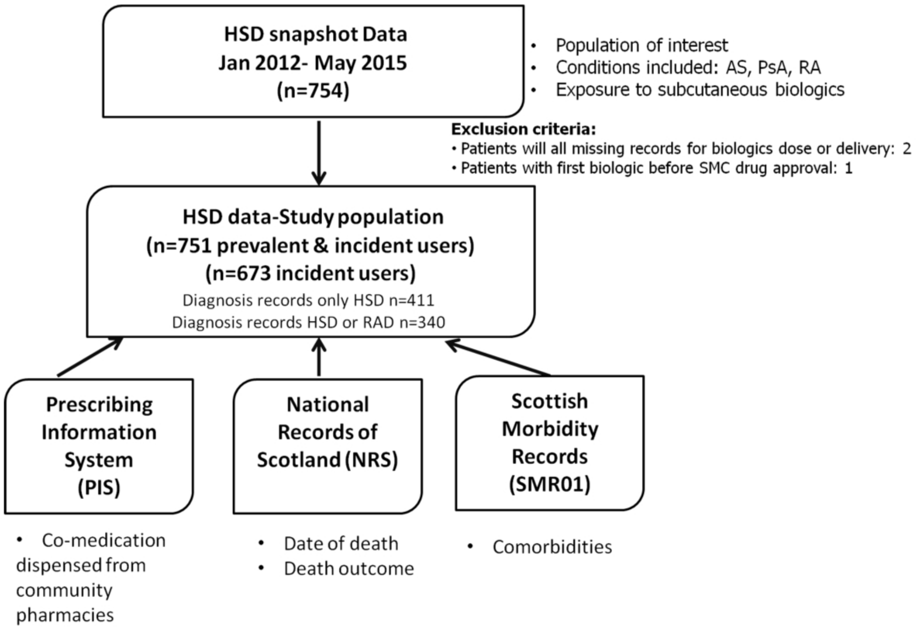

Figure 1. Variables obtained from different Scottish datasets.

306x192mm (300 x 300 DPI)

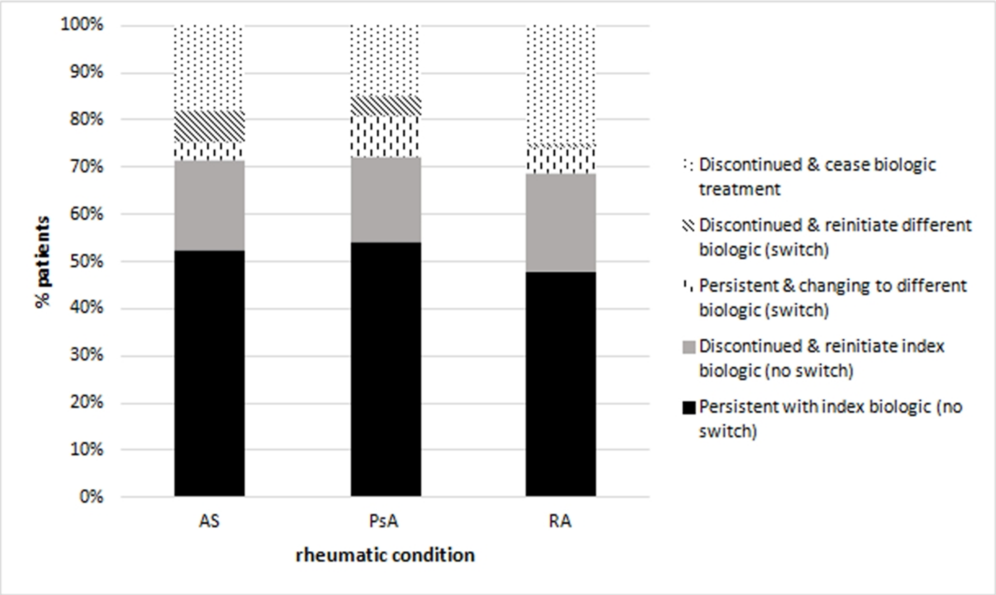

215x129mm (300 x 300 DPI)

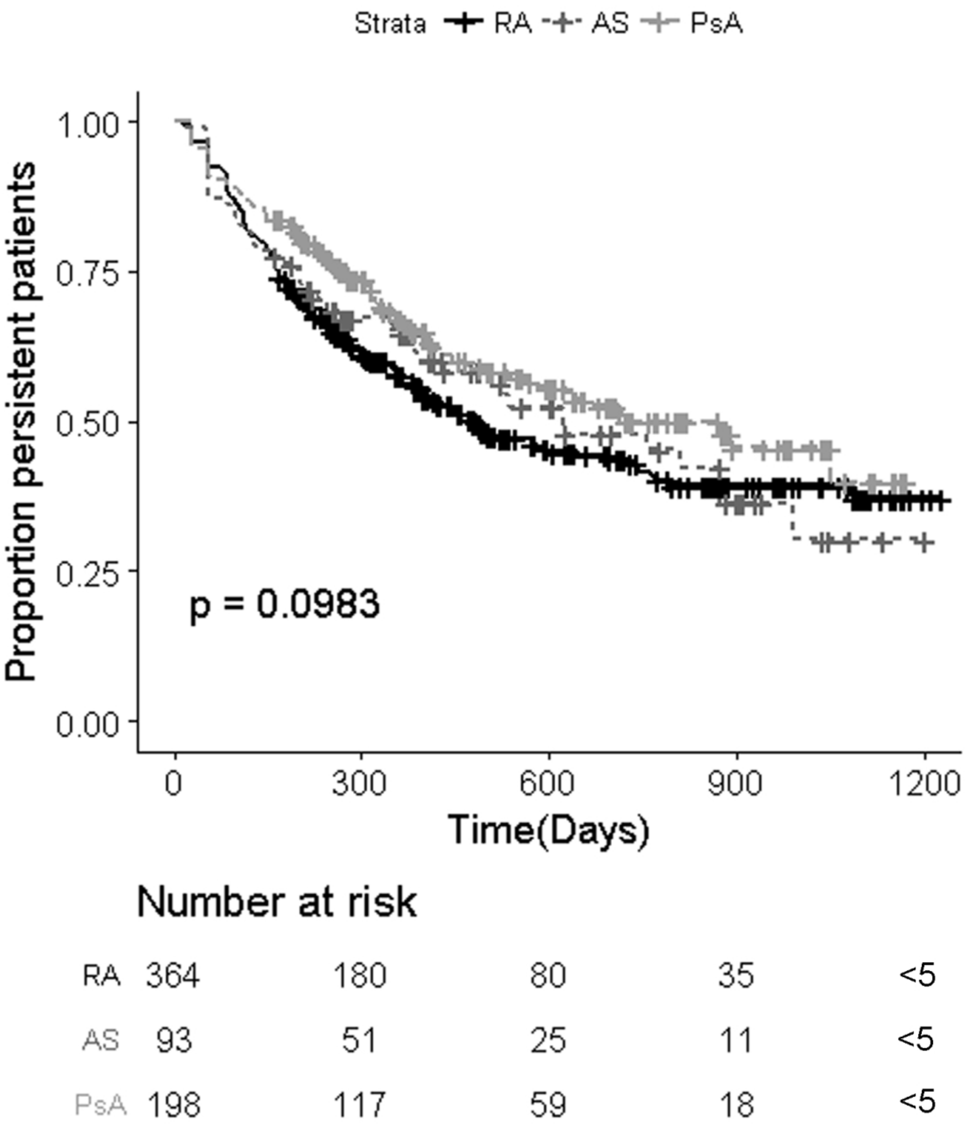

Figure 3:Crude survival curves comparing persistent patients according to rheumatic condition. (n=655)

125x152mm (300 x 300 DPI)

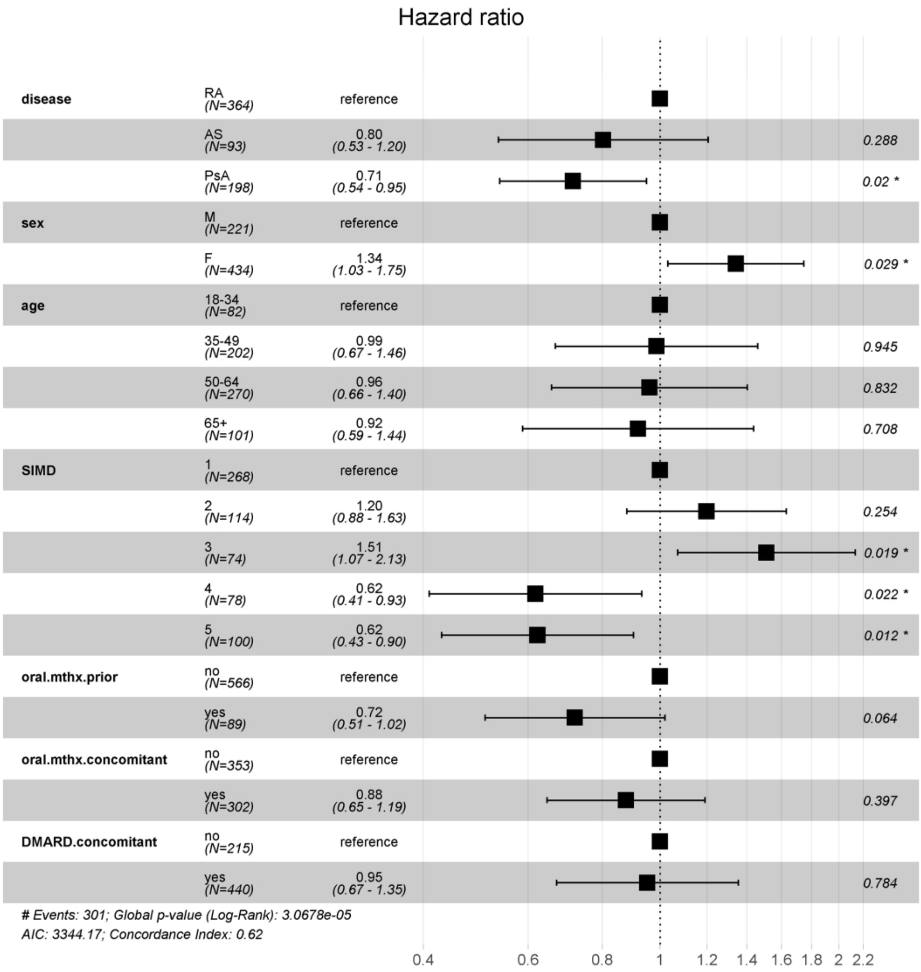

Caption : Figure 4: Adjusted Hazard Ratios for persistence with biologics using Cox regression analysis excluding patients without SIMD values (n=634)

199x199mm (300 x 300 DPI)

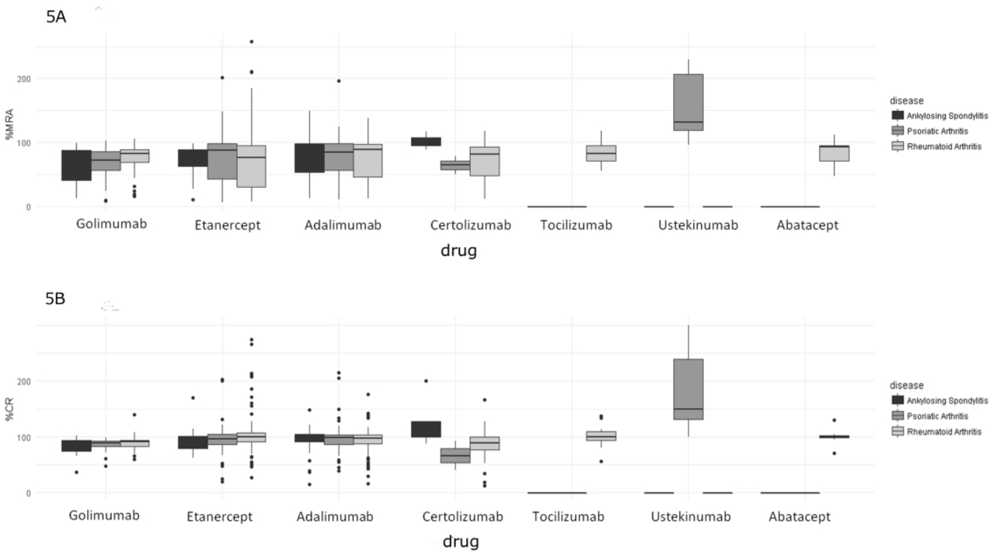

Figure 5: Adherence to biologics by rheumatic condition according to %MRA (5A) and %CR (5B). MRA- medication refill adherence; CR- compliance rate.

Supplementary file :

Validation method and results

Of the 751 patients in HSD, 340 were also in the RAD. A minority of these (n=48) had diagnosis recorded in either of the datasets with the majority of these (n=292) with diagnosis recorded in both datasets (supplementary figure 1). RAD diagnosis was considered as the "gold standard" because it was collected in rheumatology clinics by clinical staff and was used to determine the validity of HSD diagnosis in the 292 patients. There were 3540 patients diagnosed with the three rheumatic conditions (RA, PsA or AS) who attended rheumatology clinics in Glasgow and were only recorded in RAD. These patients were not used for the validation study.

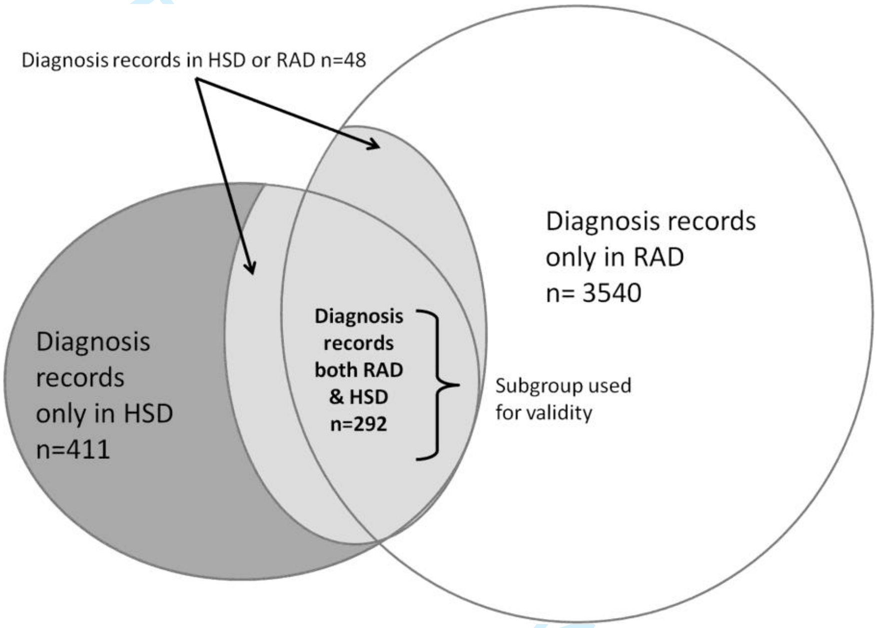

Supplementary Figure 1. Diagnoses of rheumatic conditions according to HSD and RAD records.

Validity of diagnoses of rheumatic conditions (PPV and sensitivity)

The positive predictive value (PPV) and sensitivity with 95% confidence intervals were calculated for the diagnosis of RA, PsA or AS in HSD. PPV was calculated as the proportion of subjects in HSD with an indication for any of these three conditions in first recorded delivery and confirmed diagnosis in RAD. Sensitivity was calculated as the proportion of RAD cases who had a RA, PsA or AS diagnosis from all the RA, PsA or AS patients in the 292 subjects. A high PPV identifies a true population, or this case subgroup, of patients with chronic inflammatory diseases; while a high sensitivity allows to asses as many cases as possible.

Validity of arthritic conditions

From the subset of patients with arthritic conditions in HSD, the diagnosis was confirmed in 292 patients, with a 91.8%(95%CI: 86.4-95.1) PPV and a sensitivity of 97.3% (95%CI:93.3-99.0) for RA, 97.4% (95%CI:86.5-99.9) PPV and 94.9% (95%CI: 83.1-98.6)sensitivity for AS and 95.8% (95%CI:89.8-98.4) PPV and 88.5%(95%CI:80.9-93.3) sensitivity for PsA.

For peer review only - http://bmjopen.bmj.com/site/about/guidelines.xhtml

Many studies focus in the optimal clinical use and safety of medication in rheumatic diseases using administrative or claims data and one of the first requirements, following the best-practice consensus from the CANRAD Network, is the validation of case definitions.[1] There are mainly two types of validation methods (a) internal using algorithms or sensitivity analyses within the data or (b) external using data from other sources such as clinical notes or data captured by general practitioners.[2] In this study, an external validation was performed by comparing the diagnoses of the patients present in both HSD and RAD databases. Both the PPV and sensitivity were above 80% for the three diseases. In contrast to several studies, instead of using ICD-10 codes, our study used free text entered by clinicians in RAD which was compared to the diagnoses entered from the prescriptions in HSD. All the patients also had at least one prescription for a subcutaneous biologic in HSD. Our sample size (n=292) is in the range of similar studies aimed to validate diagnoses (n=85-450), and our PPV values and sensitivities are comparable to others using data from rheumatology clinics (PPV 95-97%; sensitivities reported 30-100%).[3, 4]

Analyses with incident users of subcutaneous biologics

Incident users were defined as patients who did not receive a subcutaneous biologic in the first six months of the study period (from January-June 2012). This incident cohort included 673 patients (89.6% from the original cohort), from them 95 had AS, 208 PsA and 370 RA (Supplementary Table 1). The proportion of patients according to age, gender and the distribution of socioeconomic levels (SIMD) were similar.

A total of 88.9% of incident patients had more than one delivery of biologic during the study period (compared to 89.3% from the original cohort) and the same percentage of 83.2% did not switch biologic.

The index biologics mostly used were also adalimumab and etanercept. Although the proportion of users according to their index biologics did not change, there were less users of adalimumab, certolizumab pegol and etanercept in the incident cohort as the prevalent users of these three medications were excluded. This is in agreement with the dates of approval for their use as these biologics were the first ones approved and patients were more likely to be users before 2012.

The pattern of concomitant medications prevailed in the incident cohort as RA and PsA patients were frequent users of oral methotrexate, DMARDs and NSAIDs, while AS patients mainly used NSAIDs.

For peer review only - http://bmjopen.bmj.com/site/about/guidelines.xhtml

Supplementary Table 1. Baseline characteristics of incident patients with rheumatic conditions receiving subcutaneous biologic from July 2012-May 2015 (n=673)

Rheumatic disease	All		Ankylosing spondylitis		Psoriatic arthritis		Rheumatoid arthritis		p-value
n	**673**		**95**		**208**		**370**		
**Female - n (%)**	**446**	(66.3)	**34**	(36.8)	**117**	(56.3)	**295**	(79.7)	$<2.2\times10^{-16}$
**Age***									
Age (median, IQR)*	**52.9(42.5-60.4)**		**47.4 (35.6-55.4)**		**48.0(40.1-57.3)**		**55.9(47.1-64.2)**		1.78×10^{-11}
**Age by category**									4.6×10^{-10}
**18-34 (%)**	**87**	(12.9)	**21**	(22.1)	**34**	(16.3)	**32**	(8.6)	
**35-49 (%)**	**196**	(29.1)	**31**	(32.6)	**83**	(39.9)	**82**	(22.2)	
**50-64 (%)**	**282**	(41.9)	**35**	(36.8)	**75**	(36.1)	**172**	(46.5)	
**65+ (%)**	**108**	(16.0)	**8**	(8.4)	**16**	(7.7)	**84**	(22.7)	
SIMD †									0.314
**1 Most deprived (%)**	**274**	(40.7)	**38**	(43.2)	**85**	(42.1)	**151**	(41.8)	
**2 (%)**	**117**	(17.4)	**19**	(21.6)	**40**	(19.8)	**58**	(16.1)	
**3 (%)**	**75**	(11.1)	**13**	(14.8)	**22**	(10.9)	**40**	(11.1)	
**4 (%)**	**78**	(11.6)	**12**	(13.6)	**22**	(10.9)	**44**	(12.2)	
**5 Least deprived (%)**	**107**	(15.9)	**6**	(6.8)	**33**	(16.3)	**68**	(18.8)	
Number of deliveries of biologics									
Number of deliveries of biologics (median, IQR)	**6**	(3-10)	**6**	(3-11)	**6**	(3-11)	**5**	(3-10)	0.400

For peer review only - http://bmjopen.bmj.com/site/about/guidelines.xhtml

Number of deliveries of biologics by category (%)									0.803
1	**75**	(11.1)	**13**	(13.7)	**18**	(8.7)	**44**	(11.9)	
2-10	**434**	(64.5)	**57**	(60.0)	**136**	(65.4)	**241**	(65.1)	
11-20	**129**	(19.2)	**19**	(20.0)	**43**	(20.7)	**67**	(18.1)	
21+	**35**	(5.2)	**6**	(6.3)	**11**	(5.3)	**18**	(4.9)	
Number of different biologics delivered (%)									
1	**560**	(83.2)	**81**	(85.3)	**165**	(79.3)	**314**	(84.9)	0.196
2, 3 or 4 (switchers)	**113**	(16.8)	**14**	(14.7)	**43**	(20.7)	**56**	(15.1)	
First biologic delivered(%)									
									$7.33x10^{-11}$
**Adalimumab**	**263**	(39.1)	**47**	(49.5)	**98**	(47.1)	**118**	(31.9)	
**Certolizumab Pegol**	**68**	(10.1)	**6**	(6.3)	**<5**		**60**	(16.2)	
**Etanercept**	**215**	(31.9)	**22**	(23.2)	**77**	(37.0)	**116**	(31.4)	
**Golimumab**	**91**	(13.5)	**20**	(21.1)	**24**	(11.5)	**47**	(12.7)	
**Abatacept**	**8**	(1.2)	**NA**		**NA**		**8**	(2.2)	NA‡
**Tocilizumab**	**21**	(3.1)	**NA**		**NA**		**21**	(5.7)	
**Ustekinumab**	**7**	(1.0)	**NA**		**7**	(3.4)	**NA**		
**Follow-up (days), *(median, IQR)***	**480**	**(264.0-734.0)**	**442 (278-749.5)**		**504.5(267.5-763.5)**		**482.0 (263.3-731.5)**		0.950

Concomitant medication use within one year before index date (%)									
**Antibacterial use**	**20**	(3.0)	**<5**		**<5**		**14**	(3.8)	0.149
**Oral methotrexate use**	**39**	(5.8)	**<5**		**9**	(4.3)	**29**	(7.8)	
**Any DMARD**	**54**	(8.0)	**<5**		**13**	(6.3)	**40**	(10.8)	
**NSAID**	**54**	(8.0)	**8**	(8.4)	**15**	(7.2)	**31**	(8.4)	
**Antifungal use**	**<5**		**NA**		**NA**		**<5**		NA[‡]
**Antiviral use**	**NA**		**NA**		**NA**		**<5**		
**Subcutaneous methotrexate use**	**NA**		**NA**		**NA**		**NA**		
**Gluco or corticosteroids**	**10**	(2.5)	**NA**		**<5**		**9**	(2.4)	
Concomitant medication use during study period (%)									1.0×10^{-7}
**Antibacterial use**	**394**	(58.5)	**46**	(48.4)	**124**	(59.6)	**224**	(60.5)	
**Antifungal use**	**62**	(9.2)	**7**	(7.4)	**22**	(10.6)	**33**	(8.9)	
**Antiviral use**	**44**	(6.5)	**<5**		**16**	(7.7)	**26**	(7.0)	
**Oral methotrexate use**	**284**	(42.2)	**7**	(7.4)	**78**	(37.5)	**199**	(53.8)	
**Any DMARD**	**432**	(64.2)	**9**	(9.5)	**122**	(58.7)	**301**	(81.4)	
**NSAID**	**430**	(63.9)	**65**	(68.4)	**137**	(65.9)	**228**	(61.6)	
**Gluco or corticosteroids**	**132**	(19.6)	**10**	(10.5)	**26**	(12.5)	**96**	(25.9)	
**Subcutaneous methotrexate use**	**10**	(1.5)	**NA**		**<5**		**7**	(1.9)	NA[‡]

**Comorbidities (%)**[§]									$<2.2\times10^{-16}$
Charlson score									
0	**145**	(21.5)	**32**	(30.5)	**66**	(31.7)	**47**	(12.7)	
1	**164**	(24.4)	..		..		**143**	(38.6)	
2+	**33**	(4.9)	..		..		**27**	(7.3)	
unknown	**331**	(49.2)	**55**	(52.4)	**123**	(59.1)	**153**	(41.4)	

*Age at first delivery, † from 673 patients 22 did not have Scottish Index of Multiple Deprivation (SIMD) values. Follow-up is from first delivery to study end date. NA[‡] Comparison was not calculable because numbers in some cells were not available, were zero and/or too small. § Following the statistical disclosure protocol of the Greater Glasgow and Clyde Safe Haven to avoid attribute disclosure, the cell suppression (primary and secondary) method was used for for values in the Comorbidities section as some were too small. DMARD- disease-modifying anti-rheumatic drugs; NSAID- non-steroidal anti-inflammatory drug

For peer review only - http://bmjopen.bmj.com/site/about/guidelines.xhtml

Discontinuation and persistence

Also in the incident cohort approximately half of the patients were persistent on their index biologic throughout the study period (55% AS, 55% PsA, 49%RA) as shown in Supplementary Figure 2. From those who discontinue treatment the majority reintiate it with their index biologic (18%AS, 18% PsA, 19%RA) and a few switch to other biologics (5%AS, 3% PsA, 1%RA).

Supplementary Figure 2. Persistence and discontinuation (re-initiating or ceasing) of treatment with biologics according to rheumatic condition in the incident cohort (n=673)

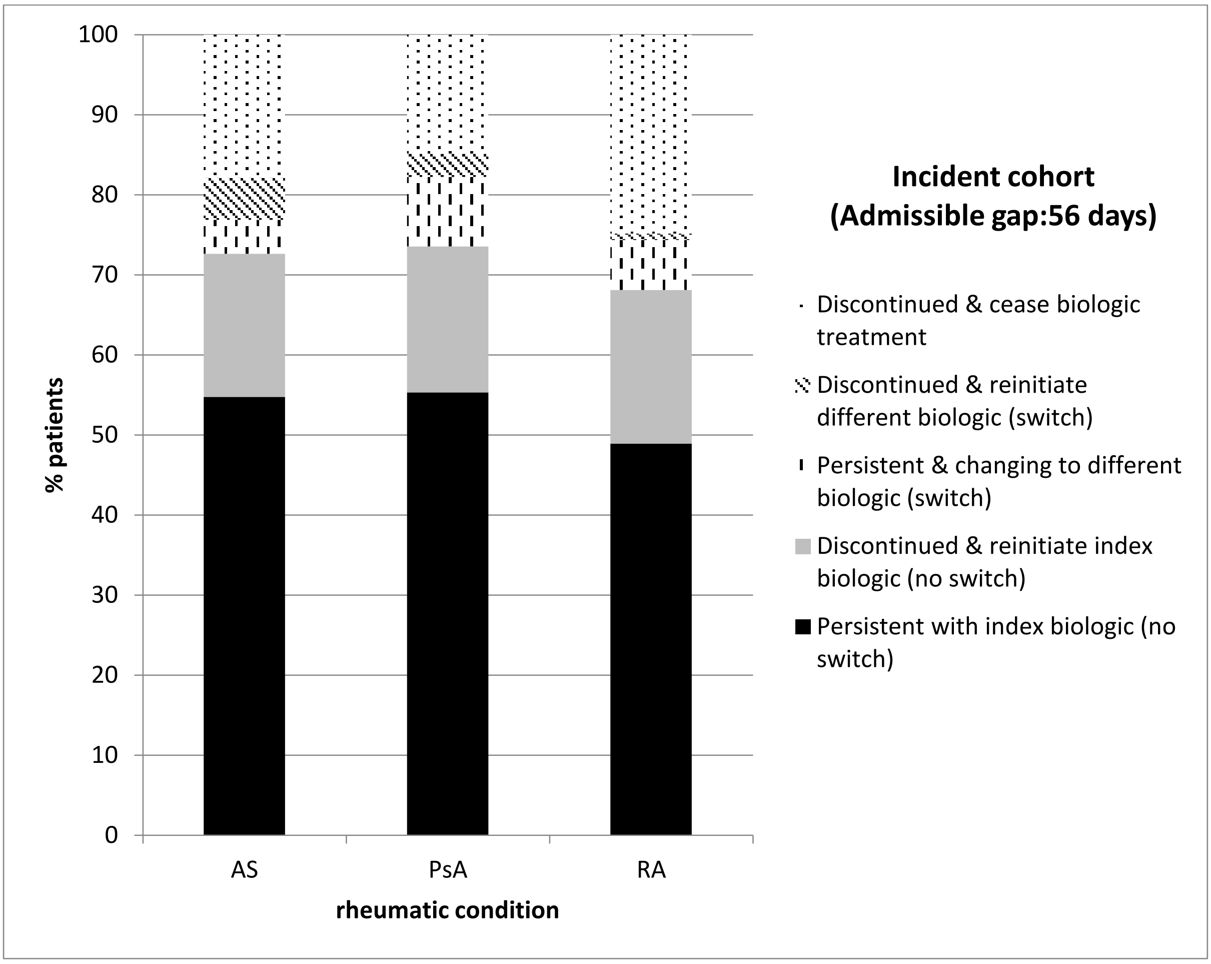

Sensitivity analyses discontinuation and persistence

Supplementary Figure 3. Persistence and discontinuation (re-initiating or ceasing) of treatment with biologics according to rheumatic condition in all the cohort with a gap of 28 days (Fig.6A) or 84 days (Fig.6B) and in the incident cohort with a gap of 28 days (Fig.6C) or 84 days (Fig.6D)

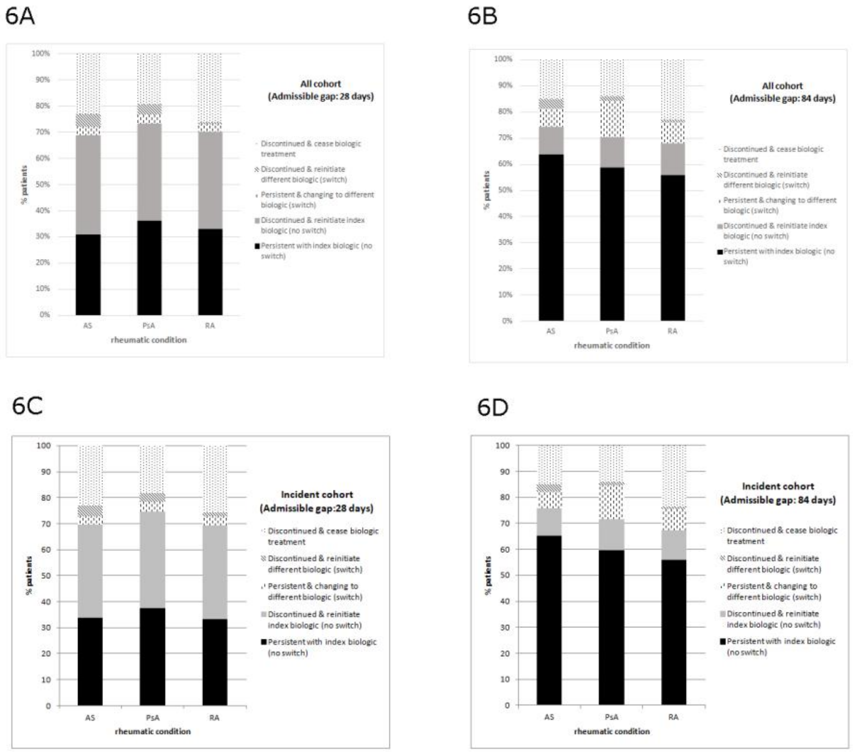

only

The crude survival curve of *time to discontinuation* (first gap greater than 56 days between biologic deliveries) of any biologic in the incident is shown in Supplementary Figure 4. In the crude model, the proportion of patients persisting is also slightly higher in PsA and AS compared to RA; however, there is no significant difference according to the log-rank test (p=0.165) and the Cox regression between RA and PsA (HR =0.77, 95%CI:0.58-1.01, p=0.0597).

Supplementary Figure 4:Crude survival curves comparing persistent patients according to rheumatic condition. (n=577)

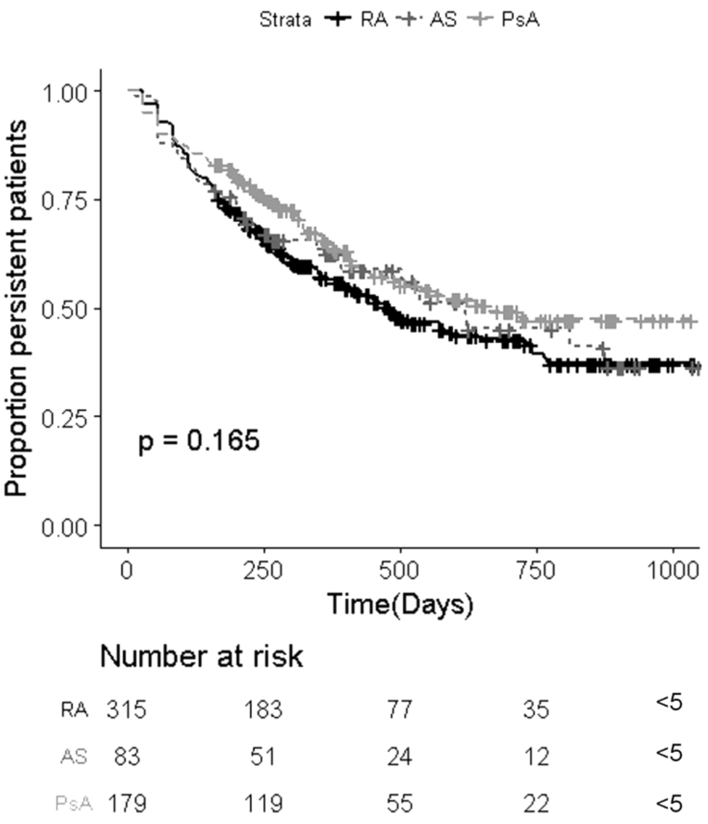

After adjustment for covariates, the difference between RA and PsA patients was still present (HR =0.73, 95%CI:0.54-1.00, p=0.048). Fewer covariates were significant in the incident cohort model. SIMD 3 was associated with a higher risk of discontinuation, whereas SIMD 5 (least deprived quintile) was associated with a lower risk of discontinuation as shown in Supplementary Figure 5. Sex, age and the use of methotrexate or DMARD prior or during the study were not statistically significant covariates.

For peer review only - http://bmjopen.bmj.com/site/about/guidelines.xhtml

Supplementary Figure 5: Adjusted Hazard Ratios for persistence with biologics using Cox regression analysis excluding patients without SIMD values (n=556)

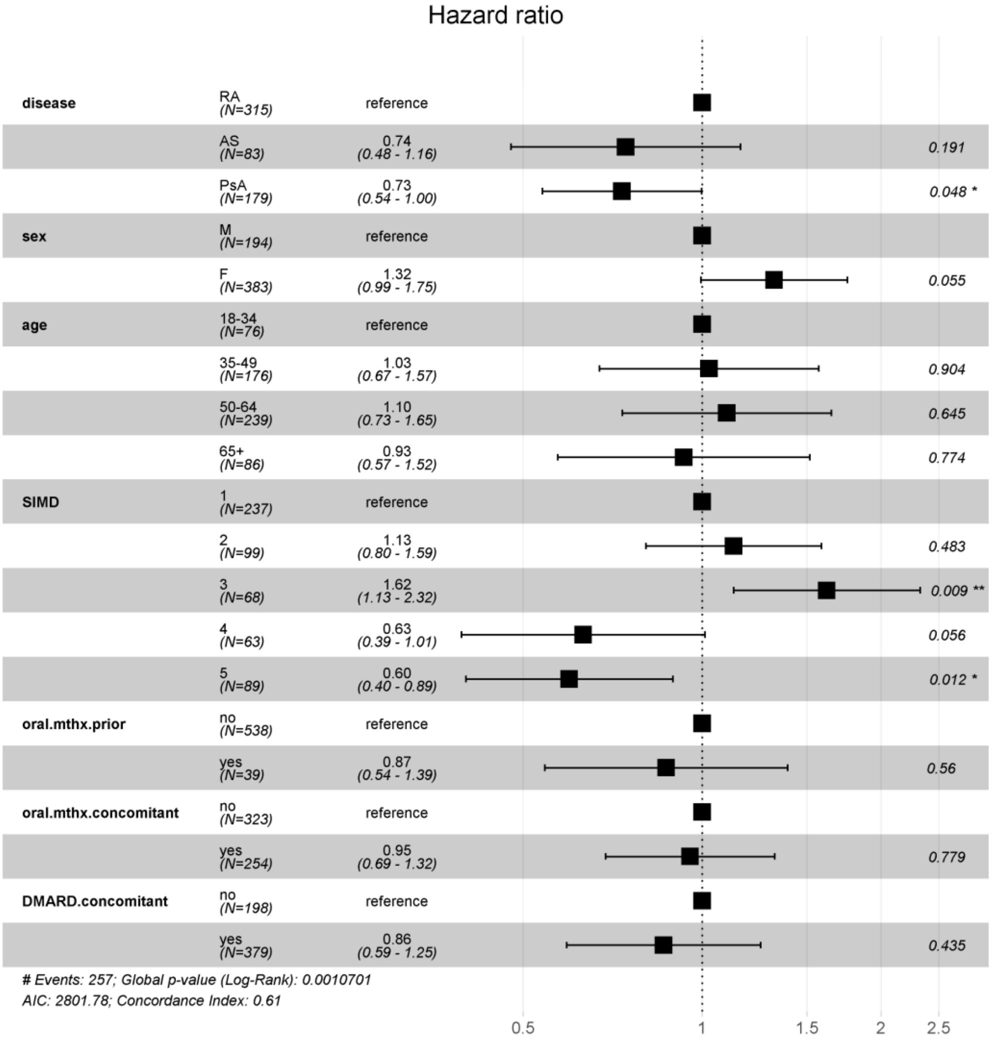

For peer review only - http://bmjopen.bmj.com/site/about/guidelines.xhtml

Adherence

Incident patients with at least two deliveries of the first (index) biologic (n = 598, 88.9%) were used to evaluate adherence. With overall adherence over 80% and both MRA and CR results showing the same patterns as the overall cohort.

Supplementary Figure 6: Adherence to biologics by rheumatic condition according to %MRA (5A) and %CR (5B). MRA- medication refill adherence; CR- compliance rate.

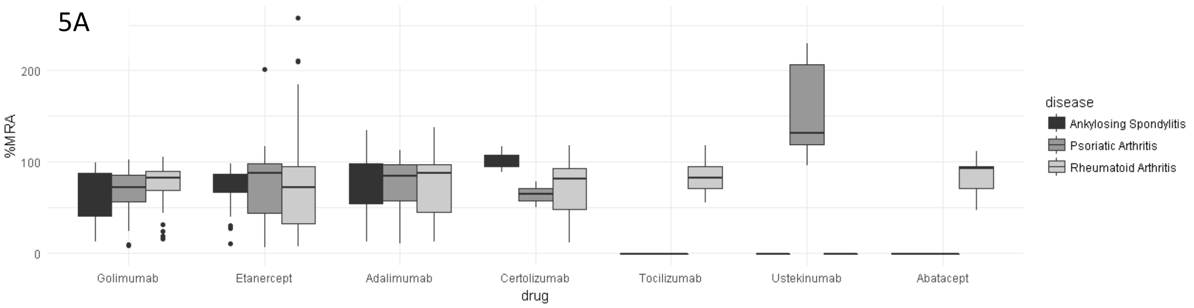

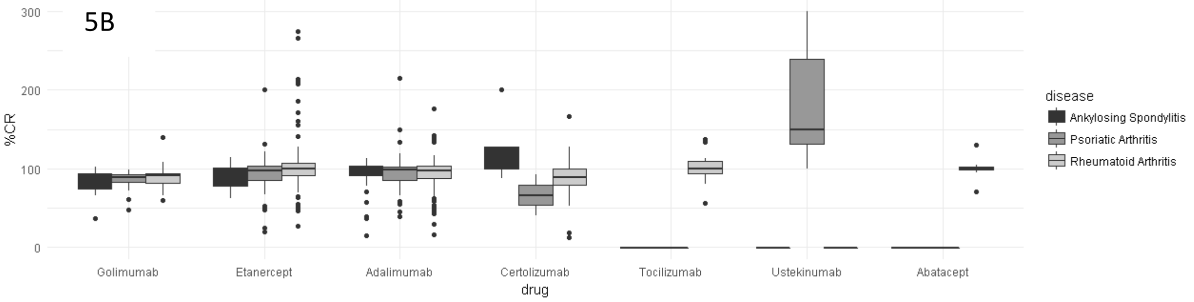

For peer review only

For peer review only - http://bmjopen.bmj.com/site/about/guidelines.xhtml

References

1. Widdifield J, Labrecque J, Lix L, et al. Systematic review and critical appraisal of validation studies to identify rheumatic diseases in health administrative databases. *Arthritis Care Res* 2013; **65**(9): 1490-503.
2. Herrett E, Thomas SL, Schoonen WM,et al. Validation and validity of diagnoses in the General Practice Research Database: a systematic review. *Br J Clin Pharmacol* 2010; **69**(1): 4-14.
3. Dubreuil M, Peloquin C, Zhang Y,et al. Validity of Ankylosing Spondylitis Diagnoses in The Health Improvement Network. *Pharmacoepidemiol Drug Saf* 2016; **25**(4): 399-404.
4. Thomas SL, Edwards CJ, Smeeth L, et al. How accurate are diagnoses for rheumatoid arthritis and juvenile idiopathic arthritis in the general practice research database? *Arthritis Rheum* 2008; **59**(9): 1314-21.

For peer review only

For peer review only - http://bmjopen.bmj.com/site/about/guidelines.xhtml

STROBE 2007 (v4) Statement—Checklist of items that should be included in reports of *cohort studies*

Section/Topic	Item #	Recommendation	Reported on page #
**Title and abstract**	1	(*a*) Indicate the study's design with a commonly used term in the title or the abstract	1
		(*b*) Provide in the abstract an informative and balanced summary of what was done and what was found	2
**Introduction**			
Background/rationale	2	Explain the scientific background and rationale for the investigation being reported	3
Objectives	3	State specific objectives, including any prespecified hypotheses	3
**Methods**			
Study design	4	Present key elements of study design early in the paper	4
Setting	5	Describe the setting, locations, and relevant dates, including periods of recruitment, exposure, follow-up, and data collection	4-5
Participants	6	(*a*) Give the eligibility criteria, and the sources and methods of selection of participants. Describe methods of follow-up	4-5
		(*b*) For matched studies, give matching criteria and number of exposed and unexposed	NA
Variables	7	Clearly define all outcomes, exposures, predictors, potential confounders, and effect modifiers. Give diagnostic criteria, if applicable	5-6
Data sources/ measurement	8*	For each variable of interest, give sources of data and details of methods of assessment (measurement). Describe comparability of assessment methods if there is more than one group	4-6
Bias	9	Describe any efforts to address potential sources of bias	13
Study size	10	Explain how the study size was arrived at	4
Quantitative variables	11	Explain how quantitative variables were handled in the analyses. If applicable, describe which groupings were chosen and why	5
Statistical methods	12	(*a*) Describe all statistical methods, including those used to control for confounding	5-6
		(*b*) Describe any methods used to examine subgroups and interactions	5-6
		(*c*) Explain how missing data were addressed	5
		(*d*) If applicable, explain how loss to follow-up was addressed	NA
		(*e*) Describe any sensitivity analyses	4-5, supplementary file

For peer review only

**Results**			
Participants	13*	(a) Report numbers of individuals at each stage of study—eg numbers potentially eligible, examined for eligibility, confirmed eligible, included in the study, completing follow-up, and analysed	4
		(b) Give reasons for non-participation at each stage	4
		(c) Consider use of a flow diagram	4
Descriptive data	14*	(a) Give characteristics of study participants (eg demographic, clinical, social) and information on exposures and potential confounders	6-9
		(b) Indicate number of participants with missing data for each variable of interest	7-9
		(c) Summarise follow-up time (eg, average and total amount)	8
Outcome data	15*	Report numbers of outcome events or summary measures over time	10
Main results	16	(*a*) Give unadjusted estimates and, if applicable, confounder-adjusted estimates and their precision (eg, 95% confidence interval). Make clear which confounders were adjusted for and why they were included	10-11
		(*b*) Report category boundaries when continuous variables were categorized	7-9
		(*c*) If relevant, consider translating estimates of relative risk into absolute risk for a meaningful time period	NA
Other analyses	17	Report other analyses done—eg analyses of subgroups and interactions, and sensitivity analyses	10-11, supplementary file
**Discussion**			
Key results	18	Summarise key results with reference to study objectives	11
**Limitations**			
Interpretation	20	Give a cautious overall interpretation of results considering objectives, limitations, multiplicity of analyses, results from similar studies, and other relevant evidence	12-14
Generalisability	21	Discuss the generalisability (external validity) of the study results	13
**Other information**			
Funding	22	Give the source of funding and the role of the funders for the present study and, if applicable, for the original study on which the present article is based	17

*Give information separately for cases and controls in case-control studies and, if applicable, for exposed and unexposed groups in cohort and cross-sectional studies.

For peer review only

Note: An Explanation and Elaboration article discusses each checklist item and gives methodological background and published examples of transparent reporting. The STROBE checklist is best used in conjunction with this article (freely available on the Web sites of PLoS Medicine at http://www.plosmedicine.org/, Annals of Internal Medicine at http://www.annals.org/, and Epidemiology at http://www.epidem.com/). Information on the STROBE Initiative is available at www.strobe-statement.org.

For peer review only

BMJ Open

Discontinuation, persistence and adherence to subcutaneous biologics delivered via a homecare route to Scottish adults with rheumatic diseases: a retrospective study.

Journal:	*BMJ Open*
Manuscript ID	bmjopen-2018-027059.R2
Article Type:	Research
Date Submitted by the Author:	05-Jun-2019
Complete List of Authors:	Alvarez-Madrazo, Samantha; University of Strathclyde, Health Data Research Scotland,Strathclyde Institute of Pharmacy and Biomedical Sciences<br>Kavanagh, Kimberley; University of Strathclyde Department of Mathematics and Statistics<br>Siebert, Stefan<br>Semple, Yvonne; University of Strathclyde, Strathclyde Institute of Pharmacy and Biomedical Sciences; Glasgow Royal Infirmary, Medicines Information, Pharmacy Department<br>Godman, Brian; Karolinska University Hospital, 5 Division of Clinical Pharmacology, Karolinska Institutet,<br>Maciel Almeida, Alessandra; Universidade Federal de Minas Gerais<br>Acurcio, Francisco ; Universidade Federal de Minas Gerais<br>Bennie, Marion; University of Strathclyde, Health Data Research Scotland, Strathclyde Institute of Pharmacy and Biomedical Sciences; NHS National Services Scotland, Information Services Division
**Primary Subject Heading**:	Rheumatology
Secondary Subject Heading:	Health informatics, Pharmacology and therapeutics
Keywords:	biologics, psoriatic arthritis, rheumatoid arthritis, ankylosing spondylitis, persistence, adherence

SCHOLARONE™
Manuscripts

TITLE PAGE

Title: Discontinuation, persistence and adherence to subcutaneous biologics delivered via a homecare route to Scottish adults with rheumatic diseases: a retrospective study

Authors:

[1]Samantha Alvarez-Madrazo

[2]Kimberley Kavanagh

[3]Stefan Siebert

[1,4]Yvonne Semple

[1,5] Brian Godman

[6]Alessandra Maciel Almeida

[6]Francisco de Assis Acurcio

[1,7] Marion Bennie

Author affiliations:

[1]Health Data Research Scotland, Strathclyde Institute of Pharmacy and Biomedical Sciences, University of Strathclyde, Glasgow, UK
[2] Department of Mathematics and Statistics, University of Strathclyde, Glasgow,UK
[3] Institute of Infection Immunity and Inflammation, University of Glasgow,UK
[4] Medicines Information, Pharmacy Department, Glasgow Royal Infirmary,UK
[5] Division of Clinical Pharmacology, Karolinska Institutet, Karolinska University Hospital, Stockholm, Sweden
[6] Federal University of Minas Gerais, Brazil
[7] Information Services Division, NHS National Services Scotland, Edinburgh,UK

Corresponding author:

Prof. Marion Bennie

Strathclyde Institute of Pharmacy and Biomedical Sciences

University of Strathclyde

161 Cathedral Street

Glasgow

G4 0RE

Email: marion.bennie@strath.ac.uk

Tel. +44(0) 141 548 2113

Word count: 3988

For peer review only

ABSTRACT

Objectives: to understand patterns of subcutaneous (SC) biologic use over time in adults with inflammatory rheumatic musculoskeletal diseases receiving a homecare delivery service.

Design: retrospective cohort

Setting: patients in secondary care receiving SC biologics in the largest Scottish Health Board

Participants: a new bespoke cohort was created from routine data gathered as part of a health board Homecare Service Database. Patients over 18 years who received a supply of SC biologic from January 2012 - May 2015 with a diagnosis for rheumatoid arthritis (RA), psoriatic arthritis (PsA) or ankylosing spondylitis (AS) were included.

Outcomes measured: a standardised framework was applied by measuring discontinuation rates, persistence using Kaplan-Meier analysis and Cox regression and adherence using medication refill adherence (MRA) and Compliance Rate (CR).

Results: 751 patients were identified (AS – 105, PsA – 227, RA - 419) of whom 89.3% had more than one biologic delivery (median days' follow-up: AS - 494; PsA – 544; RA - 529) and 83.2% did not switch biologic. For all conditions, approximately half were persistent on their index biologic (52% AS, 54% PsA, 48%RA). Of patients who discontinued treatment, the majority re-initiated with the same biologic (19% AS, 18% PsA and 21% RA). Overall adherence during the period of treatment was over 80% when calculated using MRA (median %MRA - AS 84.0%, PsA 85.0%, RA 82.4%) or CR (median %CR - AS 96.6%, PsA 97%, RA 96.6%).

Conclusion: Use of linked routine data is a sustainable pathway to enable on-going evaluation of biologics use. A more consistent approach to studying use (discontinuation, persistence and adherence metrics) should be adopted to enable comparability of studies.

Keywords: biologics, psoriatic arthritis, rheumatoid arthritis, ankylosing spondylitis,persistence, adherence

Article summary

Strengths and limitations of this study

- This is the first real-world cohort containing type, frequency and dose of seven subcutaneous biologics delivered directly to patients with ankylosing spondylitis, psoriatic arthritis and rheumatoid arthritis.
- The cohort is sustainable being generated as part of routine clinical care, can be easily updated and linked to other administrative datasets and provides a novel way to assess adherence, persistence and discontinuation of biologics.
- The limitations include having a mixed cohort of naive and existing biologic users, not capturing reasons for variablity in patients' response, use of intravenous biologics or prior use of any biologic or subcutaneous methotrexate.
- The generalisability of the results in a Scottish setting has to be tested, as this cohort covers the largest Scottish Health Board.

For peer review only - http://bmjopen.bmj.com/site/about/guidelines.xhtml

INTRODUCTION

Chronic inflammatory rheumatic musculoskeletal diseases such as rheumatoid arthritis (RA), psoriatic arthritis (PsA) and ankylosing spondylitis (AS) are characterised by inflammatory joint symptoms requiring long-term management. Over the past two decades, the use of biologic therapies has revolutionised their treatment, with national and global groups recommending their use in patients with moderate to severe disease unresponsive to conventional therapies.[1-5] Whilst clinical trials have demonstrated their short-term efficacy and safety, long-term evidence require observational studies and real-world data. Despite reassuring results to-date,[6-10] important challenges and unanswered questions remain.

Furthermore, despite the apparent shared inflammatory mechanisms and therapies, there are significant differences between these inflammatory conditions in terms of prevalence, gender and sex distribution, genetics, pathophysiology and clinical phenotype, including extra-articular manifestations and co-morbidities.[11-16] Therefore, the safety and long-term therapy data for one condition (usually RA due to the larger numbers and longer pharmacological history) cannot simply be assumed to apply to the other conditions, even for the same drug. It is therefore important to evaluate these factors in real-world settings across the range of conditions for which these drugs are used.

In addition to drug and disease-related factors, in clinical practice poor adherence and persistence to therapy are important detrimental factors, contributing to treatment failure and disease progression.[17] Most of the direct comparisons for adherence and persistence are from observational studies, mainly focusing on the first biologics to reach the market/clinical practice (infliximab, adalimumab and etanercept).[17-19] A few studies have incorporated other biologics,[20-26] with more biologics set to be approved in the upcoming years. Although the standardised measurement of adherence and persistence, dividing the process in three phases - initiation, implementation and discontinuation - has been advocated by Vrijens *et al.* and endorsed by the European Society for Patient Adherence, Compliance and Persistence (ESPACOMP),[27] this methodology has mainly been applied to oral medications and not to biologics, which are administered subcutaneously or intravenously, with complex dosing schedules.

Disease or drug registers are expensive, labour-intensive and not optimal for evaluating adherence and persistence, so other methods are required.[17, 18] In many countries, administrative or prescription datasets linked to reimbursement have been used,[28-31] but these do not always indicate whether the participant actually received the medication. Furthermore, biologics usually require specialist prescription so are not routinely captured in primary care prescription datasets.

This study aimed to use a homecare service database for subcutaneous biologics to understand the patterns of biologic use over time in a population of adults with rheumatic diseases receiving biologics.

For peer review only - http://bmjopen.bmj.com/site/about/guidelines.xhtml

METHODS

Data Sources

For this study a new bespoke, retrospective cohort of patients receiving subcutaneous biologics was created by linking administrative electronic health records (EHRs) at a patient level via an unique national (Scottish) health service identifier, known as the Community Health Index (CHI).[32] This dataset included two resources available in the largest Scottish Health Board, NHS Greater Glasgow and Clyde (GGC), with a population covering 1.2 million of the 5.4 million residents in Scotland.[33] The two novel resources were the Homecare Service Database (HSD) and the local Rheumatology Arthritis Database (RAD), in addition to three existing nationally held datasets: the Prescribing Information System (PIS),[34] the mortality records (National Records of Scotland-NRS) and the Scottish Morbidity Records (SMR01).[35] The variables obtained from each dataset are outlined in Figure 1.

Figure 1. Variables obtained from different Scottish datasets.

Of the GGC patients receiving biologics up until early 2018, 98% received subcutaneous biologics uniquely via the homecare delivery route, with delivery details captured in the HSD and managed through the pharmacy distribution system in the health board. Since 2012 the HSD has systematically captured information about patients (e.g. hospital attended, indication for biologics, drugs and dose, administration and delivery frequency, date and quantity delivered), prescribers and healthcare delivery companies. The remaining 2% of patients receiving intravenous biologics are not captured in the HSD as these medicines are administered directly in hospital day wards.

The RAD contains information of patients who have attended rheumatology clinics in two of the four main hospitals in Glasgow since 1997. Diverse clinical variables are recorded, including the main and secondary diagnoses, biologic screening/follow up, comorbidities, other medications and lifestyle factors.

The PIS includes prescriptions prescribed, dispensed and reimbursed within the community setting,[34] NRS captures cause-specific mortality records and SMR01 has episode-level data on all hospital inpatient and day care episodes from hospitals in Scotland.[35]

Study population

Patients who received at least one delivery of subcutaneous biologics in the HSD dataset with a diagnosis for AS, PsA or RA were eligible for this study. The RAD database was used to validate the diagnosis in a subset of HSD patients (see supplementary file and Supplementary Figure 1 for validation methods and results).

All patients who had a biologic supply date between January 2012 and May 2015 and were at least 18 years old at cohort entry were included. All patients were followed-up until death, migration to a primary care practice outside Scotland or end date of the study (May 2015), whichever occurred first. In the overall cohort, both prevalent and incident users were included (n=751, 105 AS, 227 PsA and 419 RA). To illustrate the stability of findings, a subset analysis was carried out with only incident users (n=673, 95 AS, 208 PsA and 370 RA), defined as patients who did not receive a subcutaneous biologic in the first six months of the study period (from January-June 2012) (online supplementary file).

For peer review only - http://bmjopen.bmj.com/site/about/guidelines.xhtml

One patient was excluded as its first biologic delivery was prior to the date of approval for the drug by the Scottish Medicines Consortium (SMC), which is the national health technology assessment process for all new medicines in Scotland.[36] Also two patients having all missing records in the HSD for dose, frequency or how often the delivery of the biologics would take place were deleted to enable calculations of weeks covered per delivery in the rest the cohort.

The first delivery date for any biologic was established as the index date and the first biologic recorded was considered the index biologic. Age was calculated at index date. Socioeconomic status was assessed using the Scottish Index of Multiple Deprivation quintile (SIMD) 2012 quintiles, a measure that incorporates different aspects of deprivation into a single index. Patients with missing SIMD values remained in the cohort. To assess co-medication, patients with at least one prescription in the 12 months prior to the index date in PIS for antibacterials (British National Formulary- BNF section 5.1), antifungals (BNF section 5.2), antivirals (BNF section 5.3), oral methotrexate (prescribed item methotrexate), Disease-Modifying Anti-Rheumatic Drugs (DMARDs - BNF subsection 10.1.3), Non-Steroidal Anti-inflammatory Drugs (NSAIDs - BNF subsection 10.1.1) and corticosteroids (BNF subsections 1.5.2 or 6.3.2) were recorded.

Data analyses

Patients characteristics were summarized using descriptive statistics. Medians and interquartile ranges are provided for continuous variables and global comparisons were done using the Kruskal-Wallis test. Numbers and percentages are provided for categorical variables and global comparisons were done using either chi-square test or Fisher's exact test (in cases where cells had expected frequencies < 5). To protect confidentiality in the results the GGC Safe Haven carried out a statistical disclosure control. The cell suppression method was applied when the cell values were small and there was a risk of disseminating sensitive information.[37]

In this study, we advocate the use of a standardised framework that includes measurements of discontinuation, persistence and adherence.[27, 38]

Considering the variation in the administration frequencies and number of injections delivered according to biologic, first the estimated days covered per delivery was calculated by multiplying the quantity delivered by the standard administration frequency according to the approved licence for each biologic. Knowing the delivery date and the estimated days covered allowed estimation of the expected end date covered and how it related with the following delivery, and whether there were gaps or not. These metrics were the basis of the utilisation analysis.

Discontinuation was defined as the end of treatment, either because there was a considerable gap of more than 56 days between deliveries (re-initiation) or no more deliveries were received (cessation). Those who did not discontinue were defined as persistent. Discontinuation rates were estimated using the refill-gap method.[39] Patients were censored at first discontinuation, pre-defined as a gap of more than 56 days with no biologics delivery after exhaustion of the days' supplied by the previous delivery, regardless of whether or not patients switched to another biologic. This gap was selected because most biologics were delivered bimonthly, approximately 5 times the half-life for the biologics of

For peer review only - http://bmjopen.bmj.com/site/about/guidelines.xhtml

interest would have elapsed, and this was comparable to other studies.[26, 29] A sensitivity analysis was conducted using a gap of 28 days (minimum period between deliveries) and 84 days (maximum period between deliveries) (see supplementary file online).

Crude persistence was estimated using Kaplan-Meier analysis and Cox regression were used to assess persistence post-index date, in patients with at least 6 months follow-up.The proportional hazards (PH) assumption for a Cox regression model was tested in both the crude and the adjusted model,and stratified where appropriate to ensure a non-significant global p value.[40] Patients who died or migrated were censored.

The hazard ratios (HRs) for persistence with biologics were adjusted for sex, age, SIMD, use of index biologic or not (meaning a switch could have happened whilst being persistent), use of oral methotrexate in the 12 months before to the index date, and concomitant use of oral methotrexate or DMARD during study period.

Adherence is defined as to what extent the biologics delivered correspond to the treatment regimen recommended in the BNF.There is no consensus in the literature on how best to estimate adherence thus two measurements were used for patients with at least two deliveries of the index biologic: medication refill adherence (MRA) and compliance rate (CR).[38, 41] MRA gives an overall adherence percentage describing the exposure during the whole study period regardless of the time when the last prescription was delivered and is calculated as (total days' supply/total days in study) x 100. In contrast, CR describes the exposure between first and last prescription delivered (total days' supply excluding last delivery/days from first delivery up to but not including last delivery) x 100.

Analyses were performed in R software, version 3.5.0.[42]

Patient and public involvement

The prelimary design of this study was presented to the Farr Scotland Public Panel. According to their experience, and preferences the research questions and outcome measures were developed to make the results generalisable to patients with rheumatic conditions.

RESULTS

The study included 751 patients: 105 with AS, 227 with PsA and 419 with RA (Table 1). From them 673 patients (89.6%) were incident users, 95 AS (90.5%), 208 PsA (91.6%) and 370 RA (88.3%).The patients with RA were older and had a higher proportion of females. Regardless of diagnosis, the majority of patients receiving biologics resided in the most deprived areas.

From the overall cohort, a total of 89.3% of patients had more than one delivery of biologic during the study period and 83.2% did not switch biologic. Patients were most commonly started on adalimumab or etanercept as index biologic. The median days' follow-up on biologic therapy, regardless of whether or not the patients switched to others biologics, was: 494 days in AS; 544 days in PsA and 529 days in RA patients.

For peer review only - http://bmjopen.bmj.com/site/about/guidelines.xhtml

Use of concomitant non-biologic medication during the study period was more frequent amongst patients with RA with oral methotrexate (54.9%), any DMARDs (81.6%) and NSAIDs (63.2%) most commonly prescribed. A similar pattern of medication use was observed in PsA patients. In contrast, in AS patients, NSAIDs (71.4%) were the most commonly co-prescribed medications. Prescription of at least one course of antibiotics in patients during the study period was similar for the three conditions (RA – 62.5%, PsA – 59.9%, AS – 49.5%). Similar trends of concomitant medications were found in the incident cohort (see Supplementary Table 1).

For peer review only

Table 1. Baseline characteristics of patients with rheumatic conditions receiving subcutaneous biologic from Jan 2012- May 2015 (n=751)

Rheumatic disease	All		Ankylosing spondylitis		Psoriatic arthritis		Rheumatoid arthritis		p-value
n	**751**		**105**		**227**		**419**		
**Female - n (%)**	**497**	(66.2)	**35**	(33.3)	**130**	(56.5)	**332**	(78.7)	2.2×10^{-16}
**Age***									
Age (median, IQR)*	**53.0(42.7-60.9)**		**47.2 (35.4-55.0)**		**48.0(40.2-57.3)**		**56.0(47.4-64.2)**		4.3×10^{-14}
**Age by category**									5.3×10^{-12}
**18-34 (%)**	**93**	(12.4)	**24**	(22.9)	**36**	(15.9)	**33**	(7.9)	
**35-49 (%)**	**222**	(29.6)	**36**	(34.3)	**91**	(40.1)	**95**	(22.7)	
**50-64 (%)**	**313**	(41.7)	**36**	(34.3)	**82**	(36.1)	**195**	(46.5)	
**65+ (%)**	**123**	(16.4)	**9**	(8.6)	**18**	(7.9)	**96**	(22.9)	
SIMD †									0.326
**1 Most deprived (%)**	**305**	(40.6)	**42**	(42.9)	**93**	(42.1)	**170**	(41.5)	
**2 (%)**	**132**	(17.6)	**21**	(21.4)	**44**	(19.9)	**67**	(16.3)	
**3 (%)**	**81**	(10.8)	**13**	(13.3)	**23**	(10.4)	**45**	(11)	
**4 (%)**	**93**	(12.4)	**15**	(15.3)	**25**	(11.3)	**53**	(12.9)	
**5 Least deprived (%)**	**118**	(15.7)	**7**	(7.1)	**36**	(16.3)	**75**	(18.3)	
Number of deliveries of biologics									
Number of deliveries of biologics (median, IQR)	**6(3-12)**		**7(3-11)**		**6(3-12)**		**6(3-12)**		0.579
Number of deliveries of biologics by category (%)									0.752

1	**80**	(10.7)	**14**	(13.3)	**19**	(8.4)	**47**	(11.2)	
2-10	**456**	(60.7)	**59**	(56.2)	**141**	(62.1)	**256**	(61.1)	
11-20	**156**	(20.8)	**25**	(23.8)	**49**	(21.6)	**82**	(19.6)	
21+	**59**	(7.8)	**7**	(6.7)	**18**	(7.9)	**34**	(8.1)	
Number of different biologics delivered (%)									0.108
1	**625**	(83.2)	**89**	(84.8)	**179**	(78.9)	**357**	(85.2)	
2, 3 or 4 (switchers)	**126**	(16.8)	**16**	(15.2)	**48**	(21.1)	**62**	(14.8)	
First biologic delivered (%)									8.3×10^{-13}
**Adalimumab**	**305**	(40.6)	**55**	(52.4)	**112**	(49.3)	**138**	(32.9)	
**Certolizumab Pegol**	**77**	(10.3)	**6**	(5.7)	**<5**		**69**	(16.5)	
**Etanercept**	**240**	(32)	**24**	(22.9)	**82**	(36.1)	**134**	(32)	
**Golimumab**	**93**	(12.4)	**20**	(19)	**24**	(10.6)	**49**	(11.7)	
**Abatacept**	**8**	(1.1)	**NA**		**NA**		**8**	(1.9)	NA‡
**Tocilizumab**	**21**	(2.8)	**NA**		**NA**		**21**	(5)	
**Ustekinumab**	**7**	(0.9)	**NA**		**7**	(3.1)	**NA**		
**Follow-up (days), median (IQR)**	**529**	**(284.0-852.0)**	**494 (285-838)**		**544 (280-831)**		**529 (283.5-866.5)**		0.894
Concomitant medication use within one year before index date (%)									6.8×10^{-3}
**Antibacterial use**	**48**	(6.4)	**6**	(5.7)	**11**	(4.8)	**31**	(7.4)	
**Oral methotrexate use**	**89**	(11.9)	**<5**		**23**	(10.1)	**63**	(15)	
**Any DMARD**	**115**	(15.3)	**<5**		**28**	(12.3)	**84**	(20)	

For peer review only - http://bmjopen.bmj.com/site/about/guidelines.xhtml

**NSAID**	**114**	(15.2)	**17**	(16.2)	**28**	(12.3)	**69**	(16.5)	
**Gluco or corticosteroids**	**19**	(2.5)	**<5**		**<5**		**16**	(3.8)	
**Antifungal use**	**<10**		**NA**		**<5**		**6**	(1.4)	NA‡
**Antiviral use**	**<5**		**NA**		**NA**		**<5**		
**Subcutaneous methotrexate use**	**NA**		**NA**		**NA**		**NA**		
Concomitant medication use during study period (%)									$1.0x10^{-7}$
**Antibacterial use**	**450**	(59.9)	**52**	(49.5)	**136**	(59.9)	**262**	(62.5)	
**Antifungal use**	**72**	(9.6)	**9**	(8.6)	**26**	(11.5)	**37**	(8.8)	
**Antiviral use**	**53**	(7.1)	**<5**		**17**	(7.5)	**34**	(8.1)	
**Oral methotrexate use**	**332**	(44.2)	**10**	(9.5)	**92**	(40.5)	**230**	(54.9)	
**Any DMARD**	**493**	(65.6)	**12**	(11.4)	**139**	(61.2)	**342**	(81.6)	
**NSAID**	**491**	(65.4)	**75**	(71.4)	**151**	(66.5)	**265**	(63.2)	
**Gluco or corticosteroids**	**148**	(19.7)	**10**	(9.5)	**29**	(12.8)	**109**	(26.0)	
**Subcutaneous methotrexate use**	**14**	(1.9)	**NA**		**<5**		**11**	(2.6)	NA‡
**Comorbidities (%)§**									
Charlson score									$<2.2x10^{-16}$
0	**163**	(21.7)	**38**	(36.2)	**75**	(33)	**50**	(11.9)	
1	**190**	(25.3)	..		..		**167**	(39.9)	
2+	**34**	(4.5)	..		..		**28**	(6.7)	
unknown	**364**	(48.5)	**58**	(55.2)	**132**	(58.1)	**174**	(41.5)	

*Age at first delivery, † from 751 patients 22 did not have Scottish Index of Multiple Deprivation (SIMD) values. Follow-up is from first delivery to study end date. NA‡ Comparison was not calculable because numbers in some cells were not available, were zero and/or too small. § Following the statistical disclosure protocol of the Greater Glasgow and Clyde Safe Haven to avoid attribute disclosure, the cell suppression (primary and secondary) method was used for values in the Comorbidities section as some were too small . DMARD- disease-modifying anti-rheumatic drugs; NSAID- non-steroidal anti-inflammatory drug

For peer review only - http://bmjopen.bmj.com/site/about/guidelines.xhtml

Discontinuation and persistence

For all three conditions, approximately half of the patients were persistent on their index biologic throughout the study period (52% AS, 54% PsA, 48%RA). This was also observed in the incident cohort (55% AS, 55% PsA, 49%RA) as shown in Figure 2 for the overall cohort and Supplementary Figure 2.

Some patients after a period of discontinuation re-initiated the same biologic received at the index date (overall cohort 19% AS, 18% PsA and 21% RA; incident cohort 18%AS, 18% PsA, 19%RA).

A small proportion of patients switched. There were patients changing their index to another biologic either whilst being persistent (overall cohort 4% AS, 9% PsA and 5% RA; incident cohort 4% AS, 9% PsA and 6% RA) or re-initiating after a discontinuation, with a gap longer than 56 days between deliveries (overall cohort 7% AS, 4% PsA and 1% RA; incident cohort 5% AS, 3% PsA and 1% RA).

The remaining patients had a discontinuation and ceased treatment, meaning they did not restart a subcutaneous biologic during the study period (overall and incident cohort 18% AS, 15% PsA and 25% RA). A sensitivity analysis was conducted using a gap of 28 and 84 days showing similar trends (results for overall and incident cohorts are in Supplementary Figure 3).

Figure 2. Persistence and discontinuation (re-initiating or ceasing) of treatment with biologics according to rheumatic condition (n=751)

The crude survival curve of *time to discontinuation* (first gap greater than 56 days between biologic deliveries) of any biologic is shown in Figure 3. In the crude model, the proportion of patients persisting is slightly higher in PsA and AS compared to RA; however, there is no significant difference according to the log-rank test (p=0.0983). The same results were observed in the incident cohort (p=0.165; Supplementary Figure 4). The PH assumption was not violated with all the patients (global p = 0.757) or in the incident cohort (global p = 0.953).

Figure 3:Crude survival curves comparing persistent patients according to rheumatic condition. (n=655)

After adjustment for covariates, there was a slight difference between RA and PsA patients (HR =0.71, 95%CI:0.54-0.95, p=0.02). Lower risk of discontinuation was associated with the two least deprived quintiles (SIMD 4 or 5), whereas female sex and SIMD 3 (deprived quintile) were associated with a higher risk of discontinuation as shown in Figure 4. Age, SIMD 2 (more deprived quintile), prior use of of oral methotrexate, concomitant use of oral methotrexate or DMARD were not statistically signficant covariates and did not influence the model, which excluded 21 patients with missing SIMD values. A subanalysis was performed with the incident cohort in which there was no longer a difference between sex and SIMD4, however, the results of rheumatic conditions and SIMD 3 and SIMD5 quintiles

For peer review only - http://bmjopen.bmj.com/site/about/guidelines.xhtml

remained significant (see Supplementary Figure 5). As those who used the index biologic or switched was used as a stratifying variable, no effect size is estimated for this covariate. The PH assumption was not violated when estimating the adjusted model with all the patients (global p = 0.545) or the incident cohort (global p = 0.192).

Figure 4: Adjusted Hazard Ratios for persistence with biologics using Cox regression analysis excluding patients without SIMD values (n=634)

Adherence

Patients with at least two deliveries of the first (index) biologic (n = 671, 89.3%) were used to evaluate adherence, calculated using two methods.

Overall adherence during the period of treatment was over 80% when calculated using either the MRA or CR. However, this was consistently higher using %CR (median(IQR) for AS 96.6% (86.6-103.5), PsA 97% (85.5-103.2) and RA 96.6% (85.9-102.7) than using %MRA (median(IQR) for AS 84.0% (54.73-94.74), PsA 85.0% (50.76-98.25) and RA 82.4% (47.24-95.77)). The same pattern was also observed when comparing these two methods by the first biologic received (Figure 5A and 5B). Regardless of which of the two measurements were used, the few PsA patients (n=7) taking ustekinumab had higher adherence than was seen with any other biologic. Similar adherence results were obtained in the subset with incident users (Supplementary Figure 6).

Figure 5: Adherence to biologics by rheumatic condition according to %MRA (5A) and %CR (5B). MRA- medication refill adherence; CR- compliance rate.

DISCUSSION

This study is the first real-world cohort using data about SC biologic deliveries, combined with routine data from rheumatology clinics, community pharmacy dispensing, hospital discharges and mortality records to assess the utilisation of biologics in patients with RA, AS and PsA over a period of three years. This study found for all three conditions, approximately 50% of the patients were persistent on their index biologic and that overall adherence during the period of treatment was over 80%. Almost half of patients who discontinued their index biologic for a period greater than 56 days, ultimately re-initiate the same therapy with only a minority switching to another biologic. This highlights the importance of longitudinal data as many of these patients would have been incorrectly counted as cessations in other datasets.

A diverse range of studies have reported on the use of biologics over the last 20 years with the majority focused solely on RA,[19, 29, 43] and few studies comparing utilisation across the three main inflammatory rheumatologic conditions within a single healthcare system. Patient demographic characteristics in our study are similar to those reported in other epidemiological studies in the UK, Denmark, Norway and Germany,[11-16] despite the different methodologies used to create these cohorts.

For peer review only - http://bmjopen.bmj.com/site/about/guidelines.xhtml

Linked prescription and administrative health datasets have been used to study the utilisation of biologics in real-world settings in other studies in Europe, USA, Canada and Brazil. [24, 28, 29, 44, 45] However, to the best of our knowledge, this is the first study to use routine data generated through "direct to home" deliveries of biologics as a source of information to assess how patients use these medicines. Most other routine administrative healthcare studies use prescriptions or claims/reimbursement data. In contrast, this homecare delivery service requires active participation of the patient in both ordering and then signing for receipt of the biologic, thereby confirming the biologic was prescribed, the prescription was processed and the patient received the biologic, providing better insight into how patients are using their medicines.

To understand the use of medicines in our cohort, we adopted the taxonomy proposed by ESPACOMP.[27] In summary, the framework enables us to quantify a patient's medicine taking behaviour in three respects: intensity, continuity and duration - how much of a drug does a patient take, how frequently, and for how long? *Persistence* and *discontinuation* allow us to study continuity and duration of treatment; while *adherence* enables us to study intensity of treatment in the context of the prescribed treatment regimen.

In those studies which examined persistence and discontinuation with biologics across rheumatic conditions, using a consistent in-study method over a comparable time period to our study, persistence was reported as being lower in RA than AS.[45, 46] This is similar to our findings where patients with RA were most likely to discontinue their index subcutaneous biologic, potentially reflecting the treat-to-target approach and greater therapeutic options available for RA at the time of this study. Our study also enabled quantification and categorisation of biologic switching, we believe for the first time, in both patients who were persistent regardless of changes in therapy and those who discontinued index therapy and then commenced a new biologic (Figure1).

In our Cox regression analyses, female sex was associated with lower persistence being consistent with several studies.[43, 45, 46] However, our findings regarding socieconomic status contradict those of the Machado *et al.* study as we have shown higher persistence in patients with lower deprivation levels. There are also discrepancies in the association with the use of methotrexate, as Heiberg *et al.* reported a strong association of its concomitant use with persistence in the three rheumatic diseases and we did not find an association. Randomised controlled trials of biologics in patients with PsA have failed to demonstrate superiority of combination therapies.[47, 48] Thus the role of methotrexate as a predictor for persistence remains unclear.

Persistence after the first, second or subsequent courses of biologics is still controversial. A French study found better persistence with first biologic compared to second or third,[49] with similar tendencies reported in Norway by Heiberg *et al.* In contrast, in a large UK national cohort of RA patients, those who switched to a second biologic had higher persistence[50] and the study using the RADIUS registry found similar persistence between first and second biologic.[51] In our study instead of comparing each course, we compared those persisting with their index biologic (first biologic) with those persisting after switching. Our findings were similar to the UK cohort as persistence was higher in those who had switched.

Various studies have examined adherence in a single rheumatoid condition, often RA[18-24, 26-29] but we could only find a single Italian study measuring adherence across RA, AS and

For peer review only - http://bmjopen.bmj.com/site/about/guidelines.xhtml

PsA,[44] though applying a different method to our study. The challenge in comparing results between studies is often the absence of any detail on how adherence was measured and where specified, multiple measures being used. This has been highlighted in a recent systematic review and meta-analysis by Scheiman-Elazary *et al.* of anti-arthritis medication in RA which reported adherence ranging from 10.5%-98.5% with the meta-analysis generating an adherence rate of 66% (95%CI 58-75%), noting that variability may result from different methods and readers should appraise studies according to the validity of the method, the scales and cut-off points.[18] From our experience of using both MRA and CR for adherence measurement we would recommend the use of CR as it is simple to calculate and includes the period between deliveries, thereby removing any ambiguity arising following the last supply of medicine.

In this study, there were cases where %MRA or %CR was above 100% suggesting some patients could have received more biologics than those expected for that period of time or adjustments with deliveries could be made later on which were not captured in the study period. The higher adherence to ustekinumab compared to the other biologics in this study may relate in part to the small numbers, a more recent introduction requiring the delivery of two 45mg vials to achieve the 90mg dose and a shorter use of this agent at the time of the study, although this observation has also been reported in psoriasis cohorts.[52, 53]

This current study has several strengths. This is the first real-world cohort containing type, frequency and dose of subcutaneous biologics delivered directly to patients for these three rheumatic diseases. The cohort is sustainable as it is generated as part of routine clinical care, avoiding the need for additional data-collection burden required in a traditional registry model, it can be easily updated and linked to other administrative datasets, has better coverage than individually-held clinical data and allows the use of biologics to be studied over time. The sustainability is a key factor, enabling biosimilar and new future innovator biologics to be easily captured as these reach the clinic. This model can also be easily extended to other specialities, including dermatology and gastroenterology, where similar biologic therapies are used. Recording the confirmed delivery of drug directly to patients may reduce some of the uncertainty and limitations associated with using prescription or reimbursement administrative datasets where it cannot be determined if patients actually received the drug. Recall bias was minimized by linking routine data and not based on self-reported persistence or adherence, while selection bias was addressed by including all the patients receiving biologics in the largest Scottish Health Board.

This study also has some limitations. The cohort covers a specific, albeit highly populated, region in Scotland and the generalisability of the results has to be tested. It is a mixed cohort of naive and existing biologic users, which could influence the assessment of medication utilisation. However, the results were similar in the overall and incident cohorts. By using CR as an adherence measurement patients discontinuing prior to study completion are not considered.This was overcome in our study by reporting together adherence, persistence and discontinuation.The reasons for discontinuation or treatment interruption are not currently captured in HSD, so it is of limited utility in terms of understanding the factors that influence the use of biologics. History of biologics or subcutaneous methotrexate being used prior to 2012 was not available. However, during the study period the use subcutaneous methotrexate was captured within the HSD, recording a relatively low use. The biologics home delivery service only captures subcutaneous biologics and not intravenous agents, such as infliximab or rituximab, so will have missed where patients were switched to these

For peer review only - http://bmjopen.bmj.com/site/about/guidelines.xhtml

agents. However, the latter are far smaller in volume and may be captured from rheumatology clinic documentation and hospital administrative data where attendance for infusion guarantees receipt of drug.

CONCLUSION

The long-term evaluation of biologics for the treatment of rheumatic and related diseases is a desirable goal on a clinical level, but it is also a requirement from a pharmacological and economic point of view. Traditional drug or disease registers are expensive, resource intensive and difficult to sustain, so strategies using linked administrative and routine clinical data provide the best opportunity to capture the true picture of biologic use. Additionally a homecare delivery database may reduce some of the uncertainties associated with other administrative databases.

In moving forward, we strongly advocate that future studies should consider application of the ESPACOMP framework[25] to better understand the use of biologics i.e. the combination of discontinuation, persistence and adherence metrics thus leading to a more consistent approach which enables more comparability between studies.

Acknowledgements: Farr Institute @ Scotland, GGC Safe Haven team, Norman Lannigan and Kevin Milne for providing the Homecare Database and allow its use for research purposes. FAA acknowledges the support from FAPEMIG, the Minas Gerais State Research Foundation, Brazil.

REFERENCES

1. Coates LC, Kavanaugh A, Mease PJ, *et al*. Group for Research and Assessment of Psoriasis and Psoriatic Arthritis 2015 Treatment Recommendations for Psoriatic Arthritis. *Arthritis & rheumatology (Hoboken, NJ)* 2016; **68**(5): 1060-71.
2. Gossec L, Smolen JS. European League Against Rheumatism (EULAR) recommendations for the management of psoriatic arthritis with pharmacological therapies: 2015 update. *Ann Rheum Dis* 2016; **75**(3): 499-510.
3. NICE. Rheumatoid arthritis in adults: management. Clinical guideline CG79. England: National Institute for Health and Care Excellence; 2015.
4. Smolen JS, Landewe R, Bijlsma J, *et al*. EULAR recommendations for the management of rheumatoid arthritis with synthetic and biological disease-modifying antirheumatic drugs: 2016 update. *Ann Rheum Dis* 2017; **76**(6): 960-77.
5. van der Heijde D, Ramiro S. 2016 update of the ASAS-EULAR management recommendations for axial spondyloarthritis. *Ann Rheum Dis* 2017; **76**(6): 978-91.
6. Macfarlane GJ, Barnish MS, Jones EA, *et al*. The British Society for Rheumatology Biologics Registers in Ankylosing Spondylitis (BSRBR-AS) study: Protocol for a prospective cohort study of the long-term safety and quality of life outcomes of biologic treatment. *BMC Musculoskelet disorders* 2015; **16**: 347.
7. Nam JL, Takase-Minegishi K, Ramiro S. Efficacy of biological disease-modifying antirheumatic drugs: a systematic literature review informing the 2016 update of the EULAR recommendations for the management of rheumatoid arthritis. *Ann Rheum Dis* 2017; **76**(6): 1113-36.
8. Nikiphorou E, Buch MH, Hyrich KL. Biologics registers in RA: methodological aspects, current role and future applications. *Nat Rev Rheumatol* 2017; **13**(8): 503-10.
9. Saad AA, Ashcroft DM, Watson KD, *et al*. Efficacy and safety of anti-TNF therapies in psoriatic arthritis: an observational study from the British Society for Rheumatology Biologics Register. *Rheumatology (Oxford, England)* 2010; **49**(4): 697-705.

For peer review only - http://bmjopen.bmj.com/site/about/guidelines.xhtml

10. Simard JF, Arkema EV, Sundstrom A, *et al*. Ten years with biologics: to whom do data on effectiveness and safety apply? *Rheumatology (Oxford, England)* 2011; **50**(1): 204-13.
11. Dean LE, Jones GT, MacDonald AG, *et al*. Global prevalence of ankylosing spondylitis. *Rheumatology* 2014; **53**(4): 650-7.
12. Egeberg A, Kristensen LE, Thyssen JP, *et al*. Incidence and prevalence of psoriatic arthritis in Denmark: a nationwide register linkage study. *Ann Rheum Dis* 2017; **8**(210963): 2016-210963.
13. Hoff M, Gulati AM, Romundstad PR, *et al*. Prevalence and incidence rates of psoriatic arthritis in central Norway: data from the Nord-Trondelag health study (HUNT). *Ann Rheum Dis* 2015; **74**(1): 60-4.
14. Humphreys JH, Verstappen SM, Hyrich KL, *et al*. The incidence of rheumatoid arthritis in the UK: comparisons using the 2010 ACR/EULAR classification criteria and the 1987 ACR classification criteria. Results from the Norfolk Arthritis Register. *Ann Rheum Dis* 2013; **72**(8): 1315-20.
15. Huscher D, Thiele K, Rudwaleit M, *et al*. Trends in treatment and outcomes of ankylosing spondylitis in outpatient rheumatological care in Germany between 2000 and 2012. *RMD Open* 2015; **1**(1): 2014-000033.
16. Ogdie A, Weiss P. The Epidemiology of Psoriatic Arthritis. *RheumDis Clin North Am* 2015; **41**(4): 545-68.
17. Blum MA, Koo D, Doshi JA. Measurement and rates of persistence with and adherence to biologics for rheumatoid arthritis: a systematic review. *Clin Ther* 2011; **33**(7): 901-13.
18. Scheiman-Elazary A, Duan L, Shourt C, *et al*. The Rate of Adherence to Antiarthritis Medications and Associated Factors among Patients with Rheumatoid Arthritis: A Systematic Literature Review and Metaanalysis. *J Rheumatol* 2016; **43**(3): 512-23.
19. Vangeli E, Bakhshi S, Baker A, *et al*. A Systematic Review of Factors Associated with Non-Adherence to Treatment for Immune-Mediated Inflammatory Diseases. *Adv Ther* 2015; **32**(11): 983-1028.
20. Bhoi P, Bessette L, Bell MJ, *et al*. Adherence and dosing interval of subcutaneous antitumour necrosis factor biologics among patients with inflammatory arthritis: analysis from a Canadian administrative database. *BMJ open* 2017; **7**(9): e015872.
21. Ebina K, Hashimoto M, Yamamoto W, *et al*. Drug retention and discontinuation reasons between seven biologics in patients with rheumatoid arthritis -The ANSWER cohort study. *PloS one* 2018; **13**(3): e0194130.
22. Glintborg B, Ostergaard M, Krogh NS, *et al*. Clinical response, drug survival and predictors thereof in 432 ankylosing spondylitis patients after switching tumour necrosis factor alpha inhibitor therapy: results from the Danish nationwide DANBIO registry. *Ann Rheum Dis* 2013; **72**(7): 1149-55.
23. Kruger K, Burmester GR, Wassenberg S,*et al*. Effectiveness and safety of golimumab in patients with rheumatoid arthritis, psoriatic arthritis and ankylosing spondylitis under real-life clinical conditions: non-interventional GO-NICE study in Germany. *BMJ open* 2018; **8**(6): e021082.
24. Lyu R, Govoni M, Ding Q, *et al*. Treatment persistence among patients with rheumatoid disease (RA, AS, PsA) treated with subcutaneous biologics in Germany. *Rheumatol Int* 2015; **28**: 28.
25. Oei HB, Hooker RS, Cipher DJ,*et al*. High rates of stopping or switching biological medications in veterans with rheumatoid arthritis. *Clin Exp Rheumatol* 2009; **27**(6): 926-34.
26. Wallman JK, Kapetanovic MC, Petersson IF *et al*. Comparison of non-radiographic axial spondyloarthritis and ankylosing spondylitis patients--baseline characteristics, treatment adherence, and development of clinical variables during three years of anti-TNF therapy in clinical practice. *Arthritis Res Ther* 2015; **17**(378): 015-0897.
27. Vrijens B, De Geest S, Hughes DA, et al. A new taxonomy for describing and defining adherence to medications. *Br J Clin Pharmacol* 2012; **73**(5): 691-705.
28. Acurcio FA, Machado MA, Moura CS, *et al*. Medication Persistence of Disease-Modifying Antirheumatic Drugs and Anti-Tumor Necrosis Factor Agents in a Cohort of Patients With Rheumatoid Arthritis in Brazil. *Arthritis Care Res* 2016; **68**(10): 1489-96.

For peer review only - http://bmjopen.bmj.com/site/about/guidelines.xhtml

29. Fisher A, Bassett K, Wright JM,*et al*. Comparative persistence of the TNF antagonists in rheumatoid arthritis--a population-based cohort study. *PloS one* 2014; **9**(8): e105193.
30. Ogale S, Hitraya E, Henk HJ. Patterns of biologic agent utilization among patients with rheumatoid arthritis: a retrospective cohort study. *BMC Musculoskelet Disord* 2011; **12**: 204.
31. Wadstrom H, Eriksson JK, Neovius M, *et al*. How good is the coverage and how accurate are exposure data in the Swedish Biologics Register (ARTIS)? *Scand J Rheumatol* 2015; **44**(1): 22-8.
32. ISD Scotland. ISD Scotland data dictionary. Edinburgh; 2016. http://www.ndc.scot.nhs.uk/Dictionary-A-Z/Definitions/index.asp?Search=C&ID=128&Title=CHI%20Number (accessed 10 December 2017)
33. NRS. Mid-year population estimates. Edinburgh; 2016. http://www. nrscotland.gov.uk/statistics-and-data/statistics/statistics-by-theme/ population/population-estimates/mid-year-population-estimates (accessed 10 December 2017)
34. Alvarez-Madrazo S, McTaggart S, Nangle C,*et al*. Data Resource Profile: The Scottish National Prescribing Information System (PIS). *Int J Epidemiol* 2016; **45**(3): 714-5f.
35. ISD Scotland. National Data Catalogue: National Datasets. Edinburgh; 2017. http://www.ndc.scot.nhs.uk/National-Datasets/ (accessed 10 December 2017)
36. Scottish Medicines Consortium . https://www.scottishmedicines.org.uk/(accessed 22 June 2017)
37. NHS National Services Scotland. Statistical Disclosure Control Protocol. Version 3.0. 2015. https://www.isdscotland.org/About-ISD/Confidentiality/disclosure_protocol_v3.pdf (accessed 30 January 2019)
38. Mueller T, Alvarez-Madrazo S, Robertson C, *et al*.. Use of direct oral anticoagulants in patients with atrial fibrillation in Scotland: Applying a coherent framework to drug utilisation studies. *Pharmacoepidemiol Drug Saf* 2017; **26**(11): 1378-86.
39. Gregoire JM, J. Assessment of adhrence to drug treatment in database records. In: Elseviers M, Wettermak B, Almarsdotti AB.; *et al*., ed. Drug Utilization Research: Methods and Applications. Chichester: Wiley Blackwell; 2016: 369-80.
40. Grambsch PM and Therneau TM. Proportional hazards tests and diagnostics based on weighted residuals. *Biometrika* 1994; **81**: 515-26.
41. Hess LM, Raebel MA, Conner DA, *et al*. Measurement of adherence in pharmacy administrative databases: a proposal for standard definitions and preferred measures. *Ann Pharmacother* 2006; **40**(7-8): 1280-88.
42. R Core Team (2018): A language and environment for statistical computing. R Foundation for Statistical Computing, Vienna, Austria. 2018. http://www.R-project.org/.

43. Souto A, Maneiro JR, Gomez-Reino JJ. Rate of discontinuation and drug survival of biologic therapies in rheumatoid arthritis: a systematic review and meta-analysis of drug registries and health care databases. *Rheumatology (Oxford, England)* 2016; **55**(3): 523-34.

44. Degli Esposti L, Sangiorgi D, Perrone V, et al. Adherence and resource use among patients treated with biologic drugs: findings from BEETLE study. *Clinicoecon Outcomes Res: CEOR* 2014; **6**: 401-7.
45. Machado MA, Moura CS, Ferre F, *et al*. Treatment persistence in patients with rheumatoid arthritis and ankylosing spondylitis. *Rev Saude Publica* 2016; **50**(50): 2016050006265.
46. Heiberg MS, Koldingsnes W, Mikkelsen K, *et al*. The comparative one-year performance of anti-tumor necrosis factor alpha drugs in patients with rheumatoid arthritis, psoriatic arthritis, and ankylosing spondylitis: results from a longitudinal, observational, multicenter study. *Arthritis Rheum* 2008; **59**(2): 234-40.
47. Mease PJ, Gladman DD, Ritchlin CT, *et al*. Adalimumab for the treatment of patients with moderately to severely active psoriatic arthritis: results of a double-blind, randomized, placebo-controlled trial. *Arthritis Rheum* 2005; **52**(10): 3279-89.

For peer review only - http://bmjopen.bmj.com/site/about/guidelines.xhtml

48. Mease PJ, Kivitz AJ, Burch FX, et al. Etanercept treatment of psoriatic arthritis: safety, efficacy, and effect on disease progression. *Arthritis Rheum* 2004; **50**(7): 2264-72.
49. Duclos M, Gossec L, Ruyssen-Witrand A, *et al*. Retention rates of tumor necrosis factor blockers in daily practice in 770 rheumatic patients. *J Rheumatol* 2006; **33**(12): 2433-8.
50. Hyrich KL, Lunt M, Watson KD, *et al*. Outcomes after switching from one anti-tumor necrosis factor alpha agent to a second anti-tumor necrosis factor alpha agent in patients with rheumatoid arthritis: results from a large UK national cohort study. *Arthritis Rheum* 2007; **56**(1): 13-20.
51. Markenson JA, Gibofsky A, Palmer WR, *et al*. Persistence with anti-tumor necrosis factor therapies in patients with rheumatoid arthritis: observations from the RADIUS registry. *J Rheumatol* 2011; **38**(7): 1273-81.
52. Menter A, Papp KA, Gooderham M, *et al*. Drug survival of biologic therapy in a large, disease-based registry of patients with psoriasis: results from the Psoriasis Longitudinal Assessment and Registry (PSOLAR). *J Eur Acad Dermatol Venereol: JEADV* 2016; **30**(7): 1148-58.
53. Zweegers J, van den Reek JM, van de Kerkhof PC, *et al*. Body mass index predicts discontinuation due to ineffectiveness and female sex predicts discontinuation due to side-effects in patients with psoriasis treated with adalimumab, etanercept or ustekinumab in daily practice: a prospective, comparative, long-term drug-survival study from the BioCAPTURE registry. *Br J Dermatol* 2016; **175**(2): 340-7.

FOOTNOTES

Contributors: All authors contributed to study design. SAM extracted, prepared the data and performed the analyses. SAM drafted the manuscript, with revisions by KK, SS, YS,BG,AMA,FAA and MB.

Funding: We acknowledge the support from The Farr Institute @ Scotland. The Farr Institute @ Scotland is supported by a 10-funder consortium: Arthritis Research UK, the British Heart Foundation, Cancer Research UK, the Economic and Social Research Council, the Engineering and Physical Sciences Research Council, the Medical Research Council, the National Institute of Health Research, the National Institute for Social Care and Health Research (Welsh Assembly Government), the Chief Scientist Office (Scottish Government Health Directorates), the Wellcome Trust, (MRC Grant No: MR/K007017/1).

Competing interest: SS has received honoraria from Janssen, Novartis, AbbVie, Pfizer, UCB, Celgene and Boehringer-Ingelheim; and research funding from UCB, Celgene, Boehringer-Ingelheim, BMS and Pfizer.

Patient consent: Not required.

Ethical approval: Ethics permission was not required as only non-identifiable routine data was used for this study. The study was approved by the NHS GGC Safe Haven Local Privacy Advisory Committee (Project number: GSH/15/MT/001). No patient identifiers were available to the study team and all data were accessed via the GGC Safe Haven.

Provenance and peer review: not commissioned; externally peer reviewed.

Data sharing statement: All data supporting this research are openly available to researchers based in recognized UK institutions from http://www.nhsggc.org.uk/about-us/professional-support-sites/nhsggc-safe-haven/your-research/available-datasets. For further information contact the Greater Glasgow and Clyde Safe Haven by emailing safehaven@ggc.scot.nhs.uk

For peer review only - http://bmjopen.bmj.com/site/about/guidelines.xhtml

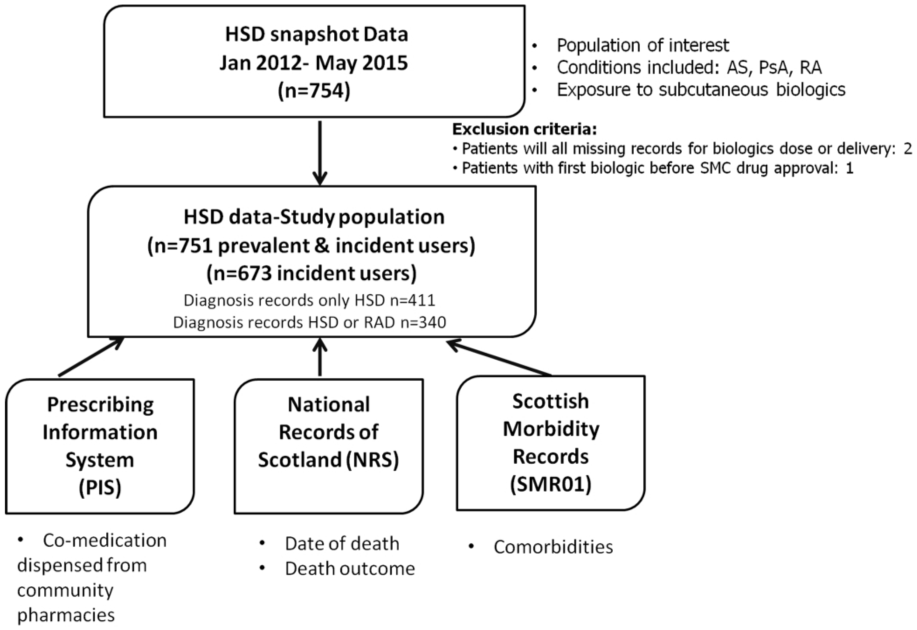

Figure 1. Variables obtained from different Scottish datasets.

306x192mm (300 x 300 DPI)

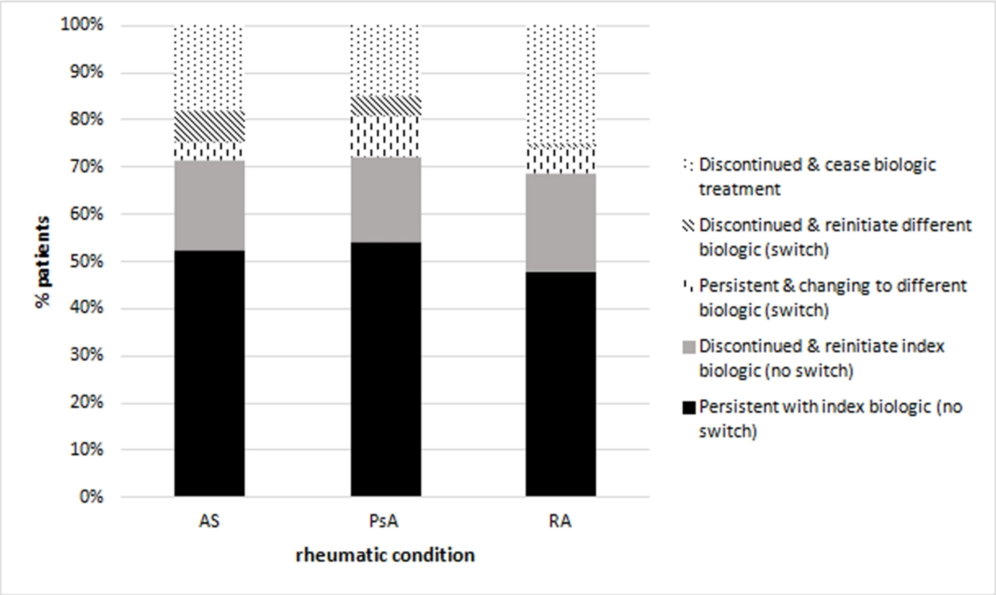

215x129mm (300 x 300 DPI)

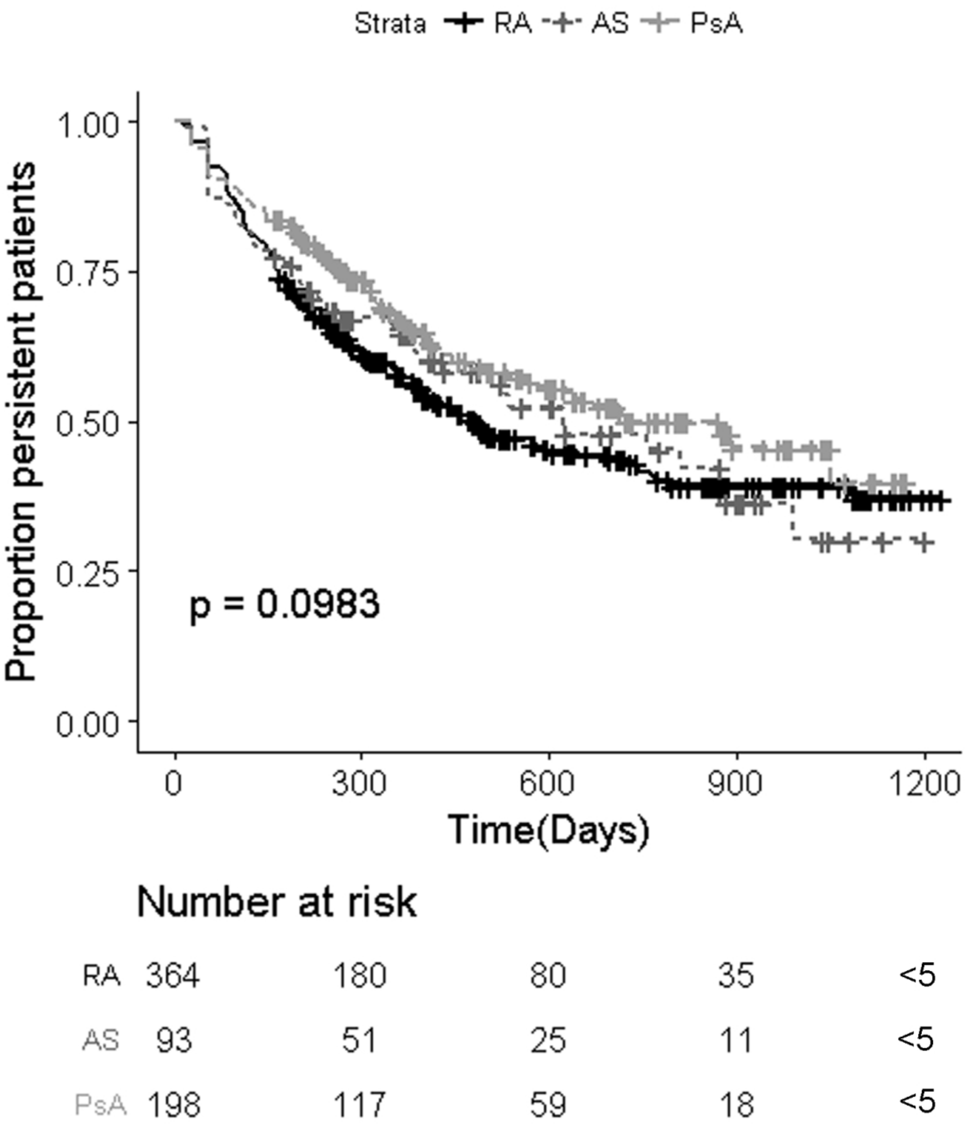

Figure 3:Crude survival curves comparing persistent patients according to rheumatic condition. (n=655)

125x152mm (300 x 300 DPI)

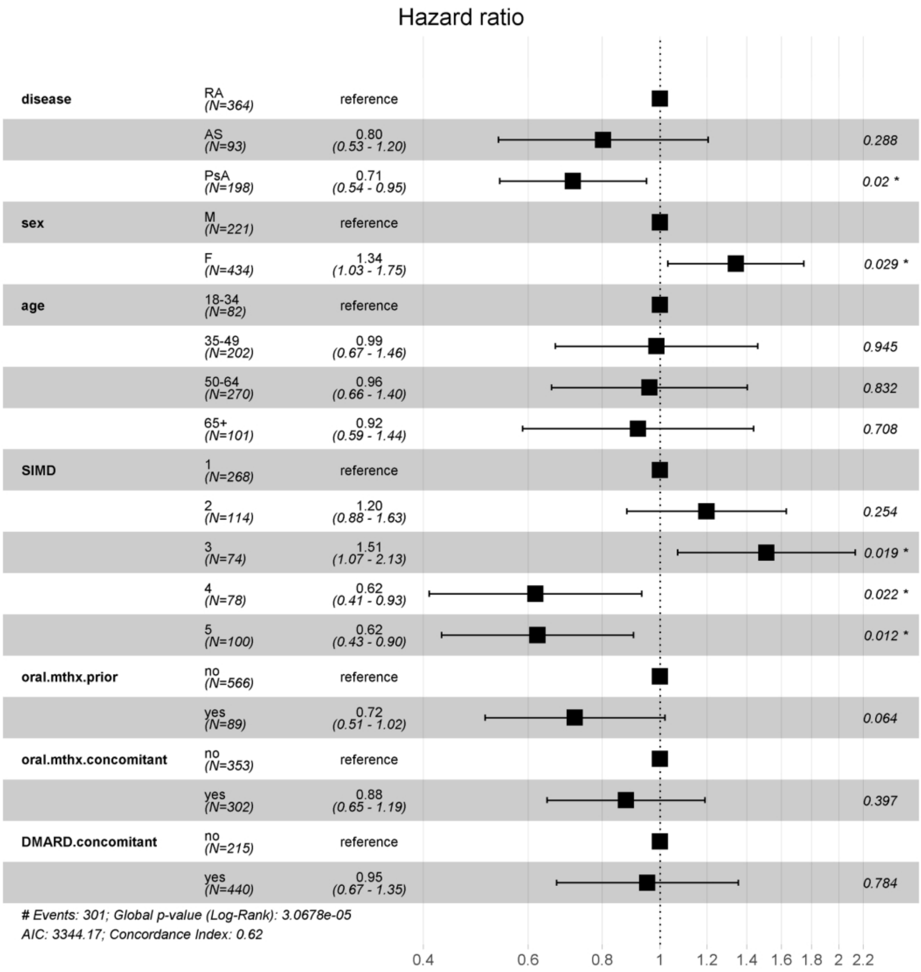

Caption : Figure 4: Adjusted Hazard Ratios for persistence with biologics using Cox regression analysis excluding patients without SIMD values (n=634)

199x199mm (300 x 300 DPI)

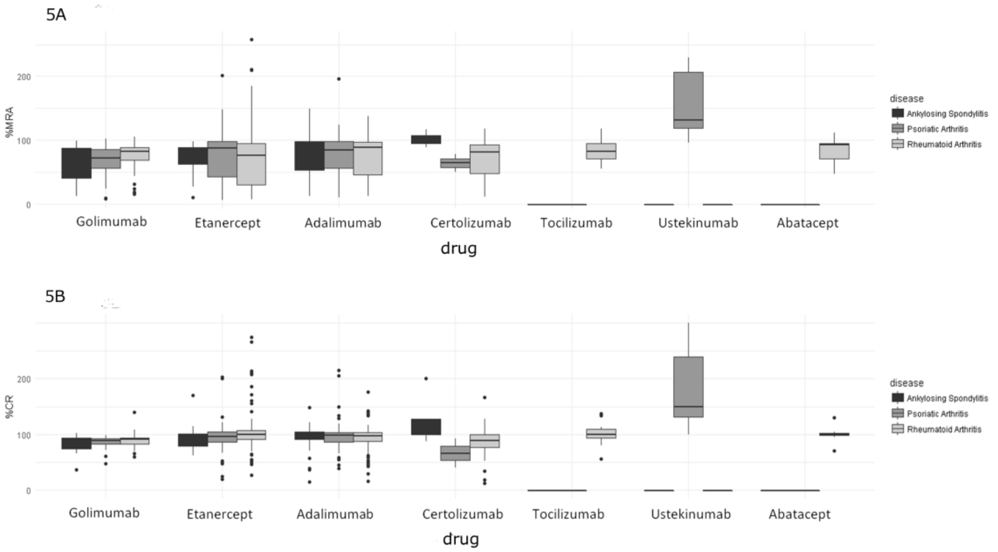

Figure 5: Adherence to biologics by rheumatic condition according to %MRA (5A) and %CR (5B). MRA- medication refill adherence; CR- compliance rate.

Supplementary file :

Validation method and results

Of the 751 patients in HSD, 340 were also in the RAD. A minority of these (n=48) had diagnosis recorded in either of the datasets with the majority of these (n=292) with diagnosis recorded in both datasets (supplementary figure 1). RAD diagnosis was considered as the "gold standard" because it was collected in rheumatology clinics by clinical staff and was used to determine the validity of HSD diagnosis in the 292 patients. There were 3540 patients diagnosed with the three rheumatic conditions (RA, PsA or AS) who attended rheumatology clinics in Glasgow and were only recorded in RAD. These patients were not used for the validation study.

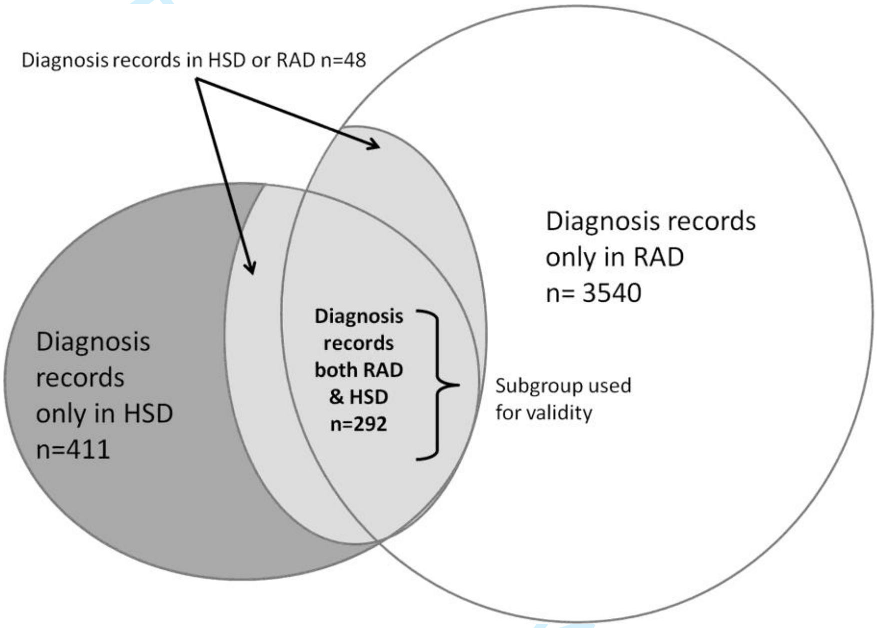

Supplementary Figure 1. Diagnoses of rheumatic conditions according to HSD and RAD records.

Validity of diagnoses of rheumatic conditions (PPV and sensitivity)

The positive predictive value (PPV) and sensitivity with 95% confidence intervals were calculated for the diagnosis of RA, PsA or AS in HSD. PPV was calculated as the proportion of subjects in HSD with an indication for any of these three conditions in first recorded delivery and confirmed diagnosis in RAD. Sensitivity was calculated as the proportion of RAD cases who had a RA, PsA or AS diagnosis from all the RA, PsA or AS patients in the 292 subjects. A high PPV identifies a true population, or this case subgroup, of patients with chronic inflammatory diseases; while a high sensitivity allows to asses as many cases as possible.

Validity of arthritic conditions

From the subset of patients with arthritic conditions in HSD, the diagnosis was confirmed in 292 patients, with a 91.8%(95%CI: 86.4-95.1) PPV and a sensitivity of 97.3% (95%CI:93.3-99.0) for RA, 97.4% (95%CI:86.5-99.9) PPV and 94.9% (95%CI: 83.1-98.6)sensitivity for AS and 95.8% (95%CI:89.8-98.4) PPV and 88.5%(95%CI:80.9-93.3) sensitivity for PsA.

For peer review only - http://bmjopen.bmj.com/site/about/guidelines.xhtml

Many studies focus in the optimal clinical use and safety of medication in rheumatic diseases using administrative or claims data and one of the first requirements, following the best-practice consensus from the CANRAD Network, is the validation of case definitions.[1] There are mainly two types of validation methods (a) internal using algorithms or sensitivity analyses within the data or (b) external using data from other sources such as clinical notes or data captured by general practitioners.[2] In this study, an external validation was performed by comparing the diagnoses of the patients present in both HSD and RAD databases. Both the PPV and sensitivity were above 80% for the three diseases. In contrast to several studies, instead of using ICD-10 codes, our study used free text entered by clinicians in RAD which was compared to the diagnoses entered from the prescriptions in HSD. All the patients also had at least one prescription for a subcutaneous biologic in HSD. Our sample size (n=292) is in the range of similar studies aimed to validate diagnoses (n=85-450), and our PPV values and sensitivities are comparable to others using data from rheumatology clinics (PPV 95-97%; sensitivities reported 30-100%).[3, 4]

Analyses with incident users of subcutaneous biologics

Incident users were defined as patients who did not receive a subcutaneous biologic in the first six months of the study period (from January-June 2012). This incident cohort included 673 patients (89.6% from the original cohort), from them 95 had AS, 208 PsA and 370 RA (Supplementary Table 1). The proportion of patients according to age, gender and the distribution of socioeconomic levels (SIMD) were similar.

A total of 88.9% of incident patients had more than one delivery of biologic during the study period (compared to 89.3% from the original cohort) and the same percentage of 83.2% did not switch biologic.

The index biologics mostly used were also adalimumab and etanercept. Although the proportion of users according to their index biologics did not change, there were less users of adalimumab, certolizumab pegol and etanercept in the incident cohort as the prevalent users of these three medications were excluded. This is in agreement with the dates of approval for their use as these biologics were the first ones approved and patients were more likely to be users before 2012.

The pattern of concomitant medications prevailed in the incident cohort as RA and PsA patients were frequent users of oral methotrexate, DMARDs and NSAIDs, while AS patients mainly used NSAIDs.

For peer review only

Supplementary Table 1. Baseline characteristics of incident patients with rheumatic conditions receiving subcutaneous biologic from July 2012-May 2015 (n=673)

Rheumatic disease	All		Ankylosing spondylitis		Psoriatic arthritis		Rheumatoid arthritis		p-value
n	**673**		**95**		**208**		**370**		
**Female - n (%)**	**446**	(66.3)	**34**	(36.8)	**117**	(56.3)	**295**	(79.7)	$<2.2 \times 10^{-16}$
**Age***									
Age (median, IQR)*	**52.9(42.5-60.4)**		**47.4 (35.6-55.4)**		**48.0(40.1-57.3)**		**55.9(47.1-64.2)**		1.78×10^{-11}
**Age by category**									4.6×10^{-10}
**18-34 (%)**	**87**	(12.9)	**21**	(22.1)	**34**	(16.3)	**32**	(8.6)	
**35-49 (%)**	**196**	(29.1)	**31**	(32.6)	**83**	(39.9)	**82**	(22.2)	
**50-64 (%)**	**282**	(41.9)	**35**	(36.8)	**75**	(36.1)	**172**	(46.5)	
**65+ (%)**	**108**	(16.0)	**8**	(8.4)	**16**	(7.7)	**84**	(22.7)	
SIMD †									0.314
**1 Most deprived (%)**	**274**	(40.7)	**38**	(43.2)	**85**	(42.1)	**151**	(41.8)	
**2 (%)**	**117**	(17.4)	**19**	(21.6)	**40**	(19.8)	**58**	(16.1)	
**3 (%)**	**75**	(11.1)	**13**	(14.8)	**22**	(10.9)	**40**	(11.1)	
**4 (%)**	**78**	(11.6)	**12**	(13.6)	**22**	(10.9)	**44**	(12.2)	
**5 Least deprived (%)**	**107**	(15.9)	**6**	(6.8)	**33**	(16.3)	**68**	(18.8)	
Number of deliveries of biologics									
Number of deliveries of biologics (median, IQR)	**6**	(3-10)	**6**	(3-11)	**6**	(3-11)	**5**	(3-10)	0.400

For peer review only - http://bmjopen.bmj.com/site/about/guidelines.xhtml

Number of deliveries of biologics by category (%)									0.803
1	**75**	(11.1)	**13**	(13.7)	**18**	(8.7)	**44**	(11.9)	
2-10	**434**	(64.5)	**57**	(60.0)	**136**	(65.4)	**241**	(65.1)	
11-20	**129**	(19.2)	**19**	(20.0)	**43**	(20.7)	**67**	(18.1)	
21+	**35**	(5.2)	**6**	(6.3)	**11**	(5.3)	**18**	(4.9)	
Number of different biologics delivered (%)									
1	**560**	(83.2)	**81**	(85.3)	**165**	(79.3)	**314**	(84.9)	0.196
2, 3 or 4 (switchers)	**113**	(16.8)	**14**	(14.7)	**43**	(20.7)	**56**	(15.1)	
First biologic delivered(%)									7.33×10^{-11}
**Adalimumab**	**263**	(39.1)	**47**	(49.5)	**98**	(47.1)	**118**	(31.9)	
**Certolizumab Pegol**	**68**	(10.1)	**6**	(6.3)	**<5**		**60**	(16.2)	
**Etanercept**	**215**	(31.9)	**22**	(23.2)	**77**	(37.0)	**116**	(31.4)	
**Golimumab**	**91**	(13.5)	**20**	(21.1)	**24**	(11.5)	**47**	(12.7)	
**Abatacept**	**8**	(1.2)	**NA**		**NA**		**8**	(2.2)	NA‡
**Tocilizumab**	**21**	(3.1)	**NA**		**NA**		**21**	(5.7)	
**Ustekinumab**	**7**	(1.0)	**NA**		**7**	(3.4)	**NA**		
**Follow-up (days), *(median, IQR)***	**480**	**(264.0-734.0)**	**442 (278-749.5)**		**504.5(267.5-763.5)**		**482.0 (263.3-731.5)**		0.950

Concomitant medication use within one year before index date (%)									
**Antibacterial use**	**20**	(3.0)	**<5**		**<5**		**14**	(3.8)	0.149
**Oral methotrexate use**	**39**	(5.8)	**<5**		**9**	(4.3)	**29**	(7.8)	
**Any DMARD**	**54**	(8.0)	**<5**		**13**	(6.3)	**40**	(10.8)	
**NSAID**	**54**	(8.0)	**8**	(8.4)	**15**	(7.2)	**31**	(8.4)	
**Antifungal use**	**<5**		**NA**		**NA**		**<5**		NA[‡]
**Antiviral use**	**NA**		**NA**		**NA**		**<5**		
**Subcutaneous methotrexate use**	**NA**		**NA**		**NA**		**NA**		
**Gluco or corticosteroids**	**10**	(2.5)	**NA**		**<5**		**9**	(2.4)	
Concomitant medication use during study period (%)									1.0×10^{-7}
**Antibacterial use**	**394**	(58.5)	**46**	(48.4)	**124**	(59.6)	**224**	(60.5)	
**Antifungal use**	**62**	(9.2)	**7**	(7.4)	**22**	(10.6)	**33**	(8.9)	
**Antiviral use**	**44**	(6.5)	**<5**		**16**	(7.7)	**26**	(7.0)	
**Oral methotrexate use**	**284**	(42.2)	**7**	(7.4)	**78**	(37.5)	**199**	(53.8)	
**Any DMARD**	**432**	(64.2)	**9**	(9.5)	**122**	(58.7)	**301**	(81.4)	
**NSAID**	**430**	(63.9)	**65**	(68.4)	**137**	(65.9)	**228**	(61.6)	
**Gluco or corticosteroids**	**132**	(19.6)	**10**	(10.5)	**26**	(12.5)	**96**	(25.9)	
**Subcutaneous methotrexate use**	**10**	(1.5)	**NA**		**<5**		**7**	(1.9)	NA[‡]

**Comorbidities (%)**[§]									$<2.2x10^{-16}$
Charlson score									
0	**145**	(21.5)	**32**	(30.5)	**66**	(31.7)	**47**	(12.7)	
1	**164**	(24.4)	..		..		**143**	(38.6)	
2+	**33**	(4.9)	..		..		**27**	(7.3)	
unknown	**331**	(49.2)	**55**	(52.4)	**123**	(59.1)	**153**	(41.4)	

*Age at first delivery, † from 673 patients 22 did not have Scottish Index of Multiple Deprivation (SIMD) values. Follow-up is from first delivery to study end date. NA[‡] Comparison was not calculable because numbers in some cells were not available, were zero and/or too small. § Following the statistical disclosure protocol of the Greater Glasgow and Clyde Safe Haven to avoid attribute disclosure, the cell suppression (primary and secondary) method was used for for values in the Comorbidities section as some were too small. DMARD- disease-modifying anti-rheumatic drugs; NSAID- non-steroidal anti-inflammatory drug

For peer review only - http://bmjopen.bmj.com/site/about/guidelines.xhtml

Discontinuation and persistence

Also in the incident cohort approximately half of the patients were persistent on their index biologic throughout the study period (55% AS, 55% PsA, 49%RA) as shown in Supplementary Figure 2. From those who discontinue treatment the majority reintiate it with their index biologic (18%AS, 18% PsA, 19%RA) and a few switch to other biologics (5%AS, 3% PsA, 1%RA).

Supplementary Figure 2. Persistence and discontinuation (re-initiating or ceasing) of treatment with biologics according to rheumatic condition in the incident cohort (n=673)

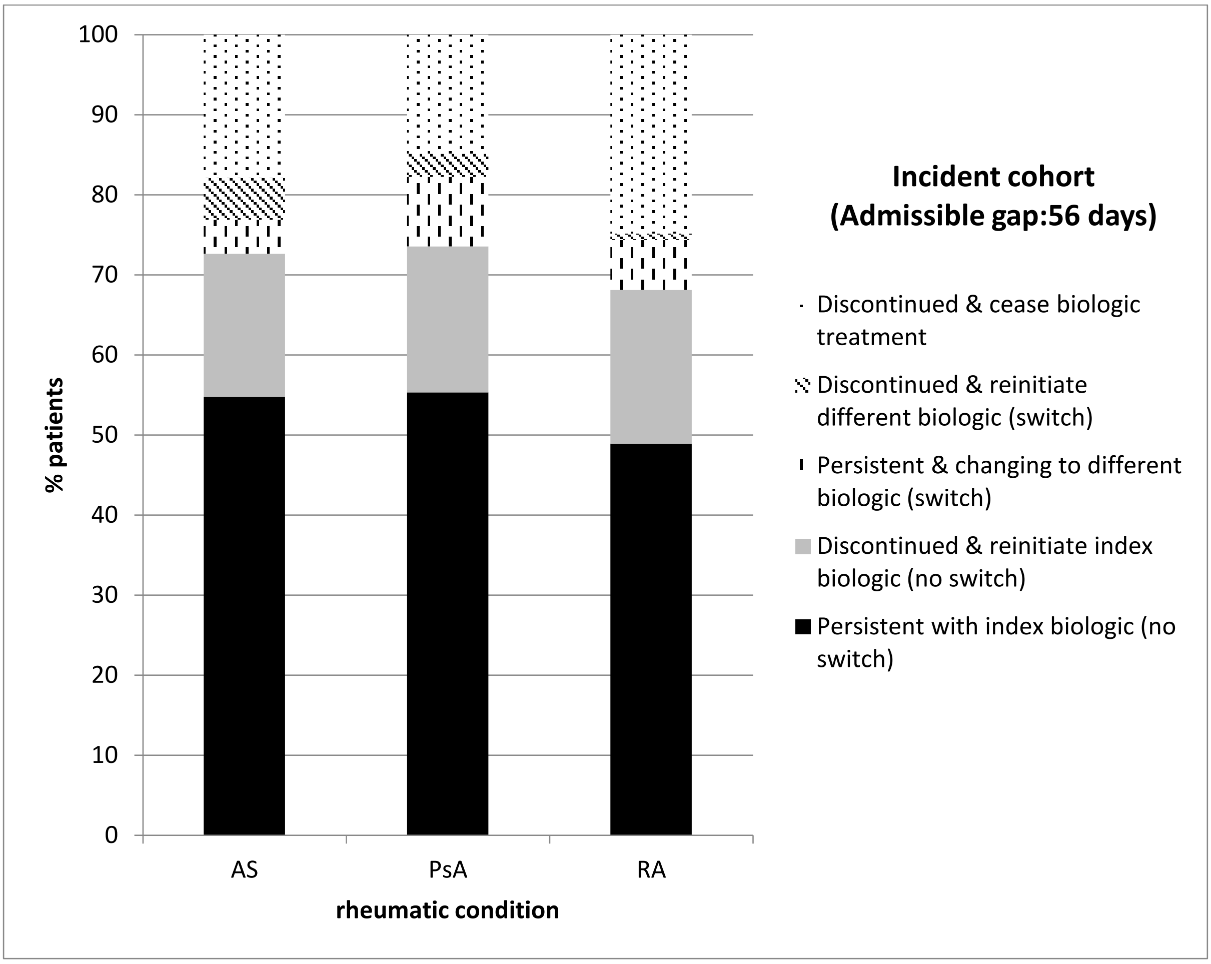

For peer review only - http://bmjopen.bmj.com/site/about/guidelines.xhtml

Sensitivity analyses discontinuation and persistence

Supplementary Figure 3. Persistence and discontinuation (re-initiating or ceasing) of treatment with biologics according to rheumatic condition in all the cohort with a gap of 28 days (Fig.6A) or 84 days (Fig.6B) and in the incident cohort with a gap of 28 days (Fig.6C) or 84 days (Fig.6D)

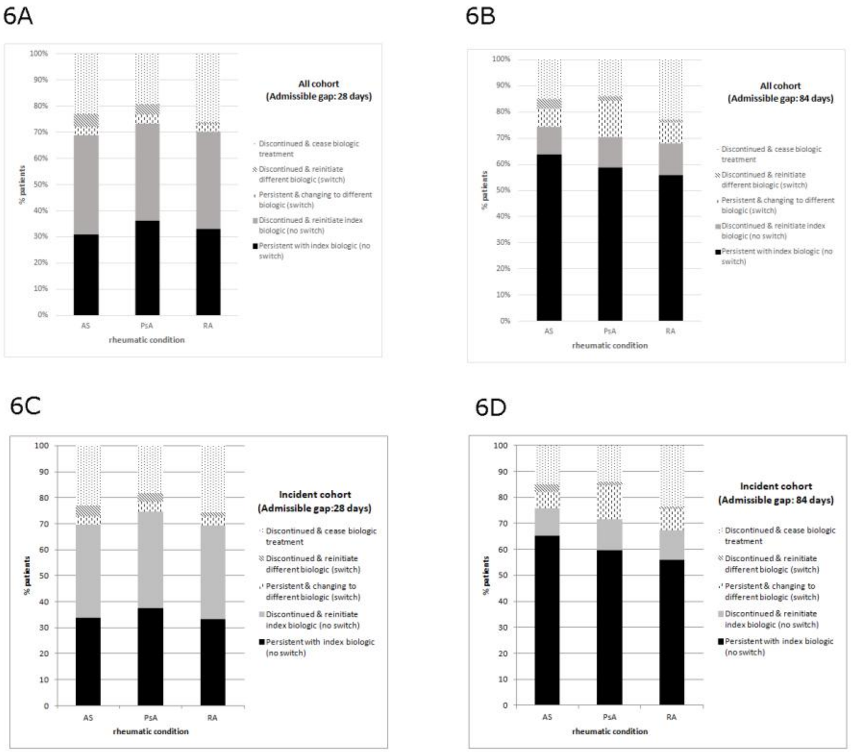

only

The crude survival curve of *time to discontinuation* (first gap greater than 56 days between biologic deliveries) of any biologic in the incident is shown in Supplementary Figure 4. In the crude model, the proportion of patients persisting is also slightly higher in PsA and AS compared to RA; however, there is no significant difference according to the log-rank test (p=0.165) and the Cox regression between RA and PsA (HR =0.77, 95%CI:0.58-1.01, p=0.0597).

Supplementary Figure 4:Crude survival curves comparing persistent patients according to rheumatic condition. (n=577)

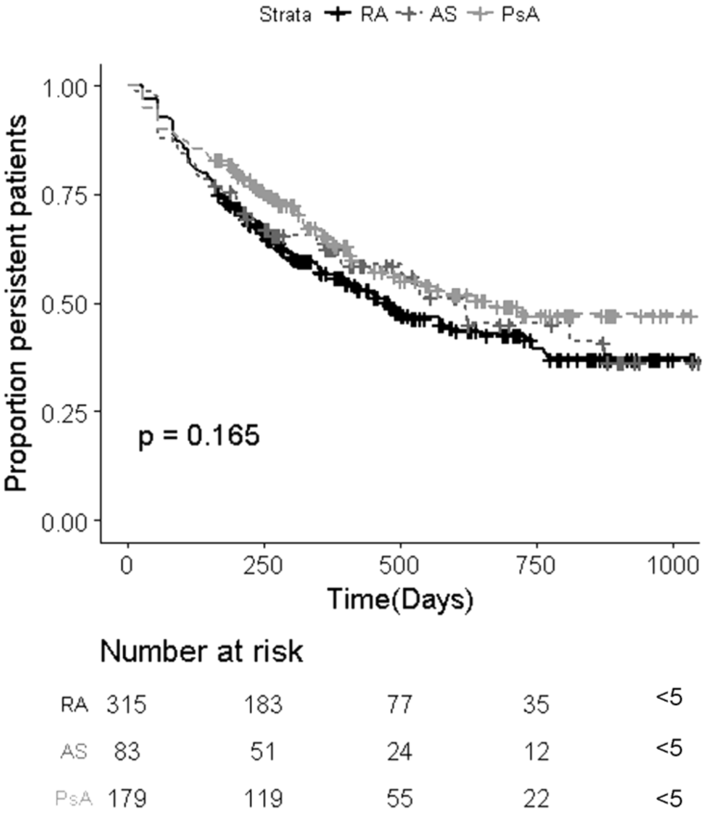

After adjustment for covariates, the difference between RA and PsA patients was still present (HR =0.73, 95%CI:0.54-1.00, p=0.048). Fewer covariates were significant in the incident cohort model. SIMD 3 was associated with a higher risk of discontinuation, whereas SIMD 5 (least deprived quintile) was associated with a lower risk of discontinuation as shown in Supplementary Figure 5. Sex, age and the use of methotrexate or DMARD prior or during the study were not statistically significant covariates.

For peer review only - http://bmjopen.bmj.com/site/about/guidelines.xhtml

Supplementary Figure 5: Adjusted Hazard Ratios for persistence with biologics using Cox regression analysis excluding patients without SIMD values (n=556)

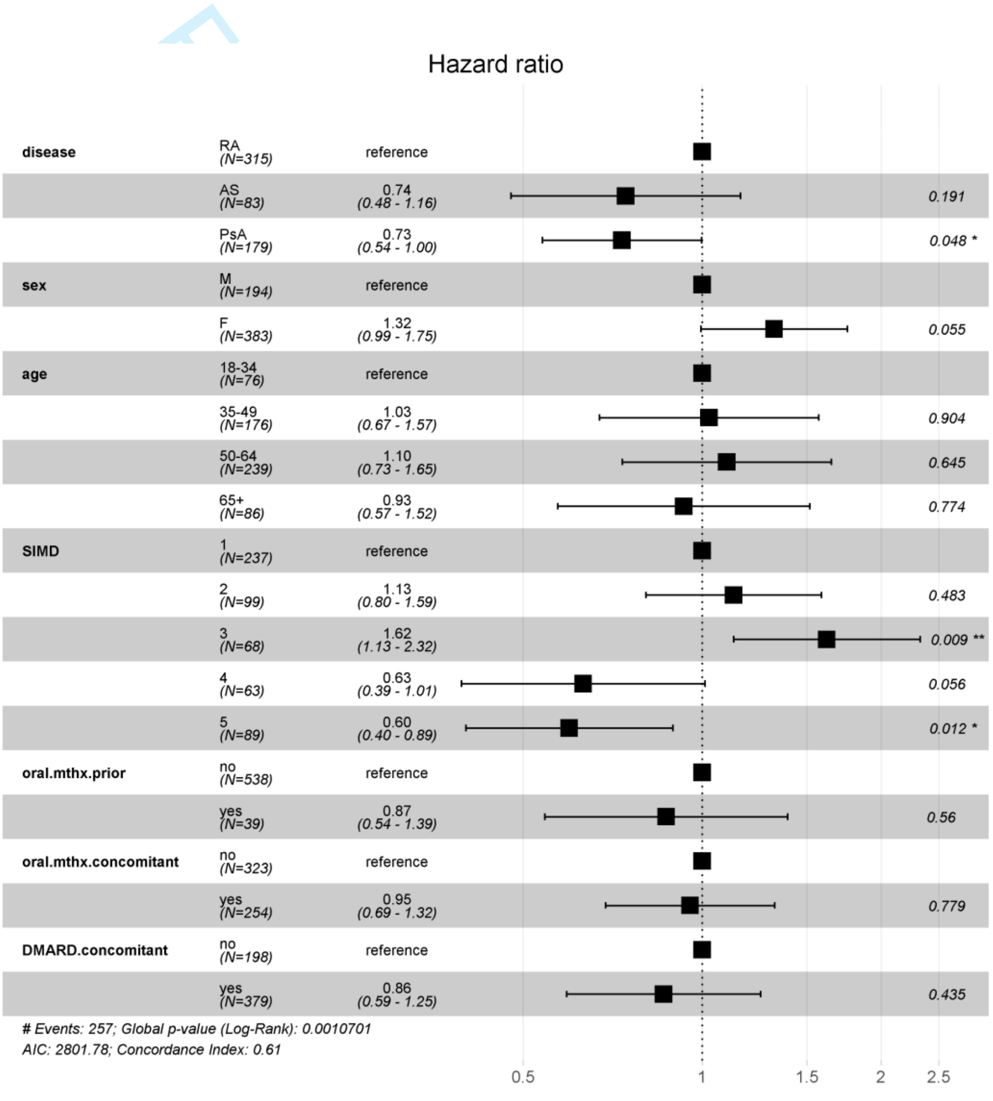

For peer review only - http://bmjopen.bmj.com/site/about/guidelines.xhtml

Adherence

Incident patients with at least two deliveries of the first (index) biologic (n = 598, 88.9%) were used to evaluate adherence. With overall adherence over 80% and both MRA and CR results showing the same patterns as the overall cohort.

Supplementary Figure 6: Adherence to biologics by rheumatic condition according to %MRA (5A) and %CR (5B). MRA- medication refill adherence; CR- compliance rate.

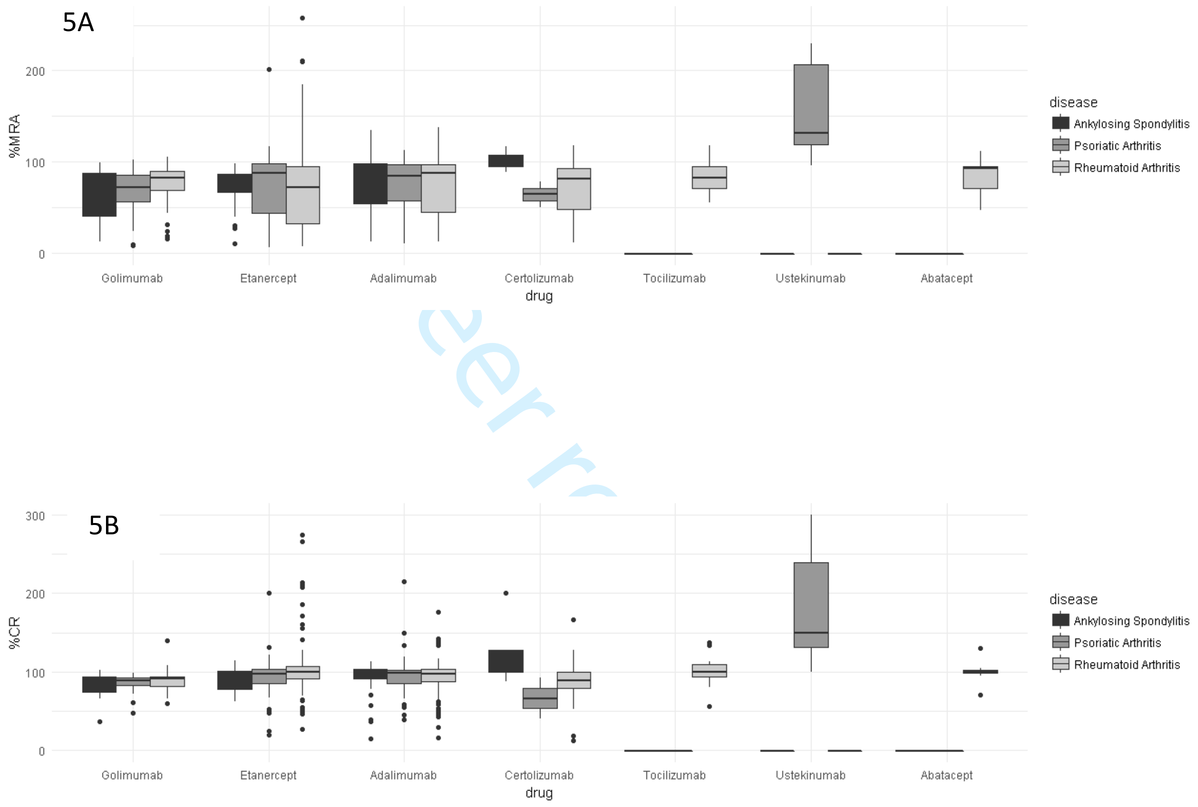

For peer review only - http://bmjopen.bmj.com/site/about/guidelines.xhtml

References

1. Widdifield J, Labrecque J, Lix L, et al. Systematic review and critical appraisal of validation studies to identify rheumatic diseases in health administrative databases. *Arthritis Care Res* 2013; **65**(9): 1490-503.
2. Herrett E, Thomas SL, Schoonen WM,et al. Validation and validity of diagnoses in the General Practice Research Database: a systematic review. *Br J Clin Pharmacol* 2010; **69**(1): 4-14.
3. Dubreuil M, Peloquin C, Zhang Y,et al. Validity of Ankylosing Spondylitis Diagnoses in The Health Improvement Network. *Pharmacoepidemiol Drug Saf* 2016; **25**(4): 399-404.
4. Thomas SL, Edwards CJ, Smeeth L, et al. How accurate are diagnoses for rheumatoid arthritis and juvenile idiopathic arthritis in the general practice research database? *Arthritis Rheum* 2008; **59**(9): 1314-21.

For peer review only

For peer review only - http://bmjopen.bmj.com/site/about/guidelines.xhtml

STROBE 2007 (v4) Statement—Checklist of items that should be included in reports of *cohort studies*

Section/Topic	Item #	Recommendation	Reported on page #
**Title and abstract**	1	(*a*) Indicate the study's design with a commonly used term in the title or the abstract	1
		(*b*) Provide in the abstract an informative and balanced summary of what was done and what was found	2
**Introduction**			
Background/rationale	2	Explain the scientific background and rationale for the investigation being reported	3
Objectives	3	State specific objectives, including any prespecified hypotheses	3
**Methods**			
Study design	4	Present key elements of study design early in the paper	4
Setting	5	Describe the setting, locations, and relevant dates, including periods of recruitment, exposure, follow-up, and data collection	4-5
Participants	6	(*a*) Give the eligibility criteria, and the sources and methods of selection of participants. Describe methods of follow-up	4-5
		(*b*) For matched studies, give matching criteria and number of exposed and unexposed	NA
Variables	7	Clearly define all outcomes, exposures, predictors, potential confounders, and effect modifiers. Give diagnostic criteria, if applicable	5-6
Data sources/ measurement	8*	For each variable of interest, give sources of data and details of methods of assessment (measurement). Describe comparability of assessment methods if there is more than one group	4-6
Bias	9	Describe any efforts to address potential sources of bias	13
Study size	10	Explain how the study size was arrived at	4
Quantitative variables	11	Explain how quantitative variables were handled in the analyses. If applicable, describe which groupings were chosen and why	5
Statistical methods	12	(*a*) Describe all statistical methods, including those used to control for confounding	5-6
		(*b*) Describe any methods used to examine subgroups and interactions	5-6
		(*c*) Explain how missing data were addressed	5
		(*d*) If applicable, explain how loss to follow-up was addressed	NA
		(*e*) Describe any sensitivity analyses	4-5, supplementary file

For peer review only

**Results**			
Participants	13*	(a) Report numbers of individuals at each stage of study—eg numbers potentially eligible, examined for eligibility, confirmed eligible, included in the study, completing follow-up, and analysed	4
		(b) Give reasons for non-participation at each stage	4
		(c) Consider use of a flow diagram	4
Descriptive data	14*	(a) Give characteristics of study participants (eg demographic, clinical, social) and information on exposures and potential confounders	6-9
		(b) Indicate number of participants with missing data for each variable of interest	7-9
		(c) Summarise follow-up time (eg, average and total amount)	8
Outcome data	15*	Report numbers of outcome events or summary measures over time	10
Main results	16	(*a*) Give unadjusted estimates and, if applicable, confounder-adjusted estimates and their precision (eg, 95% confidence interval). Make clear which confounders were adjusted for and why they were included	10-11
		(*b*) Report category boundaries when continuous variables were categorized	7-9
		(*c*) If relevant, consider translating estimates of relative risk into absolute risk for a meaningful time period	NA
Other analyses	17	Report other analyses done—eg analyses of subgroups and interactions, and sensitivity analyses	10-11, supplementary file
**Discussion**			
Key results	18	Summarise key results with reference to study objectives	11
**Limitations**			
Interpretation	20	Give a cautious overall interpretation of results considering objectives, limitations, multiplicity of analyses, results from similar studies, and other relevant evidence	12-14
Generalisability	21	Discuss the generalisability (external validity) of the study results	13
**Other information**			
Funding	22	Give the source of funding and the role of the funders for the present study and, if applicable, for the original study on which the present article is based	17

*Give information separately for cases and controls in case-control studies and, if applicable, for exposed and unexposed groups in cohort and cross-sectional studies.

For peer review only

Note: An Explanation and Elaboration article discusses each checklist item and gives methodological background and published examples of transparent reporting. The STROBE checklist is best used in conjunction with this article (freely available on the Web sites of PLoS Medicine at http://www.plosmedicine.org/, Annals of Internal Medicine at http://www.annals.org/, and Epidemiology at http://www.epidem.com/). Information on the STROBE Initiative is available at www.strobe-statement.org.

For peer review only
